# Retailer’s inventory-based financing with bounded advance rates: Interplay between wholesale price contract and loan menu

**DOI:** 10.1371/journal.pone.0347675

**Published:** 2026-07-30

**Authors:** Yaru Dang, Junfeng Tian, Junjie Liao, Meixian Tang, Chenyu Tian

**Affiliations:** 1 School of Business Administration, Faculty of Business Administration, Southwestern University of Finance and Economics, Chengdu, China; 2 New York University Shanghai, Shanghai, China; Ningbo China Institute for Supply Chain Innovation, UNITED STATES OF AMERICA

## Abstract

We examine a supply chain comprising a capital-constrained retailer, a supplier, and a bank. The retailer adopts inventory-based financing with bounded advance rates (IBF-B) to procure products through wholesale price contracts offered by the supplier. The bank is viewed as a strategic decision-maker with market power over the retailer when designing a loan menu that includes interest rates and inventory advance rates. By analyzing how inventory advance rates influence the retailer’s financing decisions, we derive upper and lower bounds for these rates to balance profitability and credit risk. Furthermore, we explore the interplay between the loan menu and wholesale price contracts. For the retailer intending to borrow up to the loan limit, the highest feasible wholesale price is offered if the supplier can obtain more profits, while the bank consistently charges the highest feasible interest rate. The upper bound of inventory advance rates is offered only when it yields a positive margin; otherwise, the interior advance rate within medium range is preferred. Numerical results illustrate the value of IBF-B, the motivations of all participants, and the impact of key economic parameters on financing decisions and supply chain performance.

## 1. Introduction

Small and medium-sized enterprises (SMEs) often face challenges in securing funding. In November 2018, 49.8% of small business loan applications were denied by banks in the United States [1]. One of the main reasons is that compared to mature and larger enterprises, SMEs have weak financial strength and limited disclosed financial information. It is a great challenge for banks to accurately assess SMEs’ credit and growth prospects. Besides traditional bank financing, trade credit financing has been well-accepted in supply chain transactions. SMEs, however, have insufficient bargaining power to obtain favorable trade credit [2]. To tackle the funding problem, asset-based lending (ABL), a financing option in which the lender provides loans secured by the borrower’s assets as collateral, presents a viable solution for SMEs seeking capital. ABL enables SMEs to leverage their assets, including receivables, inventory, or equipment, as collateral to obtain loans, thereby overcoming the challenges of obtaining funds on the basis of creditworthiness or cash flow [3]. For example, a Toronto-based industrial parts distributor secured a $2.3 million asset-based lending line of credit to keep pace with its marketing expansion [4].

In ABL, the lender can obtain repayment by liquidating the borrower’s assets if the borrower does not have sufficient funds to repay. Consequently, financial institutions focus on the assets’ liquidation value of SMEs with limited capital, and the ABL specialists should have strong experience in assessing the liquidation values of the inventory, receivables, and so on [4]. On the other hand, Hubert and Accardi [5] present that a key feature of ABL is the close relationship between lenders and borrowers’ management, particularly regarding working capital. To anticipate borrowers’ financing needs and ensure efficient loan utilization, the lender must remain informed about trends in both the borrowers’ businesses and industry-level developments. Moreover, the operational decisions of capital-constrained SMEs, which affect the lender’s financial decisions, are influenced by the upstream and downstream partners in the supply chain. Therefore, when designing loan contracts, the lender should evaluate the SMEs’ interplay with these partners to determine the value of assets. This interdependence motivates our study, which argues that the financial decisions of lenders should account for SMEs’ businesses with these partners.

As mentioned above, ABL is secured by firm assets, making asset valuation a core decision for lenders. To manage the credit risk, the lender adjusts not only interest rates but also asset advance rates, which typically range from 85–90% for accounts receivable and 50–75% for inventory [6]. The advance rate, expressed as a percentage of the expected asset liquidation value, reflects the lender’s assessment of the collateral’s value. For example, a 50% inventory advance rate indicates that the lender will extend credit up to half of the inventory liquidation value. In practice, inventory advance rates are typically subject to upper limits imposed by lenders to control credit risk. For example, Finimpact [7] and CFI Team [8] report inventory advance rates of around 50%, while Cerebro Capital [9] reports typical inventory advance rates ranging from 50% to 65%. These industry practices suggest that inventory-based financing (IBF) commonly operates under bounded inventory advance rates rather than unconstrained lending.

These industry practices motivate our research to investigate how the lender determines the bounds of the inventory advance rate based on the interactions among the lender, the capital-constrained firm, and the upstream supplier, which has not been well studied in the existing ABL literature. In particular, after identifying the feasible bounds of the inventory advance rate, we further examine how the bank optimally determines the advance rate within this bounded range.

To address this issue, our work considers a supply chain involving a bank, a capital-constrained retailer, and a supplier. As stated by Siskin [10], the retail inventory is among the most easily liquidated collateral assets on the balance sheet. This is reflected in business practice. For instance, a growing Canadian electronics retailer faces a classic inventory financing challenge that its available cash is not sufficient to cover the electronics inventory investment. In such cases, traditional banks view this retailer’s inventory as collateral to offer the loan [11]. Accordingly, the retailer employs inventory-based financing (IBF), a common form of ABL, to acquire products from the supplier. In case of a default, the bank recoups losses by liquidating these inventories. Because loan amounts depend on inventory value, the retailer should treat the bank’s lending decision as a capital constraint, while the bank should account for the retailer’s inventory decision when determining loan limits. Furthermore, the supplier’s non-negotiable wholesale price contract also affects the retailer’s ordering and credit decisions.

The retailer, like a newsvendor, procures products from the supplier to meet uncertain single-period demand, seeking to maximize expected final cash flow. The bank provides this retailer with a loan menu specifying a bounded inventory advance rate and an interest rate, with funds paid directly to ensure timely product delivery. This financing mechanism is hereinafter referred to as inventory-based financing with bounded advance rates (IBF-B). Since the decisions cannot be made independently for supply chain participants, including the bank, we focus on the following issues.

(1) How does the bank set the bounds of the inventory advance rate, considering the retailer’s ordering decision in IBF-B?
(2) How should the retailer with constrained capital determine orders and loans?
(3) How does the supplier choose the optimal wholesale price contract?
(4) What loan menu should the bank offer to the retailer?
(5) How does IBF-B with a loan limit affect supply chain performance, and what is its value?

### 1.1. Main results and contributions

This study examines inventory-based financing with bounded advance rates (IBF-B) in a supply chain consisting of a bank, a supplier, and a capital-constrained retailer. The bank possesses substantial market power over the retailer belonging to SMEs with constrained working capital and is seen as a risk-neutral strategic player that offers the retailer a loan menu consisting of interest rates and inventory advance rates. We consider a three-level Stackelberg game in which the bank acts as the overall leader, the supplier acts as the sub-leader, and the retailer is the follower. The supplier offers a wholesale price contract, while the retailer determines whether to adopt IBF-B and how much to order. It is supposed that the supplier can receive all payments from the retailer at the first stage and make production decisions. However, the bank will loss a portion of the repayment if the retailer goes bankrupt. This paper derives the following main conclusions.

First, the bank can balance credit risk and profitability in IBF by adjusting not only the interest rate but also the inventory advance rate. By analyzing how the inventory advance rate affects the retailer’s financial risk based on his working capital, we suggest that the bank should set an inventory advance rate within the medium range, defined by the lower and upper bounds, to mitigate the retailer’s default risk and be more profitable, which has not been explored in the existing studies of IBF [12–14]. This result aligns with the business practice mentioned above, where inventory advance rates are typically bounded rather than unconstrained.

Second, based on the retailer’s financing needs, we examine how the supplier determines the wholesale price contract and how the bank sets the loan menus, respectively. The supplier can offer tailor-made wholesale prices to various retailers according to their working capital level. For retailers intending to borrow up to the loan limit and facing bankruptcy risk, the supplier will make a trade-off between a wholesale price contract that permits borrowing up to the loan limit and one that relies solely on working capital, thereby achieving higher profit. Since the bank has strong market power over SMEs, in the context of IBF-B, it is viable to simultaneously optimize profits and mitigate the retailers’ default risk by setting both interest rates and inventory advance rates within its bounds. And we find that the order quantity of the retailer who borrows up to the loan limit depends only on the inventory advance rate rather than the interest rate. For a given wholesale price, intuitively, when an inventory advance rate yields a positive marginal contribution to the bank’s profit, the bank prefers to set it as high as possible until the upper bound of a medium range. Otherwise, if the inventory advance rate does not generate a positive marginal contribution, the bank should select the interior advance rate within the medium range. Meanwhile, the bank consistently charges the highest feasible interest rate to manage the credit risk of retailers borrowing up to the loan limit. All of these insights are not embodied in extant research (e.g., Dada and Hu [15]).

Finally, we explore the interplay between the wholesale price contract and the loan menu, and assess the value of IBF-B by analyzing its impact on supply chain performance under a uniform demand distribution. The structure of the bank’s loan menu decisions, based on the supplier’s wholesale price, remains similar to that when the wholesale price is fixed. Thus, the essence of the bank’s decisions does not change whether the wholesale price is endogenous or not. This finding further supports the rationale for designing the loan menu based on the wholesale price, which is consistent with the judicial practice in IBF-related cases, where courts treat the wholesale price as the value of the collateral [16,17]. It is also shown that the bank raises inventory advance rates to satisfy as much of the loan demand of the poorer retailers who borrow up to the loan limit, while simultaneously charging higher interest rates to manage credit risk. To stimulate purchase, the supplier sets lower wholesale prices for these retailers. The numerical results further indicate that, for the retailers with less working capital and attempting to borrow up to the loan limit, the supplier offers the wholesale price contract that enables them to place orders using only working capital instead of adopting IBF-B. However, retailers who intend to borrow up to the loan limit but have more working capital can be permitted to order through IBF-B. The bank sets the upper bound of inventory advance rates to these retailers, which can effectively balance the profitability and the retailer’s bankruptcy risk. The fact is aligned with real-world practice. It is implied that restricting the inventory advance rate within a medium range is an effective strategy. The retailer who borrows up to the loan limit is poor and obtains a sizable loan, which in turn leads to a remarkable enhancement in supply chain performance. Furthermore, IBF-B significantly boosts overall supply chain profitability. Sensitivity analyses further reveal that inventory recoverability, demand conditions, and the bank’s loan costs play an important role in determining the bank’s loan menu and supply chain performance.

Our paper makes three key contributions to the existing literature. First, we fill in the gap of extant studies on IBF by obtaining the inventory advance rate bounds that effectively balance the profitability and credit risk. Second, we provide a rigorous analysis of the decision of supply chain participants, including a retailer, a supplier, and a bank, and explore the interplay between loan menus and wholesale price contracts by examining how the capital-constrained retailer should employ IBF-B for procurement. Finally, our interpretations of the order quantity and wholesale price between the retailer and supplier, as well as the bank’s loan menu towards the retailer, propose interesting insights on how IBF-B affects the three parties’ decisions and whether IBF-B is profitable for the supply chain.

### 1.2. Paper structure

The paper is organized as follows. Section 2 reviews the relevant literature. Section 3 provides a detailed introduction to our model. Section 4 discusses the retailer’s ordering decision under IBF-B. Section 5 derives the analytical equilibrium solutions of the supplier and the retailer. Section 6 presents the loan terms that the bank offers to the capital-constrained retailer. Section 7 conducts extended discussions to analyze the interplay between the wholesale price contract and loan menu under a uniform demand distribution, evaluates the impact of IBF-B on supply chain performance, and investigates the effects of key economic parameters on financing and operational decisions. Finally, Section 8 concludes the paper and provides managerial insights. Proofs for all lemmas and propositions are stated in S1 Appendix (S1 File).

## 2. Literature review

Our paper involves the interfaces of operations and finance, particularly inventory financing. One stream of this area studies the interaction between operational decisions and capital structure. Xu and Birge [18] are the pioneers to integrate the production decisions with the capital structure choices and examine how a capital-constrained firm in an imperfect market balances the tax-shield benefits of debt with the bankruptcy costs when making production and financing decisions. Then, Hu and Sobel [19] study the optimal short-term inventory and debt decisions, and discuss the long-term capital structure under firm value maximization. Li et al. [20] point out that studying short-term debt can reveal the consequences of default risk on a firm’s inventory decisions by examining a dynamic coordination model for an equity-financed firm. Moreover, Rzeszutek et al. [21] utilize the agent-based model to investigate the impact of overconfidence on capital structure.

While the above studies pay more attention to long-term financial strategies for inventory financing, another stream studies the impact of short-term financing formats, including trade credit, bank financing, and asset-based lending (ABL), on inventory decisions of capital-constrained firms. Trade credit, an agreement allowing customers to defer payment for purchased products, has been widely analyzed in conjunction with operational decisions, with a thorough review of the literature provided by Seifert et al. [22]. There are considerable studies shown as follows that examine the effects of trade credit, bank financing, and their combination on firms’ operational decisions. Notable studies include Zhou and Zhou [23], who present that the supplier offers trade credit to induce the retailer to order under certain demand; Gupta and Wang [24] examine the impact of dated-related trade credit on the retailer’s inventory decisions under uncertain demand, deriving an optimal inventory decision that does not depend on the trade credit rate. Kouvelis and Zhao [25] present the equilibrium solutions for a Stackelberg game involving a well-capitalized supplier and a retailer facing capital constraints under uncertain demand, where the retailer relies on bank financing to obtain funds. Then, Yang and Birge [26], Lu and Wu [27], and Yang et al. [28] analyze trade credit and bank financing to identify which is more advantageous for capital-constrained firms. There is also an empirical study that examines how trade credit affects inventory decisions, demonstrating that trade credit serves as a critical source of funding for inventory investment [29].

In extant literature, bank loans can be assumed to be competitively priced, which means that the bank has no preference between lending to borrowers and allocating funds to risk-free investments. Under this assumption, capital-constrained retailers generally prefer trade credit over bank financing [30–32]. On the other hand, some studies treat banks as decision-makers or leaders. For instance, Dada and Hu [15] and Yan et al. [33] model Stackelberg games in which the bank acts as a leader seeking to maximize profits, while the capital-constrained retailer follows. In the research on ABL, Buzacott and Zhang [13] are the first to integrate ABL into inventory investment and analyze the optimal credit decision made by the bank; Alan and Gaur [12] present how the bank determines loan menus of ABL based on a retailer’s inventory to maximize its profits, in which the retailer decides the stock level and capital structure, and show how the capital structure influences operational decisions. Also, our study aligns with the practical situation of SMEs using IBF, where such firms have weak market power [2]. The Bank is dominant and therefore, is a strategic decision maker rather than seeking risk-free returns through competitively priced loans. And to control the credit risk of IBF, the bank determines the optimal inventory advance rate within preset bounds.

Apart from the impact of financing decisions on operations, the pricing decisions made by upstream suppliers in the supply chain affect the ordering choices of downstream capital-constrained retailers, which in turn affects the bank’s IBF or ABL loan menu. For example, Jiang and Liu [34] investigate how the bank determines the inventory advance rate based on the wholesale price contract offered by a supplier who is overconfident in the demand forecasting. In their setting, the retailer’s working capital is not explicitly considered, and the bank’s loan interest rate is exogenously given. Also, the loan amount is assumed to equal the loan limit determined by the inventory advance rate. In contrast, our work explicitly incorporates the retailer’s working capital into the financing decision and simultaneously considers the IBF-B loan menu, consisting of the interest rate and the bounded inventory advance rate, together with the wholesale price contract provided by a rational supplier. Different working capital levels lead to different retailer types with distinct loan demands and bankruptcy risks. Furthermore, when the interest rate is fixed, the bank can only adjust the inventory advance rate to control credit risk, which may lead to either overly restricting financing or excessive default exposure. By jointly optimizing both the interest rate and inventory advance rate, the bank can balance profitability and credit risk. Specifically, the bank offers a higher inventory advance rate to encourage retailers with lower working capital to place larger orders, while simultaneously charging a higher interest rate to compensate for the greater credit risk associated with such retailers. Furthermore, due to the implicit solutions in our work, we numerically show the interplay between wholesale price contracts and loan menus, the impact of IBF-B on supply chain performance, as well as the impact of key economic parameters on loan menus and supply chain performance.

Unlike previous studies, Zhou and Groenevelt [14], which treat the manufacturer as the leader in a Stackelberg game and examine how a capital-constrained retailer, as the follower, utilizes IBF and open accounting finance to achieve optimal inventory under the competitively priced bank loans, our paper considers that the bank has market power over SMEs and can price loans, with the supplier providing only a wholesale price contract without financing support.

Some studies further investigate IBF in dynamic inventory settings. For example, Fu et al. [35] study a multi-period inventory model for a capital-constrained firm with access to IBF and show that the firm may strategically overstock inventory in early periods to secure future financing. Liang et al. [36] examine dynamic inventory policy under IBF by jointly considering stochastic material prices and uncertain demand. Moreover, Chen and Zhu [37] study retailer-initiated IBF and derive the lender’s optimal interest rate and the borrower’s pledging strategy, while comparing IBF with the Future Goods Financing scheme. However, these studies either focus on dynamic inventory decisions or treat only one financing parameter as endogenous. In contrast, our study jointly determines the interest rate and inventory advance rate while examining the interactions between the financing and operational decisions. Furthermore, Song et al. [38] investigate IBF as a contingent financing mechanism to hedge retailers against future liquidity shortage after inventory procurement and employ blockchain-based smart contracts to make IBF adoption contractible.

To the best of our knowledge, no prior research explores the inventory advance rate bounds in IBF for capital-constrained retailers and analyzes how a well-capitalized supplier sets wholesale prices to such retailers who leverage IBF-B to obtain limited funds. Moreover, we consider the fact that banks strive for maximum profits based on the bounds of inventory advance rates, and examine the interplay between wholesale price contracts and loan menus (including interest rates and inventory advance rates). This paper contributes to a better understanding of *inventory-based financing with bounded advance rates* by viewing the bank as a strategic player with market power over capital-constrained retailers as SMEs. The distinctions between our research and closely related literature are shown in Table 1.

**Table 1:** The distinctions between this paper and closely related literature.

	Exploring the inventory advance rate bound of IBF	Lender’s decisions	Lender is a strategic decision maker	Using IBF to obtain funding
Dada and Hu [15]		R	√	
Buzacott and Zhang [13]		R	√	√
Zhou and Groenevelt [14]		R		√
Alan and Gaur [12]		R and β	√	√
Jiang and Liu [34]		β	√	√
Yan et al. [33]		*R*	√	
Chen and Zhu [37]		*R*	√	√
This paper	√	R and β	√	√

*Note:* R *denotes the interest rate and* β *denotes the inventory advance rate. √ means that the research considers this term.*

## 3. Model setting

### 3.1 Basic description

We consider a three-level Stackelberg game in which the bank (“it”) serves as the overall leader, the supplier (“she”) acts as the sub-leader, and the capital-constrained retailer (“he”), representing a small and medium-sized enterprise (SME), is the follower. The bank first announces a loan menu comprising an interest rate R and an inventory advance rate β, conditional on the retailer’s working capital k. The supplier then offers the retailer a non-negotiable wholesale price w. Observing the loan menu and wholesale price, the retailer acting as a newsvendor decides whether to adopt inventory-based financing with bounded advance rate (IBF-B), a form of ABL, and determines the one-time order quantity q, based on his working capital.

Aligning with the statement of ABL [39], which indicates that purchased inventory can serve as collateral, we consider that the retailer’s current-period purchased inventory is used as collateral for the bank loan. The collateral valuation is tied to the inventory procurement value. Also, Zachary Scott [40], a boutique investment banking firm, states that ABL lenders can prepay inventory or accounts receivable to ensure borrowers have adequate working capital to purchase products. Therefore, we consider that if the retailer signs a loan contract, the bank will disburse this loan directly to the supplier, while the retailer covers the remaining balance from working capital. After receiving the payment, the supplier delivers products to a third-party logistics (3PL) company entrusted by the bank to monitor this collateral. The bank then incurs a monitoring cost at rate $r_{c}$, paid to the 3PL, and requires the retailer to deposit sales revenue into a closed account designated for loan repayment.

Once demand is realized, the retailer repays the bank loan in full if revenue is adequate. Otherwise, he files for bankruptcy, and the bank seizes all of his remaining assets. For simplicity and without loss of generality, we consider the continuous compounding over the loan term and express the unit loan return as $e^{R}$. Any remaining working capital after using is invested in a risk-free market at an interest rate $r_{f}$. The unit selling price and salvage value are denoted by p and s, respectively, while c represents the supplier’s unit production cost. The wholesale price w represents the inventory procurement value and serves as the basis for collateral valuation, whereas the salvage value s denotes the liquidation recovery value of unsold inventory. Consistent with standard newsvendor setting, we assume s<w<p. In IBF-B, the bank has a legal right to liquidate the retailer’s inventory and recover the salvage value s in the event of default. We assume no goodwill loss from unmet demand. Note that if $we^{R}\left(or\;we^{r_{f}}\right)>p$, the retailer has no incentive to order, and if c>w, the supplier will not produce. To avoid trivial cases, let $p\geq we^{R}\geq ce^{R}\geq se^{R}$.

Demand is represented as a random variable ξ≥0 with probability density function (PDF) f(·), cumulative distribution function (CDF) F(·) and complementary CDF $\overset{―}{F}(\cdot )$. Assume that F(·) is differentiable, increasing and satisfies F(0)=0. The failure rate is denoted as $z(\cdot )=\frac{f(\cdot )}{\overset{―}{F}(\cdot )}$. Following the existing literature [12,41], we assume a demand distribution with an increasing failure rate (IFR). In other words, for $\text{0}\leq \xi_{\text{1}}\leq \xi_{\text{2}}<\infty$, $\text{0}\leq z\left(\xi_{\text{1}}\right)\leq z\left(\xi_{\text{2}}\right)$ always holds. Let $v_{r}$, $\pi_{s}$ and $\pi_{b}$ represent the retailer’s final cash flow, the supplier’s profits and the bank’s profits, respectively.

The fundamental assumptions of the model are outlined below.

**Assumption 1:** The retailer, supplier and bank are all risk-neutral, and no asymmetric information exists regarding the company and investment choices. All parameters are common knowledge [32,42]. The bank incurs monitoring costs paid to the 3PL, representing a form of financial friction [43].

**Assumption 2:** Both the retailer and supplier are limited liability firms, and their long-term capital structures are entirely equity-financed. The retailer is creditworthy and repays the bank loans (if any) to extent possible.

**Assumption 3:** The financial economics literature highlights the bank’s market power ranging from monopoly to perfect competition [44–46]. Since focusing on SMEs, it is reasonable to believe that the bank has strong market power and is in a monopolistic status, consistent with the mainstream studies [12,13,15,47]. Accordingly, the supply chain is modeled as a three-level Stackelberg game in which the bank acts as the overall leader, the supplier acts as the sub-leader, and the capital-constrained retailer is a follower.

We summarize the main notations in Table 2.

**Table 2:** Notations.

Expression	Description
q	Retailer’s order quantity.
p	Retailer’s selling price.
w	Supplier’s wholesale price, $we^{R}\leq p$.
c	Supplier’s unit production cost, c≤w.
s	Unit salvage price, s≤c.
ξ	Uncertain demand (random variable).
R	Bank’s interest rate, $r_{c}\leq R$.
β	Inventory advance rate for inventory-based financing, 0≤β≤1.
$r_{c}$	Bank’s loan cost rate in inventory-based financing, $r_{f}\leq r_{c}\leq \text{1}$.
$r_{f}$	Risk-free interest rate.
k	Retailer’s working capital.
$v_{r}$	Retailer’s final cash position.
$\pi_{i}$	i=s,b, supplier’s profits and bank’s profits, respectively.
f(·)	Probability density function of demand distribution.
$\overset{―}{F}(\cdot )$	Complementary cumulative distribution function of demand distribution.
z(·)	Failure rate of demand distribution，z$\left(\cdot )=f(\cdot )/\overset{―}{F}(\cdot \right)$.

### 3.2 Decision sequence and methodology

**Fig 1:**
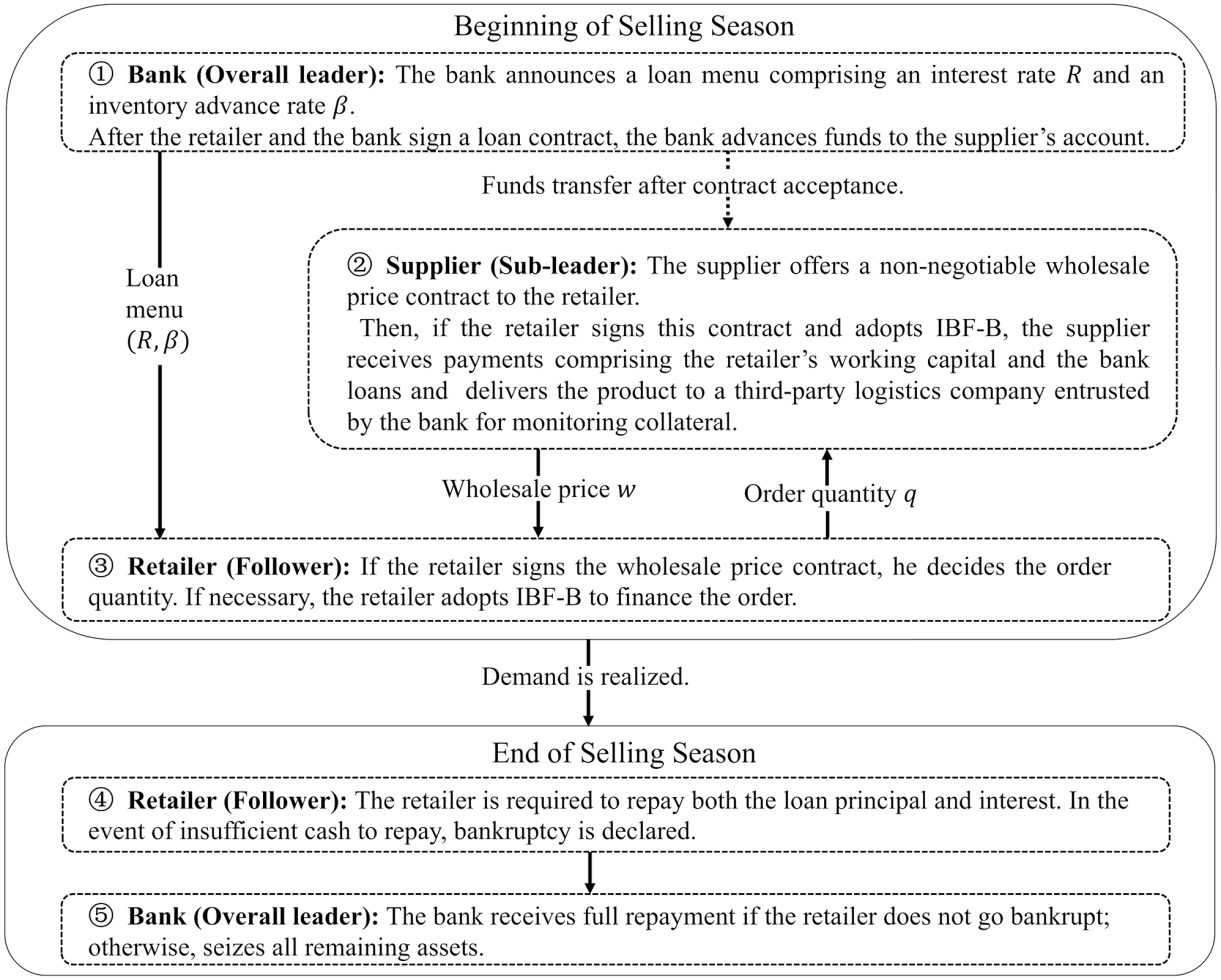
Decision sequence of events.

The sequence of decision-making events is depicted in Fig 1. At the beginning of the selling season, the bank announces the loan terms, including interest rates R and inventory advance rates β. The supplier then sets the wholesale price w for the retailer. Observing the loan terms and wholesale price, the retailer determines the order quantity q and decides whether to adopt IBF-B to maximize his expected profits. Once the loan contract is accepted, the bank disburses funds directly to the supplier, who subsequently delivers the products to a third-party logistics company entrusted by the bank for monitoring. At the end of the selling season, the retailer is required to fulfill his repayment obligations. In the event of bankruptcy, the bank seizes all remaining assets.

Methodologically, we adopt a newsvendor model to describe the inventory investment problem under demand uncertainty and solve it by backward induction since the decisions are sequential. We first analyze the retailer’s optimal order quantity $q_{r}^{*}$ for various working capital cutoffs k(w) and examine the impacts of bank loan terms (i.e., R and β) on $q_{r}^{*}$. Then we derive the equilibrium wholesale price $w^{*}$ for the supplier within a Stackelberg game framework, accounting for the correlation between w and k(w). To determine optimal bank loan terms, we further demonstrate the relationship between the interest rate R and k(R). Due to the complexity of the model, we first examine the bank’s loan contract decisions under a fixed wholesale price in general demand distribution. We then extend the analysis to a given demand distribution to derive the equilibrium decisions of supply chain participants and explore the interaction between the wholesale price contract and the loan menu. Finally, numerical results demonstrate the impacts of IBF-B on supply chain performance and reveal the consistency with business practices.

## 4. Retailer’s decisions under IBF-B

### 4.1 Retailer’s expected return

Considering that the retailer does not have adequate working capital to cover the total purchase cost, he pledges the inventory to obtain funds from the bank. The bank determines the loan contract consisting of the interest rate R and the inventory advance rate β, which limits the loan amount. Consequently, the retailer’s loan demand $l(q)=(wq-k{)}^{+}$ is constrained by the loan limit βwq, which is consistent with Zhou and Groenevelt [14] and Alan and Gaur [12], where $(a{)}^{+}=max\left\{a,\text{0}\right\}$. The analysis in Section 5 shows that the optimal wholesale price correlates with the selling price, indicating that the loan limit βwq captures both the purchase cost and market price of the product. Let $q_{\text{0}}=\frac{k}{(\text{1}-\beta )w}$ be *loan-limit order quantity* so that $wq_{\text{0}}-k=\beta wq_{\text{0}}$, meaning the borrowed amount exactly reaches the loan limit.

At a point of time, demand is realized. The retailer’s income comprises sales revenue pmin(ξ,q), the salvage value of unsold inventory $s(q-\xi {)}^{+}$, and capital investment $(k-wq{)}^{+}e^{r_{f}}$. Meanwhile, he has to fulfill his loan repayment obligations. Then, the retailer’s decision problem is


$$\begin{array}{c} v_{r}(q,\xi ;w;R,\beta )=[pmin(\xi ,q)+s(q-\xi {)}^{+}-l(q)e^{R}+{\left({k-wq)}^{+}e^{r_{f}}\right]}^{+}\\ \;\begin{array}{c} s.t.\;l(q)\leq \beta wq. \end{array} \end{array}$$ (1)

The retailer can fulfill all loan obligations if demand is sufficiently high. If not, the retailer declares bankruptcy and his cash and other assets are in possession of the bank. However, since the retailer has limited liability, his potential loss is only $ke^{r_{f}}$. Subtracting $ke^{r_{f}}$ from $v_{r}(q,\xi ;w;R,\beta )$ yields the retailer’s final profits. Therefore, maximizing the retailer’s final cash flow is equivalent to maximizing his final profits.

Considering that the retailer may go bankrupt if he adopts IBF-B, we first state the *order quantity threshold* in Lemma 1 and the *bankruptcy threshold* in Lemma 2 as follows to identify under what conditions bankruptcy occurs.

**Lemma 1.** *If* $\frac{k}{w}\leq q\leq \hat{q}$*, the retailer borrows without bankruptcy risk, where* $\hat{q}=\frac{k}{w(\text{1}-\gamma )}$ *is order quantity threshold and* $\gamma =\frac{s}{we^{R}}$*.*

If $\frac{k}{w}\leq q\leq \hat{q}$, the product’s salvage value adequately covers the loan obligations, so there is no bankruptcy risk on the retailer’s borrowing.

**Lemma 2.** *If the order quantity satisfies* $\hat{q}<q$*, the retailer goes bankrupt with* $\xi <\hat{\xi }(q)$*, where* $\hat{\xi }(q)=\frac{{\left[l(q)e^{R}-sq\right]}^{+}}{p-s}$*.*

Obviously, the bankruptcy threshold satisfies $\hat{\xi }(q)<q$ for $\hat{q}<q$. Lemma 2 also indicates that if demand exceeds the retailer’s order quantity, the retailer will avoid bankruptcy.

From Lemmas 1 and 2, we derive the retailer’s expected final cash flow. That is,


$$\begin{array}{c} E\left[v_{r}(q,\xi ;w;R,\beta )\right]=\left\{ \;\;\;\;\;\;\;\;\;\begin{array}{ccccc} & (p-s)\int_{\text{0}}^{q}\overset{―}{F}(\xi )d\xi -\left(we^{r_{f}}-s\right)q+ke^{r_{f}} & , & \text{0}\leq q<\frac{k}{w}\;(2a)\;\\ & (p-s)\int_{\text{0}}^{q}\overset{―}{F}(\xi )d\xi +sq & , & q=\frac{k}{w}\;(2b)\\ & (p-s)\int_{\text{0}}^{q}\overset{―}{F}(\xi )d\xi -\left(we^{R}-s\right)q+ke^{R} & , & \frac{k}{w}<q\leq \hat{q}\;(2c)\\ & (p-s)\int_{\hat{\xi }(q)}^{q}\overset{―}{F}(\xi )d\xi & , & \hat{q}<q. & \;\;\;\;\;\;\;\;\;\;\;\;\;\;\;\;\;(2d) \end{array}\right. \end{array}$$ (2)

### 4.2 Retailer’s ordering decision

The optimal order quantity for a retailer using IBF-B to alleviate capital constraints is subject to the loan limit, which is directly related to inventory advance rates that also affect the retailer’s bankruptcy risk. In this section, we begin by presenting the retailer’s bankruptcy risk exposure under various inventory advance rate ranges in Lemma 3.

**Lemma 3.** *For a given* R*,* w *and* k*, if the bank sets a lower inventory advance rate, i.e.,* 0≤β≤γ*, the retailer does not go bankrupt. If the bank sets a medium inventory advance rate, i.e.,* $\gamma <\beta \leq \hat{\beta }$*, the retailer borrows with bankruptcy risk. And if the bank sets a higher inventory advance rate, i.e.,* $\hat{\beta }<\beta \leq \text{1}$*, the retailer’s borrowing is at higher bankruptcy risk, where* $\hat{\beta }=\text{1}-\frac{q_{\text{2}}(w,R)\left(\text{1}-\gamma (w,R)\right)}{q_{\text{3}}(w,R,k)}$*,* $q_{\text{2}}(w,R)={\overset{―}{F}}^{-\text{1}}\left(\frac{we^{R}-s}{p-s}\right)$ *and* $q_{\text{3}}(w,R,k)={\overset{―}{F}}^{-\text{1}}\left(\frac{we^{R}-s}{p-s}\overset{―}{F}\left(\hat{\xi }\left(q_{\text{3}}(w,R,k)\right)\right)\right).$

Now, let the retailer’s expected final cash flow $E\left[v_{r}(q)\right]=\text{E}\left[v_{r}(q,\xi ;w;R,\beta )\right]$. Then, we derive the retailer’s optimal order quantity by calculating optimality conditions of $E\left[v_{r}(q)\right]$ with respect to q, as presented in Proposition 1.

**Proposition 1.** *For a demand distribution with IFR, given* w*,* R *and* $\gamma <\beta \leq \hat{\beta }$*, the retailer’s optimal order quantity that depends on his working capital, is*


$$\begin{array}{c} \begin{array}{c} q_{r}^{*}=\left\{ \begin{array}{cccc} & q_{\text{1}}(w) & , & if\;k\geq wq_{\text{1}},\;(no\;borrowing)\\ & \frac{k}{w} & , & if\;wq_{\text{2}}\leq k\leq wq_{\text{1}},\;(no\;borrowing,\;but\;using\;up\;working\;capital)\\ & q_{\text{2}}(w,R) & , & if\;wq_{\text{2}}(\text{1}-\gamma )\leq k<wq_{\text{2}},(borrowing\;without\;bankruptcy\;risk)\\ & q_{\text{0}}(w,\;\beta ,k) & , & if\;\text{0}\leq k<wq_{\text{2}}(\text{1}-\gamma ),\;(borrowing\;with\;bankruptcy\;risk) \end{array}\right.\; \end{array} \end{array}$$ (3)

*where* $q_{\text{1}},\;q_{\text{2}},$ *and* $q_{\text{0}}$ *are defined as follows:* $q_{\text{1}}(w)={\overset{―}{F}}^{-\text{1}}\left(\frac{we^{r_{f}}-s}{p-s}\right)$*,* $q_{\text{2}}(w,R)={\overset{―}{F}}^{-\text{1}}\left(\frac{we^{R}-s}{p-s}\right)$*,* $q_{\text{0}}(w,\beta ,k)=\frac{k}{w(\text{1}-\beta )}$*.*

Let the cutoffs of the retailer’s working capital be $k_{i}(w)$, where i=1,2,3, as shown in S1 Appendix A (S1 File). According to Proposition 1, the retailer determines the optimal order quantity based on his working capital level. When the retailer is rich enough, i.e., $k\geq k_{\text{1}}(w)$, his optimal order quantity $q_{r}^{*}$ is the traditional newsvendor order quantity. When the retailer’s working capital lies in $\left[k_{\text{2}}(w),k_{\text{1}}(w\right)$], although the working capital cannot support the traditional newsvendor quantity, he is unwilling to assume debt. Thus, the retailer orders $q_{r}^{*}=\frac{k}{w}$ by only using all working capital. When the retailer’s working capital falls to $\left[k_{\text{3}}(w),k_{\text{2}}(w)\right)$, he borrows below the loan limit to place an order $q_{\text{2}}(w,R)$ without the bankruptcy risk. Finally, if the retailer’s working capital is quite low, i.e., $\text{0}\leq k<k_{\text{3}}(w)$, he can only place a *loan-limit order quantity* $q_{\text{0}}(w,\beta ,k)$ by borrowing up to the loan limit and may go bankrupt.

**Fig 2:**
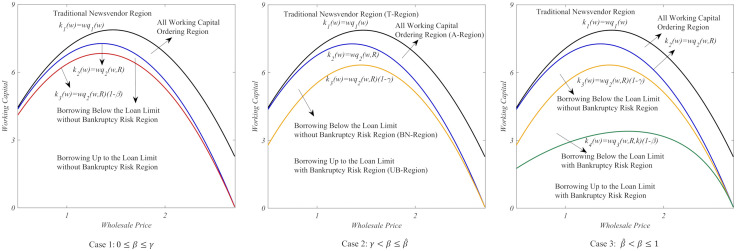
Retailer’s Response Regions Depend on Working Capital and Wholesale Price.

All above results of Lemma 3 and Proposition 1 depend on working capital and wholesale price, as shown in Fig 2.

A fairly low inventory advance rate, i.e., 0≤β≤γ (see Case 1 in Fig 2), implies that the product’s salvage value is adequate to cover loan obligations even under no demand, thereby eliminating the retailer’s bankruptcy risk. In this case, the retailer’s borrowing either reaches the loan limit in the *Borrowing Up to the Loan Limit without Bankruptcy Risk Region* or remains below it in the *Borrowing Below the Loan Limit without Bankruptcy Risk Region*. Conversely, if the inventory advance rate is excessively high, i.e., $\hat{\beta }<\beta \leq \text{1}$ (see Case 3 in Fig 2), the retailer faces bankruptcy risk whenever borrowing occurs, regardless of whether the loan reaches the limit. The retailer’s bankruptcy region then splits into two sub-regions: *Borrowing Below the Loan Limit with Bankruptcy Risk Region* and *Borrowing Up to the Loan Limit with Bankruptcy Risk Region.* For the retailer borrowing up to the loan limit, since the bankruptcy probability $F(\hat{\xi }(q_{\text{0}}))$ rises with β, the bank’s risk of not receiving full repayment increases when $\beta \in (\hat{\beta },\text{1}]$.

The bank’s profitability can be balanced against the retailer’s default risk by adjusting the inventory advance rate. The fact that regulatory frameworks such as Basel III require banks to maintain capital adequacy and manage credit risk exposures, making it unacceptable for them to face the higher possibility of incomplete loan repayment, as shown in Case 3 of Fig 2. In this case, the risk outweighs the bank’s potential earnings. Conversely, Case 1 of Fig 2 resembles the classic newsvendor problem, which is not concerned in this paper. Although the retailer faces no bankruptcy risk, the bank earns less interest income due to the small loan amount caused by a low inventory advance rate. Thus, we focus on Case 2 ($\gamma <\beta \leq \hat{\beta }$), where the retailer borrows with moderate bankruptcy risk. We refer to IBF under these bounded inventory advance rates as shown in Case 2 to IBF-B. This is consistent with the practice, where banks typically set inventory advance rates above a very low level but below an upper bound, such as around 50% [7,8] or 50%−65% [9]. In this situation, the retailer has only one bankruptcy region – borrowing up to the loan limit (see Case 2 in Fig 2) – and the optimal order quantity $q_{\text{0}}(w,\beta ,k)=\frac{k}{w(\text{1}-\beta )}$ depends solely on the inventory advance rate rather than the interest rate. Thus, the bank can only set the inventory advance rate within its bounds to manage the bankruptcy risk of the retailer whose borrowing reaches the loan limit.

For clarity, Fig 2 defines four regions. When $q_{r}^{*}=q_{\text{1}}(w)$, we refer to it as the *Traditional Newsvendor Region* (*T-Region*). While $q_{r}^{*}=\frac{k}{w}$, it is the *All Working Capital Ordering Region* (*A- Region*). The region where $q_{r}^{*}=q_{\text{2}}(w,R)$ is denoted as the *Borrowing Below the Loan Limit without Bankruptcy Risk Region* (*BN-Region*). The bank sets an inventory advance rate $\beta \in (\gamma ,\hat{\beta }]$ to determine the loan limit and mitigate its losses in the case of default. Finally, when the retailer is sufficient capital-constrained such that he must borrow up to the loan limit, incurring bankruptcy risk, this region is called as the *Borrowing Up to the Loan Limit with Bankruptcy Risk Region* (*UB-Region*).

### 4.3 Sensitivity analysis of retailer’s order quantity

#### 4.3.1 Impact of working capital.

**Proposition 2.** *For a demand distribution with IFR, in the UB-Region, both optimal order quantity* $q_{r}^{*}$ *and bankruptcy threshold* $\hat{\xi }\left(q_{r}^{*}\right)$ *of the retailer increase with the working capital* k *and there exists* $\frac{dq_{r}^{*}}{dk}=\frac{\text{1}}{w(\text{1}-\beta )}\geq \frac{\text{1}}{w}$*. In A-Region,* $q_{r}^{*}$ *increases with* k *and* $\frac{dq_{r}^{*}}{dk}=\frac{\text{1}}{w}$*. Finally, in the BN-Region and T-Region,* $q_{r}^{*}$ *does not depend on* k*. Furthermore, the optimal expected final cash flow* $E\left[v_{r}\left(q_{r}^{*}\right)\right]$ *increases with* k *in all these regions.*

**Fig 3:**
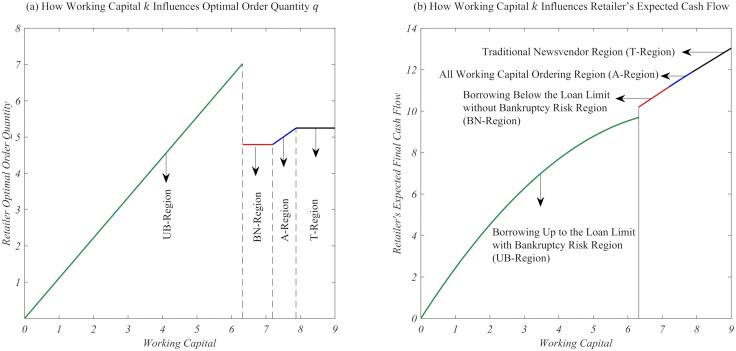
How Working Capital k Influences Optimal Order Quantity and Retailer’s Expected Cash Flow.

From Proposition 2, the retailer in the *UB-Region* is so poor that, with sufficient working capital, he would order more to increase profits. However, the larger the order, the higher the bankruptcy risk, which in turn exposes the bank to greater credit risk. The results of Proposition 2 are illustrated in Fig 3, which is described as the retailer’s response regions for p=3, w=1.5, c=0.5, s=0.2, R=0.1, β=0.6, $r_{f}=\text{0}.\text{0}\text{2}$ and the uniform demand distribution ξ~U(0,10). All parameter settings satisfy the assumptions.

As shown in Fig 3(a), the retailer’s optimal order quantity increases as working capital rises, when he orders using all working capital or borrowing up to the loan limit. Moreover, Fig 3(b) shows that the retailer’s optimal expected final cash flow nonlinearly increases with his working capital, and the rate of increase is decreasing in the *UB-Region*.

#### 4.3.2 Impact of interest rates and inventory advance rates.

**Lemma 4.** *For a demand distribution characterized by an increasing and convex failure rate, the retailer’s optimal order quantity in the BN-Region decreases as interest rate* R *increases. And the optimal order quantity in UB-Region increases with the inventory advance rate* β*.*

Note that the subsequent analysis in this paper is based on such a demand distribution with an increasing and convex failure rate. For example, power, truncated normal, uniform, exponential distribution, as well as Weibull distribution with shape parameter greater than one can be converted to standard normal distribution (see Zhou and Groenevelt [14]; Kouvelis and Zhao [32]).

For the retailer borrowing below the loan limit, the optimal order quantity declines as the interest rate rises, since higher financing costs raise the effective purchase cost. However, when the retailer borrows up to the loan limit, the optimal order quantity depends on inventory advance rates rather than interest rates. This highlights the importance of the impact of inventory advance rates. Intuitively, a higher advance rate allows the retailer to access a larger loan amount, thereby increasing his order quantity.

## 5. Wholesale price contract between retailer and supplier

### 5.1 Wholesale price corresponding to limited working capital

In this section, we examine the supplier’s wholesale price decision under a given loan contract. Given that suppliers can establish pricing tiers by segmenting customers based on order quantity, order frequency, or customer type [48], and according to Assumption 1 that all parameters are known to three parties, the supplier can tailor the wholesale price for retailers with different working capital levels, which serves as one basis for categorizing customer types. Moreover, the working capital cutoffs $k_{i}(w)$ presented in Section 4.2 depend on the wholesale price. It is necessary to investigate how these cutoffs, i.e., $k_{\text{1}}(w)$, $k_{\text{2}}(w)$, and $k_{\text{3}}(w)$, vary with wholesale price w.

**Lemma 5.** *For a demand distribution characterized by an increasing and convex failure rate,* $k_{\text{1}}(w)$*,* $k_{\text{2}}(w)$ *and* $k_{\text{3}}(w)$ *are all concave functions of* w*. Let the maximum value*$\;k_{\text{1}}(w{)}_{max}=\tilde{k}$*,* $k_{\text{2}}(w{)}_{max}=\overset{―}{k}$*, and* $k_{\text{3}}(w{)}_{max}=\underset{―}{k}$*. There exists* $\underset{―}{k}\leq \overset{―}{k}\leq \tilde{k}$*. For* $k\leq k_{i}(w{)}_{max}$ (i=1,2,3)*, if and only if* $s\leq min\left\{\frac{we^{r_{f}}}{\text{2}}+\frac{we^{r_{f}}z^{\prime }\left(q_{\text{1}}\right)}{\text{2}z\left(q_{\text{1}}\right)},\frac{we^{R}}{\text{2}}+\frac{we^{R}z^{\prime }\left(q_{\text{2}}\right)}{\text{2}z\left(q_{\text{2}}\right)}\right\}$*, there are at most two values for* w *so that the retailer considers using up all working capital to determine optimal order quantity, regardless of whether bank loans are required. That is,*

(1) *For* $\overset{―}{k}<k\leq \tilde{k}$*, there exist* $w_{t\text{1}}$ *and* $w_{t\text{2}}$*, i.e.,* $\text{0}\leq w_{t\text{1}}\leq w_{t\text{2}}\leq pe^{-r_{f}}$*, so that* $k_{\text{1}}(w)=k$;
(2) *For* $\underset{―}{k}<k\leq \overset{―}{k}$*, there exist* $w_{t\text{3}}$ *and* $w_{t\text{4}}$*, i.e.,* $w_{t\text{1}}\leq w_{t\text{3}}\leq w_{t\text{4}}\leq w_{t\text{2}}$*, so that* $k_{\text{2}}(w)=k$;
(3) *For* $\text{0}\leq k\leq \underset{―}{k}$*, there exist* $w_{t\text{5}}$ *and* $w_{t\text{6}}$*, i.e.,* $w_{t\text{3}}\leq w_{t\text{5}}\leq w_{t\text{6}}\leq w_{t\text{4}}$*, so that* $k_{\text{3}}(w)=k$*.*

**Fig 4:**
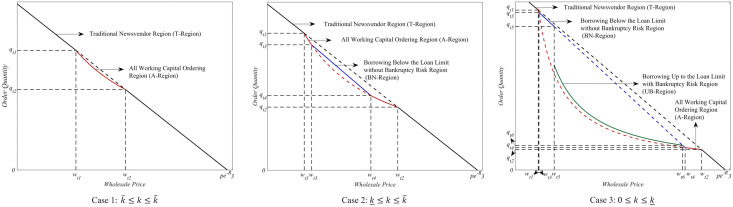
Retailer’s response to wholesale price w.

According to the results of Lemma 5, we describe the retailer’s response to w in Fig 4, where $q_{ti}$ is the order quantity corresponding to the cutoffs ($w_{ti}$) of wholesale price, i=1,2,3,4,5,6.

It is noted that, if the working capital is large enough, i.e., $k>\tilde{k}$, the retailer only needs to consider adopting traditional newsvendor model to determine order quantity. From Lemma 5 and Fig 4, When $\overset{―}{k}\leq k\leq \tilde{k}$ (see Case 1 in Fig 4), the retailer is in the *A-Region* with an order quantity $\frac{k}{w}$ if the supplier offers a medium wholesale price, i.e., $w\in [w_{t\text{1}},w_{t\text{2}}]$; otherwise, he follows the traditional newsvendor order quantity $q_{\text{1}}(w)$ if the wholesale price drifts toward either side, i.e., $w<w_{t\text{1}}$ or $w>w_{t\text{2}}$. When $\underset{―}{k}\leq k\leq \overset{―}{k}$ (see Case 2 in Fig 4), similar to Case 1, if the wholesale price is medium, i.e., $w{\in [w}_{t\text{3}},w_{t\text{4}}]$, the retailer lies in the *BN-Region,* with an optimal order quantity $q_{\text{2}}(w)$. Otherwise, the retailer may be in either the *T-Region* or the *A-Region* for the other ranges of the wholesale price. Finally, the bank loans offered to retailers with working capital $\text{0}\leq k\leq \underset{―}{k}$ (see Case 3 in Fig 4) are subject to the loan limits. Thus, if the supplier offers the medium wholesale price $w\in [w_{t\text{5}},w_{t\text{6}}]$, the retailer sits in the *UB-Region* and orders $q_{\text{0}}(w)$. For other wholesale price ranges, the retailer could be in one of the other three regions.

In summary, we can obtain the following Proposition 3 according to the above analysis.

**Proposition 3.** *For a demand distribution characterized by an increasing and convex failure rate, given* k*,* R *and* $\gamma <\beta \leq \hat{\beta }$*, the retailer’s optimal order quantity* $q_{r}^{*}$ *decreases as the wholesale price* w *increases. Furthermore, the relationship between* $q_{r}^{*}$ *and* w *is illustrated in* Table 3.

**Table 3:** Optimal order quantity corresponding to wholesale price.

The working capital k	The wholesale price w	The optimal order quantity $q_{r}^{*}$
$k\geq \tilde{k}$	$\text{0}\leq w\leq pe^{-R}$	$q_{r}^{*}=q_{\text{1}}={\overset{―}{F}}^{-\text{1}}\left(\frac{we^{r_{f}}-s}{p-s}\right)$
$\overset{―}{k}\leq k\leq \tilde{k}$	$\text{0}\leq w\leq w_{t\text{1}}$	
	$w_{t\text{2}}\leq w\leq pe^{-R}$	
	$w_{t\text{1}}\leq w\leq w_{t\text{2}}$	$q_{r}^{*}=\frac{k}{w}$
$\underset{―}{k}\leq k\leq \overset{―}{k}$	$w_{t\text{1}}\leq w\leq w_{t\text{3}}$	
	$w_{t\text{4}}\leq w\leq w_{t\text{2}}$	
	$w_{t\text{3}}\leq w\leq w_{t\text{4}}$	$q_{r}^{*}=q_{\text{2}}={\overset{―}{F}}^{-\text{1}}\left(\frac{we^{R}-s}{p-s}\right)$
$\text{0}\leq k\leq \underset{―}{k}$	$w_{t\text{3}}\leq w\leq w_{t\text{5}}$	
	$w_{t\text{6}}\leq w\leq w_{t\text{4}}$	
	$w_{t\text{5}}\leq w\leq w_{t\text{6}}$	$q_{r}^{*}=q_{\text{0}}=\frac{k}{(\text{1}-\beta )w}$

Proposition 3 states that with a specified interest rate R and a bounded inventory advance rate $\beta \in \left(\gamma ,\hat{\beta }\right]$, the retailer determines the optimal order quantity by considering wholesale price intervals set by the supplier. Intuitively, if the retailer is wealthy enough, i.e., $k\geq \tilde{k}$, he can order traditional newsvendor optimal order quantity $q_{\text{1}}$ no matter what the wholesale price is taken in $\left[\text{0},pe^{-R}\right]$. However, when the supplier offers a moderate wholesale price within $\left[w_{t\text{1}},w_{t\text{2}}\right]$, the retailer whose working capital is in $\left[\overset{―}{k},\tilde{k}\right]$ will order $\frac{k}{w}$; otherwise, he will order at the traditional newsvendor optimal order quantity $q_{\text{1}}(w).$ Similarly, the medium poor retailer, i.e., $\underset{―}{k}\leq k\leq \overset{―}{k}$, will order optimal quantity $q_{\text{2}}(w,R)$ at a wholesale price within $\left[w_{t\text{3}},w_{t\text{4}}\right]$; otherwise, he uses up all his working capital to order. Finally, for the really poor retailer, i.e., $\text{0}\leq k\leq \underset{―}{k}$, he borrows up to the loan limit to order if the wholesale price is moderate, i.e., $w_{t\text{5}}\leq w\leq w_{t\text{6}}$.

### 5.2 Equilibrium solutions for retailer and supplier

It is supposed that the capital-constrained retailer relies on IBF-B to finance his inventory investment for maximizing profits. In this situation, the supplier receives a payment of $wq_{r}^{*}(w)$ at the beginning of the selling season and production costs are $cq_{r}^{*}(w)$, where $q_{r}^{*}(w)$ is the retailer’s optimal order quantity for specified values of β and R. When a capital-constrained retailer signs an IBF-B loan contract with a bank, the bank directly credits these funds to the supplier. As a result, the supplier receives the full payment $wq_{r}^{*}(w)$ to cover production costs. Hence, she has no bankruptcy risk and the final profits are


$$\begin{array}{c} \begin{array}{c} \pi_{s}(w)=(w-c)q_{r}^{*}(w)e^{r_{f}}. \end{array} \end{array}$$ (4)

According to Proposition 3, when $k>\tilde{k}$, the results are equivalent to traditional newsvendor problem, which has been well studied [49]. Thus, our following work focuses on the remainder cases, that is, $\overset{―}{k}\leq k\leq \tilde{k}$, $\underset{―}{k}\leq k\leq \overset{―}{k}$ and $\text{0}\leq k\leq \underset{―}{k}$. For the sake of simplifying the expression, the retailer who orders using all working capital and does not borrow from the bank, i.e., $\overset{―}{k}\leq k\leq \tilde{k}$, is called an NB-retailer; the retailer who intends to borrow below the loan limit, i.e., $\underset{―}{k}\leq k\leq \overset{―}{k}$, is called a BB-retailer; and the retailer who intends to borrow up to the loan limit, i.e., $\text{0}\leq k\leq \underset{―}{k}$, is called a BU-retailer.

#### 5.2.1 Retailer who orders using all working capital (NB-retailer).

As a benchmark, we first examine the wholesale pricing for the NB-retailer who does not borrow and has no bankruptcy risk. We then focus on the financing relevant cases under IBF-B.

When $w_{t\text{1}}\leq w\leq w_{t\text{2}}$, the retailer exhausts all working capital to place orders. Otherwise, the retailer behaves as a traditional newsvendor, whose optimal order quantity $q_{s\text{1}}$ satisfies the following condition for $w\leq w_{t\text{1}}$ or $w\geq w_{t\text{2}}$ [49]:


$$\begin{array}{c} \begin{array}{c} \overset{―}{F}\left(q_{s\text{1}}\right)-q_{s\text{1}}f\left(q_{s\text{1}}\right)-\frac{ce^{r_{f}}-s}{p-s}=\text{0}. \end{array} \end{array}$$ (5)

The corresponding wholesale price is $w_{s\text{1}}=\left[(p-s)\overset{―}{F}\left(q_{s\text{1}}\right)+s\right]e^{-r_{f}}$. Proposition 4 summarizes the benchmark equilibrium outcomes for the supplier and NB-retailers.

**Proposition 4.** *For* $\overset{―}{k}\leq k\leq \tilde{k}$*, the global equilibrium solutions are* $\left(w^{*},q^{*}\right)=(w_{s\text{1}},q_{s\text{1}})$ *if* $w_{s\text{1}}\geq w_{t\text{2}}$*, and alternatively,* $\left(w^{*},q^{*}\right)=(w_{t\text{2}},q_{t\text{2}})$ *if* $w_{s\text{1}}<w_{t\text{2}}$*.*

The benchmark results indicate that when the NB-retailer’s working capital is insufficient to attain the traditional newsvendor order quantity, the supplier adjusts the wholesale price to induce the retailer to exhaust all working capital, corresponding to the equilibrium $\left(w^{*},q^{*}\right)=(w_{t\text{2}},q_{t\text{2}})$.

#### 5.2.2 Retailer who intends to borrow below loan limit (BB-retailer).

The BB-retailer borrows from the bank without bankruptcy for $w_{t\text{3}}\leq w\leq w_{t\text{4}}$. Now, the supplier’s problem is formulated as


$$\begin{array}{c} \pi_{s}\left(q_{r}^{*}\right)=\underset{q_{r}^{*}(w)>\text{0}}{\text{max}}(w-c)q_{r}^{*}e^{r_{f}},\\ \begin{array}{c} s.t.\;(p-s)\overset{―}{F}\left(q_{r}^{*}\right)-\left(we^{R}-s\right)=\text{0}, \end{array} \end{array}$$ (6)

where Equation (6) represents the first-order condition for determining the retailer’s optimal order quantity at a specified wholesale price. The supplier establishes a wholesale price to optimize her final profits for the BB-retailer. This result is formalized in Proposition 5.

**Proposition 5.** *Given an interest rate* R *and a bounded inventory advance rate* $\beta \in \left(\gamma ,\hat{\beta }\right]$, *the global equilibrium solutions* $\left(w^{*},q^{*}\right)$ *for* $\underset{―}{k}\leq k\leq \overset{―}{k}$ *are*

(1) $\left(w^{*},q^{*}\right)=\left(w_{s\text{1}},q_{s\text{1}}\right)$*, if* $w_{s\text{1}}\geq w_{t\text{2}}$ *and* $w_{s\text{2}}>w_{t\text{4}}$;
(2) $\left(w^{*},q^{*}\right)=argmax\pi_{s}(w,q)$*, where* $\left\{(w,q)|\left(w_{s\text{1}},q_{s\text{1}}\right),\left(w_{s\text{2}},q_{s\text{2}}\right)\right\}$*, if* $w_{s\text{1}}\geq w_{t\text{2}}$ *and* $w_{s\text{2}}\leq w_{t\text{4}}$;
(3) $\left(w^{*},q^{*}\right)=(w_{t\text{2}},q_{t\text{2}})$*, if* $w_{s\text{1}}<w_{t\text{2}}$ *and*$\;w_{s\text{2}}>w_{t\text{4}}$;
(4) $\left(w^{*},q^{*}\right)=argmax\pi_{s}(w,q)\;$*, where* $\left\{(w,q)|(w_{t\text{2}},q_{t\text{2}}),\left(w_{s\text{2}},q_{s\text{2}}\right)\right\}$*, if* $w_{s\text{1}}<w_{t\text{2}}$ *and* $w_{s\text{2}}\leq w_{t\text{4}}$*.*

It is worth mentioning that the equilibrium solutions $\left(w_{s\text{1}},q_{s\text{1}}\right)$ and $\left(w_{t\text{2}},q_{t\text{2}}\right)$ are not considered simultaneously, due to the fact that $w_{t\text{2}}$ is a threshold for $w_{s\text{1}}$. Once $w_{s\text{1}}$ is less than $w_{t\text{2}}$, the retailer orders with all working capital and hence the equilibrium solution becomes $\left(w_{t\text{2}},q_{t\text{2}}\right)$. That is, if the wholesale price $w_{s\text{1}}$ lies within the *T-Region*, the supplier will charge $w_{s\text{1}}$ as the candidate optimal wholesale price; otherwise, $w_{t\text{2}}$ will be the candidate optimal wholesale price. Then, if supplier realizes that the equilibrium solution $\left(w_{s\text{2}},q_{s\text{2}}\right)$ is in the *BN-Region*, i.e., $w_{s\text{2}}\leq w_{t\text{4}}$, she will charge the optimal wholesale price that makes her more profitable out of $w_{s\text{1}}$ (or $w_{t\text{2}}$) and $w_{s\text{2}}$; otherwise, the optimal wholesale price is either $w_{s\text{1}}$ or $w_{t\text{2}}$ as we discussed above.

#### 5.2.3 Retailer who intends to borrow up to loan limit (BU-retailer).

Finally, the retailer can only adopt the *loan-limit order quantity* when the wholesale price $w\in [w_{t\text{5}},w_{t\text{6}}]$. Then the supplier’s problem shows as follows.


$$\begin{array}{c} \pi_{s}\left(q_{r}^{*}\right)=\underset{q_{r}^{*}(w)>\text{0}}{\text{max}}(w-c)q_{r}^{*}e^{r_{f}},\\ \begin{array}{c} s.t.\;wq_{r}^{*}-k-\beta wq_{r}^{*}=\text{0}, \end{array} \end{array}$$ (7)

where Equation (7) is satisfied by *loan-limit order quantity* $q_{\text{0}}(w)$. Proposition 6 shows how the supplier offers the wholesale price contract to the BU-retailer.

**Proposition 6.** *Given an interest rate* R *and a bounded inventory advance rate* $\beta \in \left(\gamma ,\hat{\beta }\right]$, *the global equilibrium solutions* $\left(w^{*},q^{*}\right)$ *for* $\text{0}\leq k\leq \underset{―}{k}$ *are*

(1) $\left(w^{*},q^{*}\right)=argmax\pi_{s}(w,q)$*, where* $\left\{(w,q)|\left(w_{s\text{1}},q_{s\text{1}}\right),\left(w_{t\text{6}},q_{\text{0}}\left(w_{t\text{6}}\right)\right)\right\}$*, if* $w_{s\text{1}}\geq w_{t\text{2}}$
(2) $\left(w^{*},q^{*}\right)=argmax\pi_{s}(w,q)$*, where* $\left\{(w,q)|\left(w_{t\text{2}},q_{t\text{2}}\right),\left(w_{t\text{6}},q_{\text{0}}\left(w_{t\text{6}}\right)\right)\right\}$*, if* $w_{s\text{1}}<w_{t\text{2}}$*.*

The supplier can charge the highest feasible wholesale price $w_{t\text{6}}$ to enable the retailer with $\text{0}\leq k\leq \underset{―}{k}$ to borrow up to the loan limit to order $q_{\text{0}}\left(w_{t\text{6}}\right)$. Similar to the analysis in Section 5.2.2, the supplier finally chooses the profitable wholesale price contract, i.e., $\left(w_{t\text{6}},q_{\text{0}}\left(w_{t\text{6}}\right)\right)$ and $\left(w_{s\text{1}},q_{s\text{1}}\right)$ (or $\left(w_{t\text{2}},q_{t\text{2}}\right)$). In other words, the supplier may refrain from offering the wholesale price contract $\left(w_{t\text{6}},q_{\text{0}}\left(w_{t\text{6}}\right)\right)$, because such a contract yields lower profit than the alternatives.

## 6. Loan menu offered by bank towards retailer

In this section, we investigate the bank’s loan contract decisions under a given wholesale price. The bank provides a loan menu comprising an interest rate and an inventory advance rate constrained within $\left(\gamma ,\hat{\beta }\right]$. In addition, the bank incurs a financing cost rate $r_{c}$ associated with inventory pledge services of a third-party logistics company. Also, consistent with Assumption 1, all parameters are assumed to be observable to the three parties. The bank aims to maximize its expected profit by jointly determining the interest rate and inventory advance rate under IBF-B.

### 6.1 Bank’s expected final profits

Let $\pi_{b}(R,\beta )$ represent the bank’s final profits. The retailer can fully repay bank loans $l(q)e^{R}$ if the realized demand exceeds the *bankruptcy threshold*. Otherwise, in the event of default, the repayments to the bank are the sales revenue $pmin\left(\xi ,q_{r}^{*}\right)$ plus the salvage value of unsold products $s{\left(q_{r}^{*}-\xi \right)}^{+}$. Then, the bank’s final profits are


$$\begin{array}{c} \pi_{b}(R,\beta )=min\left\{l(q)e^{R},pmin\left(\xi ,q_{r}^{*}\right)+s{\left(q_{r}^{*}-\xi \right)}^{+}\right\}-l(q)e^{r_{c}}. \end{array}$$

From Proposition 1, we can derive that the expected final profits for the bank are


$$\begin{array}{c} E\left[\pi_{b}(R,\beta )\right]=\left\{ \begin{array}{cccc} & \text{0} & , & if\;k\geq wq_{\text{2}}\;(a)\\ & \left(wq_{\text{2}}(w,R)-k\right)\left(e^{R}-e^{r_{c}}\right) & , & if\;wq_{\text{2}}(\text{1}-\gamma )\leq k<wq_{\text{2}}\;(b)\\ & \beta wq_{\text{0}}(w,\beta ,k)\left(e^{R}-e^{r_{c}\;}\right)\\ & -(p-s)\left(\hat{\xi }\left(q_{\text{0}}(w,\beta ,k)\right)-\int_{\text{0}}^{\hat{\xi }\left(q_{\text{0}}(w,\beta ,k)\right)}\overset{―}{F}(\xi )d\xi \right) & . & if\;\text{0}\leq k<wq_{\text{2}}(\text{1}-\gamma )\;(c) \end{array}\right. \end{array}$$ (8)

Therein, Equation (8a) indicates that the retailer has no demand for IBF-B service when working capital is adequate to support the optimal order quantity, i.e., $k\geq wq_{\text{2}}$; consequently, the bank earns no profit from providing the IBF-B service.

In practice, banks commonly offer IBF-B based on the given inventory value that depends on wholesale prices. Accordingly, for a given wholesale price, we first examine how the bank jointly determines an interest rate and an inventory advance rate to optimize its expected final profits. Furthermore, in Section 7.1, we extend to illustrate the interaction between the wholesale price contract and the loan menu under a given demand distribution, namely, the uniform distribution.

### 6.2 How bank determines loan menu

As we mentioned above, the retailer’s operations are constrained by loan terms. Based on the results in Section 5, we first examine how interest rates affect the retailer’s working capital cutoffs which are independent of inventory advance rates. Following this, we will explore the interplay between interest rates and the retailer’s optimal order quantity. Initially, we reformulate working capital cutoffs $k_{i}(w)$ as $k_{i}(R)$, i=2,3. Then, we conclude the following lemma.

**Lemma 6.** *Both* $k_{\text{2}}(R)\;$and $k_{\text{3}}(R)\;$*decrease with* R. *Thus,* $k_{i}(R{)}_{max}=k_{i}\left(r_{c}\right)$ *and* $k_{\text{3}}(R{)}_{max}\leq k_{\text{2}}(R{)}_{max}$*. For* $k\leq k_{i}(R{)}_{max}$ (i=2,3)*, there exists a value for* R *so that the retailer exhausts all working capital to determine optimal order quantity, regardless of whether borrowing is necessary. That is*

(1) *For* $k_{\text{3}}\left(r_{c}\right)<k\leq k_{\text{2}}\left(r_{c}\right)$*, there exists a value of* R*, i.e.,* $r_{c}\leq R_{t\text{1}}\leq ln\left(\frac{p}{w}\right)$*, so that* $k_{\text{2}}(R)=k$;
(2) *For* $\text{0}\leq k\leq k_{\text{3}}\left(r_{c}\right)$*, there exists a value of* R*, i.e.,* $r_{c}\leq R_{t\text{2}}{\leq R}_{t\text{1}}$*, so that* $k_{\text{3}}(R)=k$*.*

**Fig 5:**
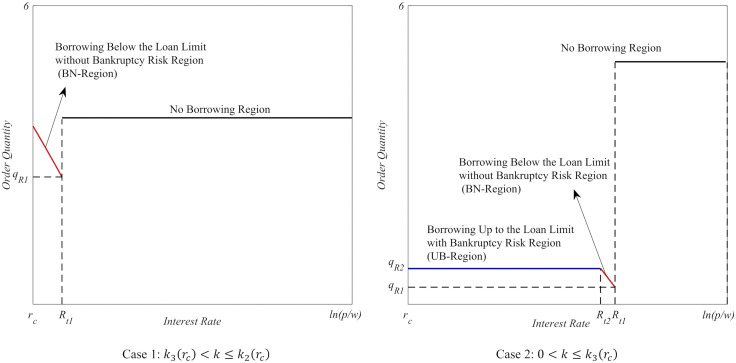
The relationship between retailer’s q and bank’s R.

According to the results in Lemma 6, Fig 5 illustrates the correlation between interest rates and the retailer’s order quantity. Note that let $q_{Ri}$ be the corresponding order quantity for $R_{ti}$, where i=1 and 2.

As discussed above, the retailer with working capital $k>k_{\text{2}}\left(r_{c}\right)$ can place the optimal order quantity $q_{r}^{*}=min\left(q_{\text{1}},\frac{k}{w}\right)$ without borrowing. From Lemma 6 and Fig 5, if the interest rate R charged by the bank lies in $\left[r_{c},\;R_{t\text{1}}\right]$, the retailer with $k_{\text{3}}\left(r_{c}\right)<k\leq k_{\text{2}}\left(r_{c}\right)$ (see Case 1 in Fig 5) places an order $q_{\text{2}}(w,R)$ by borrowing below the loan limit, which falls into the *BN-Region*. However, if the bank charges a higher interest rate, i.e., $R\in [R_{t\text{1}},\;ln(\frac{p}{w})]$, the retailer will not adopt IBF-B due to the high financing cost. The upper bound $ln(\frac{p}{w\;})$ follows from the constraint that the purchase cost cannot exceed the selling price, i.e., $we^{R}\leq p$. Then, for the retailer with $\text{0}\leq k\leq k_{\text{3}}\left(r_{c}\right)$ (see Case 2 in Fig 5), although the *loan-limit order quantity* $q_{\text{0}}(w,\beta ,k)$ is independent of interest rates, the bank can still optimize its expected final profits by charging an optimal interest rate $R^{*}\in \left[r_{c},R_{t\text{2}}\right]$, which lies in the *UB-Region*.

As we mentioned above, the inventory advance rate serves as another loan term used to manage the bank’s credit risk in IBF-B. It is worth noting that the bank’s core purpose of presetting inventory advance rate bounds is to control credit risk. As explained in Section 4.2, the retailer has higher bankruptcy risk if the inventory advance rate falls within a higher range, i.e., $\hat{\beta }<\beta \leq \text{1}$, which is not what the bank do expect. Therefore, the bank considers offering an appropriate inventory advance rate constrained within a medium range, i.e., $\left(\gamma ,\hat{\beta }\right]$, which not only mitigates the retailer’s bankruptcy risk, but also allows for credit profitability.

Now, considering that the bank can predict the retailer’s optimal order quantity and set an inventory advance rate within the medium range, loan menus that optimize the bank’s expected final profits are presented in Proposition 7.

**Proposition 7.** *For a demand distribution characterized by an increasing and convex failure rate, given* w*, and provided that the bank loan cost rate* $r_{c}$ *satisfies* $\overset{―}{\beta }\left(R_{t\text{2}}\right)>\gamma \left(r_{c}\right)$*, the optimal loan menu* $\left(R^{*},\beta^{*}\right)$ *is shown as follows.*

(1) *For* $k_{\text{3}}\left(r_{c}\right)<k\leq k_{\text{2}}\left(r_{c}\right)$*, if* $R_{bu}>r_{c}$*,*$R^{*}=R_{bu}$*, otherwise,* $R^{*}=r_{c}$*. And the bank can offer any inventory advance rate* $\beta^{*}$ *within the range* $\left(\gamma \left(r_{c}\right),\text{1}\right]$*.*
(2) *For* $\text{0}<k\leq k_{\text{3}}\left(r_{c}\right)$*, if* $\lambda_{\text{1}}\left(R_{t\text{2}},\hat{\beta }\left(r_{c}\right)\right)>\text{0}$*, the bank’s optimal loan menu is* $\left(R_{t\text{2}},\hat{\beta }\left(r_{c}\right)\right)$*. Otherwise, the bank offers* $\left(R_{t\text{2}},\overset{―}{\beta }\left(R_{t\text{2}}\right)\right)$ *as optimal loan menu.*

According to Proposition 7 and Lemma 6, because the BB-retailer with $k_{\text{3}}\left(r_{c}\right)<k\leq k_{\text{2}}\left(r_{c}\right)$ borrows below the loan limit and does not go bankrupt, the bank can set any inventory advance rate $\beta^{*}$ within the range $\left(\gamma \left(r_{c}\right),\text{1}\right]$*.* To obtain optimal expected final profits, the bank offers a medium interest rate $R_{bu}$ if $R_{bu}>r_{c}$; otherwise, it offers the lower bound $r_{c}$, implying the bank just breaks even in this business. The BB-retailer therefore faces a mild financing cost. By contrast, the BU-retailer with substantially lower working capital demands higher loan amounts and faces bankruptcy risk. Consequently, the bank always sets the highest feasible interest rate $R_{t\text{2}}$ to manage the credit risk, regardless of the inventory advance rate. According to part (2) in Proposition 7, the bank offers the highest feasible inventory advance rate, i.e., $\hat{\beta }\left(r_{c}\right)$, if the shadow price of the inventory advance rate is positive, i.e., $\lambda_{\text{1}}\left(R_{t\text{2}},\hat{\beta }\left(r_{c}\right)\right)>\text{0}$, implying that an increase in inventory advance rate yields a positive marginal contribution to the bank’s profit. Otherwise, the bank offers the interior inventory advance rate, i.e., $\overset{―}{\beta }\left(R_{t\text{2}}\right)$.

## 7. Extended discussions

Under IBF-B, the loan menu items, especially inventory advance rates, depend on the product’s wholesale price offered by the supplier. Conversely, the wholesale price and order quantity are affected by inventory advance rates and interest rates. However, due to the complexity of the model, we further derive the equilibrium decisions of participants under a special demand distribution, i.e., the uniform distribution, to investigate the interplay between the loan menu and wholesale price contract. Finally, we illustrate the impact of the IBF-B on the supply chain performance and the effects of key economic parameters on financing and operational decisions by a numerical analysis.

### 7.1 Equilibrium decisions of participants under uniform demand distribution

Assume that the demand ξ follows a uniform distribution with ξ~U(a,b). According to Section 5.2, if the wholesale price contract offered by the supplier is $\left(w_{s\text{2}},q_{s\text{2}}\right)$ or $\left(w_{t\text{6}},q_{\text{0}}\left(w_{t\text{6}}\right)\right)$, the BB-retailer with working capital $\underset{―}{k}(R)\leq k\leq \overset{―}{k}(R)$ will borrow below the loan limit or the BU-retailer with $\text{0}\leq k\leq \underset{―}{k}(R)$ will borrow up to the loan limit.

Similar to the analysis in Section 6.2, we obtain the results about the relationship between the working capital cutoffs ($\underset{―}{k}(R)$ and $\overset{―}{k}(R)$) and the interest rate R under the uniform demand distribution (see Lemma EC.1 in S1 Appendix B (S1 File)). Specifically, both working capital cutoffs ($\underset{―}{k}(R)$ and $\overset{―}{k}(R)$) increase with respect to R. Then, BB-retailers’ working capital lies in $\underset{―}{k}\left(r_{c}\right)<k\leq \overset{―}{k}\left(r_{c}\right)$, whereas BU-retailers’ working capital satisfies $\text{0}\leq k\leq \underset{―}{k}\left(r_{c}\right)$. For BB-retailers, the interest rate should lie within $\left[r_{c},R_{t\text{1}}^{U}\right]$ to induce them to consider IBF-B, whereas for BU-retailers, the corresponding range is $\left[r_{c},R_{t\text{2}}^{U}\right]$. These results characterize the interest rate ranges in which different retailers are willing to consider IBF-B and provide the basis for deriving Proposition 8. The equilibrium decisions of participants on the loan menu and wholesale price contract are then shown in Proposition 8 (see details in S1 Appendix B (S1 File)).

**Proposition 8.** *For a uniform demand distribution* ξ~U(a,b)*, and provided that the bank loan cost rate* $r_{c}$ *satisfies* $\frac{\text{2}(b-a)\sigma_{\text{5}}\left(r_{c}\right)b(p-s)}{\left[\sigma_{\text{4}}+\text{2}(b-a)\sigma_{\text{5}}\left(r_{c}\right)\right]\sigma_{\text{4}}}>\text{0}$, *the optimal loan menu* $\left({R^{U}}^{*},{\beta^{U}}^{*}\right)$ *and the optimal wholesale price contract* $\left({w^{U}}^{*},{q^{U}}^{*}\right)$ *are shown in* Tables 4 and 5, *respectively.*

**Table 4:** Optimal loan menu $\left({R^{U}}^{*},{\beta^{U}}^{*}\right)$.

The types of the retailers	The conditions of optimal loan menu	The optimal loan menu $\left({R^{U}}^{*},{\beta^{U}}^{*}\right)$
BB-retailer with $\underset{―}{k}\left(r_{c}\right)<k\leq \overset{―}{k}\left(r_{c}\right)$	$R_{bu}^{U}>r_{c}$	${(R}_{bu}^{U},\;\beta^{U*}),$ where $\beta^{U*}\in \left(\gamma \left(r_{c}\right),\text{1}\right]$
	$R_{bu}^{U}\leq r_{c}$	$(r_{c},\;\beta^{U*}),$ where $\beta^{U*}\in \left(\gamma \left(r_{c}\right),\text{1}\right]$
BU-retailer with $\text{0}<k\leq \underset{―}{k}\left(r_{c}\right)$	$\lambda_{\text{1}}^{\prime }\left(R_{t\text{2}}^{U},\hat{\beta }\left(r_{c}\right)\right)>\text{0}$	$\left(R_{t\text{2}}^{U},\hat{\beta }\left(r_{c}\right)\right)$
	$\lambda_{\text{1}}^{\prime }\left(R_{t\text{2}}^{U},\hat{\beta }\left(r_{c}\right)\right)\leq \text{0}$	$\left(R_{t\text{2}}^{U},\overset{―}{\beta }\left(R_{t\text{2}}^{U}\right)\right)$

**Table 5:** Optimal wholesale price contract $\left({w^{U}}^{*},{q^{U}}^{*}\right)$ and retailer’s credit decision.

The types of the retailers	The conditions of optimal wholesale price		The optimal wholesale price $\left({w^{U}}^{*},{q^{U}}^{*}\right)$	Whether the retailer uses IBF-B
BB-retailer with $\underset{―}{k}\left(r_{c}\right)<k\leq \overset{―}{k}\left(r_{c}\right)$	(a) $w_{s\text{1}}\geq w_{t\text{2}}$ and $w_{s\text{2}}\left({R^{U}}^{*}\right)>w_{t\text{4}}\left({R^{U}}^{*}\right)$		$\left(w_{s\text{1}},q_{s\text{1}}\right)$	Not Use
	$w_{s\text{1}}\geq w_{t\text{2}}$ and $w_{s\text{2}}\left({R^{U}}^{*}\right)\leq w_{t\text{4}}\left({R^{U}}^{*}\right)$	(b)$\;E\left[\pi_{s}\left(w_{s\text{1}}\right)\right]\geq E[\pi_{s}\left(w_{s\text{2}}\left({R^{U}}^{*}\right)\right)]$		
		(c)$\;E\left[\pi_{s}\left(w_{s\text{1}}\right)\right]<E[\pi_{s}\left(w_{s\text{2}}\left({R^{U}}^{*}\right)\right)]$	$\left(w_{s\text{2}}\left({R^{U}}^{*}\right),q_{s\text{2}}\left({R^{U}}^{*}\right)\right)$, where ${R^{U}}^{*}=max\left\{R_{bu}^{U},\;r_{c}\right\}$	Use
	(d) $w_{s\text{1}}<w_{t\text{2}}$ and $w_{s\text{2}}\left({R^{U}}^{*}\right)>w_{t\text{4}}\left({R^{U}}^{*}\right)$		$\left(w_{t\text{2}},q_{t\text{2}}\right)$	Not Use
	$w_{s\text{1}}<w_{t\text{2}}$ and $w_{s\text{2}}\left({R^{U}}^{*}\right)\leq w_{t\text{4}}\left({R^{U}}^{*}\right)$	(e)$\;E\left[\pi_{s}\left(w_{t\text{2}}\right)\right]\geq E[\pi_{s}\left(w_{s\text{2}}\left({R^{U}}^{*}\right)\right)]$		
		(f)$\;E\left[\pi_{s}\left(w_{s\text{1}}\right)\right]<E[\pi_{s}\left(w_{s\text{2}}\left({R^{U}}^{*}\right)\right)]$	$\left(w_{s\text{2}}\left({R^{U}}^{*}\right),q_{s\text{2}}\left({R^{U}}^{*}\right)\right)$	Use
BU-retailer with $\text{0}<k\leq \underset{―}{k}\left(r_{c}\right)$	$w_{s\text{1}}\geq w_{t\text{2}}$	(1) $E\left[\pi_{s}\left(w_{s\text{1}}\right)\right]\geq E[\pi_{s}\left({w_{t\text{6}}(R^{U}}^{*})\right)]$	$\left(w_{s\text{1}},q_{s\text{1}}\right)$	Not use
		(2) $E\left[\pi_{s}\left(w_{s\text{1}}\right)\right]<E[\pi_{s}\left({w_{t\text{6}}(R^{U}}^{*})\right)$	$\left(w_{t\text{6}}\left({R^{U}}^{*}\right),q_{\text{0}}\left(w_{t\text{6}}\left({R^{U}}^{*}\right),{\beta^{U}}^{*}\right)\right),$ where ${R^{U}}^{*}=R_{t\text{2}}^{U}$ and $\beta^{U^{*}}=min\left\{\overset{―}{\beta }\left(R_{t\text{2}}^{U}\right),\hat{\beta }\left(r_{c}\right)\right\}$	Use
	$w_{s\text{1}}<w_{t\text{2}}$	(3) $E\left[\pi_{s}\left(w_{t\text{2}}\right)\right]<E[\pi_{s}\left({w_{t\text{6}}(R^{U}}^{*})\right)$		
		(4) $E\left[\pi_{s}\left(w_{t\text{2}}\right)\right]\geq E[\pi_{s}\left({w_{t\text{6}}(R^{U}}^{*})\right)$	$\left(w_{t\text{2}},q_{t\text{2}}\right)$	Not use

As shown in Proposition 8, the structure of the bank’s optimal loan menu is similar to that in Proposition 7 where the wholesale price is fixed (exogenous). These results indicate that although the endogenous wholesale price affects the items in the loan menu, it leaves the fundamental nature of the bank’s decision-making unchanged. Specifically, for the BB-retailer who borrows below the loan limit and faces no bankruptcy risk, the bank offers a medium interest rate $R_{bu}^{U}$ if $R_{bu}^{U}>r_{c}$; otherwise, the bank just breaks even by charging $r_{c}$. But for the BU-retailer who borrows up to the loan limit and faces bankruptcy risk, the bank consistently charges the highest feasible interest rate $R_{t\text{2}}^{U}$. Meanwhile, the choice of the inventory advance rate depends on whether it yields positive marginal contribution to the bank’s profit. Consistent with the analysis in Section 5.2, the supplier examines whether each candidate equilibrium wholesale price lies within its feasible range and then chooses the profitable wholesale price contract.

As discussed above, both the supplier and the bank tailor the wholesale price contract and loan menu, respectively, according to the retailer’s working capital. Thus, we present how the retailer’s working capital impacts the decision-making of three parties for the BB-retailer with $\underset{―}{k}\left(r_{c}\right)<k\leq \overset{―}{k}\left(r_{c}\right)$ and the BU-retailer with $\text{0}<k\leq \underset{―}{k}\left(r_{c}\right)$ in Proposition 9.

**Proposition 9.** *(1) The interest rates* $R_{bu}^{U}$*,* $R_{t\text{2}}^{U}$*, the inventory advance rate* $\overset{―}{\beta }\left(R_{t\text{2}}^{U}\right)$ *and its upper bound* $\hat{\beta }\left(r_{c}\right)$ *decrease with respect to the working capital* k*.*

(2) *The wholesale prices* $w_{s\text{2}}\left(R_{bu}^{U}\right)$ *and* $w_{t\text{6}}\left(R_{t\text{2}}^{U}\right)$ *increase with respect to* k*. Conversely,* $w_{t\text{2}}$ *decreases with respect to* k*. Then,* $w_{s\text{1}}$ *and* $w_{s\text{2}}\left(r_{c}\right)$ *are independent of* k.
(3) *The order quantities* $q_{s\text{2}}\left(R_{bu}^{U}\right)$ *and* $q_{t\text{2}}$ *increase with respect to* k*. Conversely,* $q_{\text{0}}\left(w_{t\text{6}}\left(R_{t\text{2}}^{U}\right),\beta^{U*}\right)$ *decreases with respect to* k*. Then,* $q_{s\text{1}}$ *and* $q_{s\text{2}}\left(r_{c}\right)$ *are independent of* k.
(4) *The bankruptcy probability of the retailer who borrows up to the loan limit decreases with respect to* k*.*

**Fig 6:**
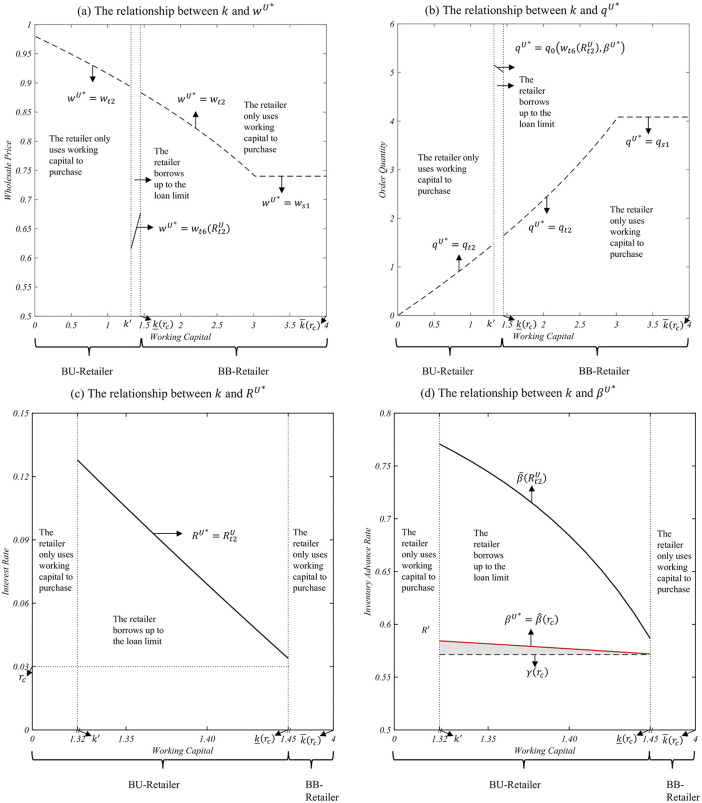
The impact of working capital on three parties’ equilibrium decisions.

We next present the following Fig 6 to provide insights into how the three parties make equilibrium decisions and how the retailer’s working capital affects these decisions. The parameters are p=1, c=0.5, s=0.4, $r_{f}=\text{0}.\text{0}\text{2}$ and the demand ξ follows a uniform distribution with ξ~U(0,10). Note that the equilibrium decision results of three parties are summarized in S1 Appendix B Table B3 (S1 File).

As illustrated in Figs 6(a) and 6(b), the result shows that the supplier offers a wholesale price to the poorer BU-retailers with $\text{0}<k<k^{\prime }$, allowing them to place orders only by using working capital. This occurs because the supplier earns higher profits when offering the wholesale price contract $\left(w_{t\text{2}},q_{t\text{2}}\right)$ to the poorer BU-retailers (see S1 Appendix B, Table B3 (S1 File)), indicating that the condition (4) in Table 5 is satisfied. By contrast, for richer BU-retailers with $k^{\prime }<k<\underset{―}{k}\left(r_{c}\right)$, the wholesale price contract $\left(w_{t\text{6}}\left(R_{t\text{2}}^{U}\right),q_{\text{0}}\left(w_{t\text{6}}\left(R_{t\text{2}}^{U}\right),{\beta^{U}}^{*}\right)\right)$ yields higher profits for the supplier and induces these retailers to borrow up to the loan limit, corresponding to condition (3) in Table 5. Therefore, the supplier offers $\left(w_{t\text{2}},q_{t\text{2}}\right)$ to poorer BU-retailers and $\left(w_{t\text{6}}\left(R_{t\text{2}}^{U}\right),q_{\text{0}}\left(w_{t\text{6}}\left(R_{t\text{2}}^{U}\right),{\beta^{U}}^{*}\right)\right)$ to richer BU-retailers.

Noteworthy, only BU-retailers with $k^{\prime }<k<\underset{―}{k}\left(r_{c}\right)$ accept the bank’s IBF-B contract, but this interval is relatively small, this region in Figs 6(c) and 6(d) is enlarged to more clearly illustrate the effect of retailer’s working capital on the interest rate and inventory advance rate.

Fig 6(d) shows that for richer BU-retailers, the bank’s optimal inventory advance rate should be set at the upper bound $\hat{\beta }\left(r_{c}\right)$. This finding suggests that the bank can effectively manage the credit risk by controlling the bounds of inventory advance rates. Additionally, as these BU-retailers become wealthier, the required loan amount decreases, and the bank can set a lower inventory advance rate. Fig 6(c) further shows that the bank charges lower interest rates to richer BU-retailers with less bankruptcy risk. Finally, the richest BU-retailer uses IBF-B at the lowest feasible inventory advance rate $\gamma \left(r_{c}\right)$.

Notably, under the above parameter settings, no BB-retailers receive wholesale price contracts that enable them to use IBF-B. This case corresponds to condition (e) in Table 5. Accordingly, the supplier offers $\left(w_{t\text{2}},q_{t\text{2}}\right)$ to most BB-retailers. However, for extremely rich BB-retailers, only the wholesale price $w_{s\text{1}}$ lies within its feasible range, i.e., $w_{s\text{1}}\geq w_{t\text{2}}$ (see S1 Appendix B, Table B3 (S1 File)). Consequently, the supplier offers the contract $\left(w_{s\text{1}},q_{s\text{1}}\right)$, corresponding to condition (a) in Table 5. In addition, Proposition 9 and Fig 6 show that as the working capital of BB-retailers (or poorer BU-retailers) increases, the supplier offers a lower wholesale price to induce larger order quantities, achieving higher profit.

### 7.2 Supply chain performance and IBF-B’s value

Since our study takes into account the interplay between the retailer-supplier wholesale price contract and the IBF-B loan menu, it is necessary to understand what the IBF-B can bring to the supply chain. Consequently, we evaluate the supply chain performance and the value of IBF-B by numerical analysis.

The integrated supply chain profit without capital constraints is represented by $\Pi_{cn}(q)=pmin(\xi ,q)+s(q-\xi {)}^{+}-cqe^{r_{f}}$. Lariviere and Porteus [49] have demonstrated that the expectation of $\Pi_{cn}(q)$ is concave with q and the optimal supply chain order quantity is $q_{cn}={\overset{―}{F}}^{-\text{1}}\left(\frac{ce^{r_{f}}-s}{p-s}\right)$. Let $E\left[\Pi_{cn}^{*}\right]=E\left[\Pi_{cn}\left(q_{cn}^{*}\right)\right]$. Then, $E\left[\Pi_{dn}^{*}\right]$ and $E\left[\Pi^{*}\right]=\sum_{i=r,s,b}E[\pi_{i}^{*}]$ are represented as the expected profits of the supply chain with capital constraints using only working capital and inventory-based financing with bounded advance rate (IBF-B), respectively, where $E\left[\pi_{i}^{*}\right]=E[\pi_{i}\left({q^{U}}^{*},\;{w^{U}}^{*},{R^{U}}^{*},\;{\beta^{U}}^{*}\right)]$ is the expected final profit of three parties with IBF-B and i=r,s,b. Here, ${q^{U}}^{*}$, ${w^{U}}^{*}$, ${R^{U}}^{*}$, and ${\beta^{U}}^{*}$ are the equilibrium solutions. To be consistent, we subtract $ke^{r_{f}}$ from the retailer’s expected final cash flow to obtain his profits.

We refer to $\frac{E\left[\Pi^{*}\right]}{E\left[\Pi_{dn}^{*}\right]}$ to measure the value of IBF-B in our work. Similar to Lariviere and Porteus [49] and Zhou and Groenevelt [14], we also use two widely employed performance measures: contract efficiency, i.e., $\frac{E\left[\Pi^{*}\right]}{E\left[\Pi_{cn}^{*}\right]}$, and each party’s profit share, i.e., $\frac{E\left[\pi_{i}^{*}\right]}{E\left[\Pi^{*}\right]}$. In addition, we calculate the retailer’s bankruptcy probability $Pr\left[\xi \leq \hat{\xi }\left({q^{U}}^{*}\right)\right]$.

Since our objective is to analyze the value of the IBF-B, the numerical analysis focuses on retailers who intend to apply for IBF-B from the bank. The parameter settings are the same as those in Fig 6. The performance measures are presented in Table 6.

**Table 6:** Supply chain performance for BU-retailers and BB-retailers.

Retailer’s types	k	$\frac{E\left[\Pi^{*}\right]}{E\left[\Pi_{cn}^{*}\right]}$	$\frac{E\left[\Pi^{*}\right]}{E\left[\Pi_{dn}^{*}\right]}$	$Pr\left[\xi \leq \hat{\xi }\left({q^{U}}^{*}\right)\right]$	$\frac{E\left[\pi_{r}^{*}\right]}{E\left[\Pi^{*}\right]}$	$\frac{E\left[\pi_{s}^{*}\right]}{E\left[\Pi^{*}\right]}$	$\frac{E\left[\pi_{b}^{*}\right]}{E\left[\Pi^{*}\right]}$	Whether the retailer uses IBF-B
BU-retailer	0.10	2.50%	–		0.63%	99.37%	–	Not use
	0.50	12.49%			3.33%	96.67%		
	0.90	22.46%			6.35%	93.65%		
	1.30	32.42%			9.77%	90.23%		
	1.35	85.14%	252.89%	0.60%	51.23%	39.89%	8.88%	Use
	1.40	84.60%	242.32%	0.32%	48.56%	46.82%	4.62%	
	1.45	84.04%	232.46%	0.03%	45.85%	53.68%	0.46%	
BB-retailer	1.50	37.29%	–		11.61%	88.39%	–	Not use
	2.00	49.70%			17.01%	82.99%		
	2.50	62.07%			23.77%	76.23%		
	3.00	74.37%			32.78%	67.22%		
	3.50	75.00%			33.33%	66.67%		
	4.00	75.00%			33.33%	66.67%		

**Observation 1:** *As the working capital of BU-retailers and BB-retailers who do not use IBF-B increase, both the contract efficiency and the retailer’s share of the supply chain profit increase. Conversely, the supplier’s share of the supply chain profit declines with the retailer’s increasing working capital.*

When BU-retailers (or BB-retailers) without IBF-B have lower working capital, the supplier captures a larger share of supply chain profit by offering a lower wholesale price to induce higher order quantities (see Fig 6 and Column 7 in Table 6), while retailers obtain only a small share. Additionally, due to the much higher interest rate faced by poorer BU-retailers with k∈[0.1,1.3] (see Table B4 in S1 Appendix B (S1 File)), these retailers do not adopt IBF-B. Consequently, inventory procurement relies solely on limited working capital, resulting in substantially lower order quantities than those under centralized decisions and thus lower contract efficiency. This explains the relatively low contract efficiencies observed for poorer BU-retailers in Table 6.

**Observation 2:** *For richer BU-retailers who adopt IBF-B, IBF-B is highly valuable for enhancing overall supply chain profitability while maintaining a lower bankruptcy probability (see Columns 4 and 5 in* Table 6*). Moreover, the supplier’s share of supply chain profit increases as the retailer’s working capital increases. The shares of supply chain profit of the other two supply chain members, as well as the contract efficiency, decrease with the retailer’s increasing working capital.*

For richer BU-retailers, IBF-B significantly enhances overall supply chain profitability with lower bankruptcy risk, compared to relying solely on their working capital. As shown in Table 6, richer BU-retailers who adopt IBF-B achieve contract efficiencies of 84.04%−85.14%, substantially higher than those of poorer BU-retailers who rely solely on working capital. Nevertheless, the efficiency remains below the centralized benchmark because the supplier’s wholesale price decision, the retailer’s bankruptcy risk, and the bank’s risk-control considerations continue to affect operational and financing decisions. Furthermore, as these retailers’ working capital decreases, they borrow more from the bank, enabling the bank to capture a larger share of supply chain profit and also improving the contract efficiency. Note that the sensitivity analyses in Section 7.3 show that contract efficiency levels reported in Table 6 remain relatively stable under different economic environments. Therefore, the efficiency levels reported in Table 6 are not driven by a specific parameter setting but reflect the effects of capital constraints, financing decisions and operational decisions.

In addition, the loan menu for BU-retailers is shown in S1 Appendix B Table B4 (S1 File). From Table B4, it is noticed that for different levels of the retailer’s working capital, the bank’s optimal inventory advance rate should be set within the range of 57.1%−67.3%, which is highly consistent with the IBF in business practice. For instance, Gibraltar Business Capital, which is a seasoned specialty finance firm with deep roots in asset-based lending, stated that the advance rate is generally around 50% for inventory [50]. And in China, Deputy Secretary-General of China Banking Association, Zhou, said that the average inventory advance rate is generally less than sixty percent [51].

### 7.3 Sensitivity analysis

To further investigate how the economic environment affects the bank’s loan contract decisions, we conduct sensitivity analyses with respect to several key parameters, such as inventory recoverability measured by the salvage-to-price ratio $\mu =\frac{s}{p}$, demand uncertainty reflected by the distribution spread Δ=b−a and the bank’s loan cost rate $r_{c}$. These parameters directly influence the bank’s optimal loan menu including the interest rate and the bounded inventory advance rate. The resulting financing decisions further affect the equilibrium operational decisions and total supply chain performance. Thus, this section investigates the impact of these key parameters on loan terms and contract efficiency.

Note that the retailer’s types are determined by working capital cutoffs, which are themselves affected by the above parameters. Therefore, for each sensitivity analysis, the working capital level is selected from the feasible range under the corresponding parameter setting.

#### 7.3.1 Sensitivity of loan terms to model parameters.

**Fig 7:**
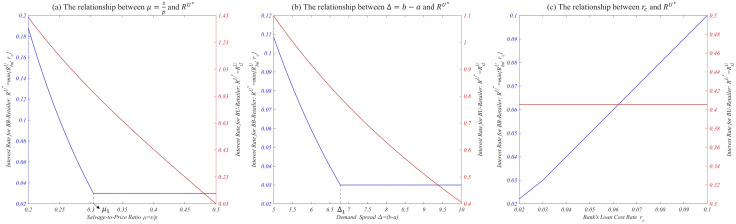
The impact of salvage-to-price ratio (a), Demand Distribution Spread (b), and Bank’s Loan Cost Rate (c) on Interest Rate.

**Fig 8:**
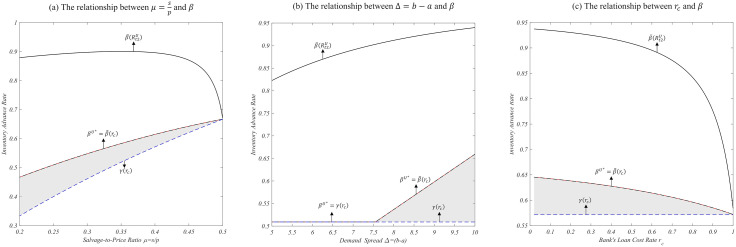
The impact of salvage-to-price ratio (a), Demand Distribution Spread (b), and Bank’s Loan Cost Rate (c) on Inventory Advance Rate.

Figs 7 and 8 illustrate the impacts of salvage-to-price ratio μ, demand spread Δ, and the bank’s loan cost rate $r_{c}$ on the bank’s optimal interest rate and inventory advance rate.

Fig 7(a) shows that the optimal interest rate for BU-retailers decreases as the salvage-to-price ratio μ increases. In contrast, for BB-retailers, the optimal interest rate first decreases and then remains unchanged once μ exceeds $\mu_{\text{1}}$. Intuitively, higher inventory recoverability reduces the bank’s financing risk. However, when the interest rate reaches its lowest feasible level that covers the bank’s loan cost rate, further increases in the salvage-to-price ratio no longer affect the optimal interest rate. Fig 8(a) further shows that since the interior advance rate exceeds the upper bound throughout the parameter range, the optimal advance rate is the upper bound, which increases with μ. A higher salvage-to-price ratio improves the bank’s expected recovery value in the event of retailer bankruptcy, thereby reducing its expected financial losses. Consequently, the bank is willing to offer a lower interest rate and a higher inventory advance rate.

Interestingly, Fig 7(b) and Fig 8(b) jointly reveal a counterintuitive result that as the demand spread increases, the bank lowers the optimal interest rate while increasing the bounded inventory advance rate. In our parameter setting, a larger demand spread generated by a lower demand downside increases the probability of low-demand realizations and retailer default risk. Intuitively, one may expect the bank to tighten loan terms under such conditions. However, excessively restrictive loan terms would reduce the retailer’s order quantity, loan demand, and the bank’s financing return. Consequently, instead of tightening loan terms, the bank is willing to offer a lower interest rate and a higher advance rate to maintain financing attractiveness and stimulate retailers’ ordering under adverse market conditions.

Fig 7(c) shows that the optimal interest rate for BB-retailers increases with the bank’s loan cost rate, whereas that for BU-retailers remains relatively stable. Since BB-retailers intend to borrow below the loan limit, the bank mainly adjusts the interest rate to transfer additional financing cost. However, to significantly control the default risk of the BU-retailer who intends to borrow up to the loan limit, the bank charges the highest feasible interest rate, which is independent of the cost rate $r_{c}$. Fig 8(c) further shows that as the cost rate rises, the bank lowers the bounded inventory advance rate for BU-retailers. Intuitively, a lower advance rate restricts the loan amount and reduces the bank’s financing cost.

#### 7.3.2 Sensitivity of contract efficiency to model parameters.

**Fig 9:**
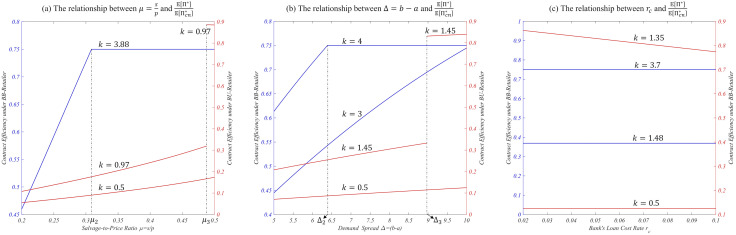
The Impact of Salvage-to-Price Ratio (a), Demand Distribution Spread (b), and Bank’s Loan Cost Rate (c) on Contract Efficiency.

Fig 9 presents how the salvage-to-price ratio μ, demand spread Δ, and the bank’s loan cost rate $r_{c}$ influence the contract efficiency under equilibrium decisions.

As shown in Fig 9(a), only one working capital level, k=3.88, satisfies the BB-retailer region over the entire range μ∈[0.2,0.5]. For this BB-retailer, the contract efficiency increases with μ when $\mu <\mu_{\text{2}}$, since higher inventory recoverability improves the retailer’s expected ordering return. When $\mu \geq \mu_{\text{2}}$, the retailer orders traditional newsvendor quantity, and the contract efficiency remains unchanged. For the BU-retailer with relatively low working capital (k=0.5), the contract efficiency also increases with μ, but remains at a relatively low level because the retailer does not adopt IBF-B and places small orders. In contrast, for the BU-retailer with higher working capital (k=0.97), the retailer adopts IBF-B when $\mu >\mu_{\text{3}}$, resulting in significantly higher contract efficiency. However, the efficiency decreases slightly as μ further increases, indicating that the marginal benefit of IBF-B gradually declines when inventory recoverability becomes sufficiently high.

Fig 9(b) shows that for the BB-retailer with lower working capital (k=3), the contract efficiency always increases as demand spread Δ increases. However, for the BB-retailer with higher working capital (k=4), the contract efficiency first increases then remains unchanged because the equilibrium order quantity reaches the traditional newsvendor quantity. Similar to the impact of salvage-to-price ratio, for the BU-retailer with lower working capital (k=0.5), the contract efficiency also increases with Δ, but remains at a relatively low level. In contrast, for the BU-retailer with higher working capital (k=1.45), the contract efficiency is significantly higher and increases with Δ. Although a larger demand spread implies higher demand uncertainty and thus a higher retailer’s default risk, the bank offers more attractive loan terms to maintain retailer ordering, thereby improving financing accessibility and contract efficiency. Furthermore, for both BB-retailers and BU-retailers, higher working capital makes contract efficiency more sensitive to changes in the salvage-to-price ratio and demand spread.

Fig 9(c) further shows that the contract efficiency decreases as the bank’s loan cost rate rises when the retailer adopts IBF-B. The BB-retailer and BU-retailer with lower working capital (k=0.5) do not adopt IBF-B. Therefore, the contract efficiency under equilibrium decisions is independent of the bank’s loan cost rate.

## 8. Concluding remarks

In this paper, by analyzing the interplay between wholesale price contracts (especially for poorer retailers) determined by suppliers and loan menus offered by banks, we provide novel insights into inventory-based financing with bounded inventory advance rate (IBF-B) for a retailer who faces capital constraints and reveal the value of IBF-B for the supply chain. The main conclusions are as follows.

### 8.1 Managerial insights and theoretical implications

Our findings can guide the decisions of participants in the context of IBF-B. The managerial insights are outlined in the following.

First, we consider that the bank, as a decision-maker with market power over the capital-constrained retailer, can manage the retailer’s default risk in IBF-B by adjusting inventory advance rates that can limit the retailer’s loan amount and order quantity. Building on this finding, we present that the bank should determine an inventory advance rate in the medium range, with the upper and lower bounds derived from an analysis of the retailer’s borrowing decision and bankruptcy risk based on his working capital, so as to balance profitability and credit risk.

Second, we explain the interplay between wholesale price contracts and loan menus according to the retailer’s borrowing decisions. It is demonstrated that the supplier tailors wholesale prices for retailers with various levels of working capital. In the context of IBF-B, the bank can manage credit risk not only by adjusting interest rates but also by controlling inventory advance rates within predetermined bounds. For retailers who intend to borrow below the loan limit, the supplier should choose a wholesale price in its feasible range and maximize her profits, and the bank offers a medium interest rate or the lowest interest rate within the feasible range, and any inventory advance rate within its range, as there is no bankruptcy risk. However, for retailers who intend to borrow up to the loan limit, the supplier chooses a wholesale price contract to obtain higher profits. One induces the retailer to borrow up to the loan limit, and the other enables the retailer to rely solely on working capital. When the retailer borrows up to the loan limit, the order quantity depends solely on the inventory advance rate rather than on the interest rate. Thus, the bank influences the retailer’s optimal order quantity by adjusting the inventory advance rate. If the inventory advance rate contributes positively to the bank’s profit margin, the bank is inclined to choose its upper bound. Otherwise, an interior advance rate within a predetermined range is set. Notably, to improve profitability, the bank consistently charges the highest feasible interest rate to retailers borrowing up to the limit and facing bankruptcy risk, regardless of the value of the inventory advance rate. By simultaneously adjusting both interest rates and inventory advance rates within a predetermined range, the bank can more effectively manage credit risk and enhance financing returns.

Finally, for a uniform demand distribution, we illustrate the interplay between the loan menu and wholesale price contracts and analyze the impact of IBF-B on supply chain performance. The structure of the bank’s loan menu under an endogenous wholesale price is similar to that when the wholesale price is fixed, implying that the fundamental nature of the bank’s decision-making remains unchanged. This further suggests that it is reasonable for the bank to determine IBF-B loan menus based on a given wholesale price. We further find that the bank sets higher inventory advance rates to satisfy as much of the loan demand from the poorer retailers borrowing up to the loan limit. Meanwhile, the bank also imposes higher interest rates to mitigate the bankruptcy risk associated with these retailers. The supplier offers lower wholesale prices to induce these retailers to order more. In addition, the numerical results indicate that, to increase profit, the supplier offers a wholesale price contract that enables retailers with less working capital, who attempt to apply for IBF-B, to place orders using only working capital. By contrast, the retailers with intermediate levels of working capital order by borrowing up to the loan limit. For these retailers, the bank consistently sets the inventory advance rate at its upper bound, a value that aligns with real-world practice. These retailers are also less likely to go bankrupt. The findings indicate that presetting bounds on inventory advance rate is an effective approach for managing credit risk. We also observe that the larger the loan obtained by retailers borrowing up to the loan limit, the higher the supply chain performance. Furthermore, IBF-B is recommended due to its ability to significantly enhance overall supply chain profitability. Sensitivity analyses further show that the effectiveness of IBF-B is closely related to the economic environment. Higher inventory recoverability generally enhances financing accessibility and improves supply chain performance, whereas higher financing costs reduce the effectiveness of IBF-B. Interestingly, under higher demand uncertainty, the bank tends to offer higher inventory advance rates and lower interest rates to improve financing attractiveness and encourage retailers’ ordering.

Moreover, our work also has significant theoretical implications. Inventory-based financing (IBF) is a common way to alleviate the financial pressure of SMEs and has received increasing attention from interfaces of operations and finance. Our research contributes to the existing literature by exploring decisions on inventory-based financing with bounded inventory advance rates, referred to as IBF-B. We then study the interplay between wholesale price contracts and loan menus and confirm the value of IBF-B to the supply chain. We set up a “selling to newsvendor” model to derive the equilibrium on a wholesale price contract between a retailer with limited working capital and an upstream supplier. Meanwhile, it is also proved that there exists an optimal interest rate and an optimal inventory advance rate that determine the bank’s loan menu to maximize its expected final profit.

### 8.2 Limitations and future research

Our paper has some shortcomings, which might be directions for further research. We assume that the collateral value of inventory is evaluated based on the wholesale price. In practice, however, collateral value might be dynamically influenced by market information. Therefore, incorporating dynamic collateral valuation would be a meaningful direction for future research. In addition, we formulate a single-period model for IBF, but multi-period interactions, such as the dynamics of the number of the collateral, can also be considered.

## Supporting information

S1 File Supplementary materials. Appendix A contains the proofs of propositions and lemmas. Appendix B contains the analysis under the uniform demand distribution.
(DOCX)
