# Supplementary material for: Retailer’s inventory-based financing with bounded advance rates: Interplay between wholesale price contract and loan menu
Source: PLoS One. 2026 Jul 30;21(7):e0347675. doi: 10.1371/journal.pone.0347675 (PMC13423192; doi:10.1371/journal.pone.0347675)
Supplement: S1 File — Appendix A contains the proofs of propositions and lemmas. Appendix B contains the analysis under the uniform demand distribution. (DOCX) [file pone.0347675.s001.docx]

# Appendix A. Proofs of statements

**Proof of Lemma 1.** For $\frac{k}{w}\leq q\leq q_{0}$ and $\xi=0$, we have $q\leq\hat{q}$, where $\hat{q}=\frac{k}{w\left( 1-\gamma\right)}$ and $\gamma=\frac{s}{we^{R}}$, satisfied $v_{r}\left( q \right)=sq-l\left( q \right)e^{R}+({k-wq)}^{+}e^{r_{f}}=sq-\left( wq-k \right)e^{R}\geq0$. For $q>q_{0}$ and $\xi=0$, we have $v_{r}\left( q \right)=sq-l(q)e^{R}+({k-wq)}^{+}e^{r_{f}}=sq-\beta wqe^{R}$. It is clear that $\pi_{r}\left( q \right)\geq0$ if $\beta\leq\gamma$.

**Proof of Lemma 2.** For $\hat{q}<q$ and $\xi\leq q$, $v_{r}\left( q \right)=p\xi+sq-s\xi-l\left( q \right)e^{R}+({k-wq)}^{+}e^{r_{f}}\geq0$ if $\xi\geq\hat{\xi}\left( q \right)$, where $\hat{\xi}\left( q \right)=\frac{\left[ l\left( q \right)e^{R}-sq \right]^{+}}{p-s}$ is the bankruptcy threshold.

**Proof of Lemma 3.** As shown in the proof of Lemma 1, it is obvious that $\pi_{r}\left( q \right)\geq0$ if $\beta\leq\gamma$. Then, we pay attention to $\beta>\gamma$ where the retailer may face bankruptcy risk.

1. For $\frac{k}{w}<q\leq\hat{q}$, $\frac{\partial\mathbb{E}\left[ v_{r}\left( q \right) \right]}{\partial q}=\left( p-s \right)\bar{F}\left( q \right)-\left( we^{R}-s \right)$ decreases in $q$, thus $v_{r}\left( q \right)$ is quasi-concave in $q$. We have $q_{2}\left( w,R \right)=\bar{F}^{-1}\left( \frac{we^{R}-s}{p-s} \right)$ which is the solution for $\frac{\partial\mathbb{E}\left[ v_{r}\left( q \right) \right]}{\partial q}=\left( p-s \right)\bar{F}\left( q \right)-\left( we^{R}-s \right)=0$. And since $\hat{q}<q_{0}$, when the retailer’s working capital is $wq_{2}\left( 1-\gamma\right)\leq k<wq_{2}$, the retailer’s optimal order quantity $q_{r}^{*}$ is $q_{2}\left( w,R \right)$ which satisfies $\frac{k}{w}<q_{2}\left( w,R \right)\leq\hat{q}<q_{0}$, where $q_{0}=\frac{k}{\left( 1-\beta\right)w}$ be loan-limit order quantity so that $wq_{0}-k=\beta wq_{0}$.
2. For $\hat{q}<q$, the first order derivative for retailer’s expected final cash flow is $\frac{\partial\mathbb{E}\left[ v_{r}\left( q \right) \right]}{\partial q}=\left( p-s \right)\bar{F}\left( q \right)-\left( we^{R}-s \right)\bar{F}\left( \hat{\xi}\left( q \right) \right)$ and the second order derivative is $\frac{\partial^{2}\mathbb{E}\left[ v_{r}\left( q \right) \right]}{\partial q^{2}}=\left( p-s \right)\bar{F}\left( q \right)\left[ -z\left( q \right)+\frac{we^{R}-s}{p-s}z\left( \hat{\xi}\left( q \right) \right) \right]$, where $z\left( \cdot\right)=\frac{f\left( \cdot\right)}{\bar{F}\left( \cdot\right)}$ is the failure rate of demand distribution. Because $\hat{\xi}\left( q \right)\leq q$ and we assume $z\left( \cdot\right)$ is an increasing function, we derive $-z\left( q \right)+\frac{we^{R}-s}{p-s}z\left( \hat{\xi}\left( q \right) \right)<0$ so that $\frac{\partial^{2}\mathbb{E}\left[ \pi_{r}\left( q \right) \right]}{\partial q^{2}}<0$. Thus $v_{r}\left( q \right)$ is quasi-concave in $q$. The retailer’s loan amount is constrained by the loan limit $\beta wq$. Thus, we solve this problem by using K-K-T condition shown as following.

$$\left\{ \begin{aligned} &\left( p-s \right)\bar{F}\left( q \right)-\left( we^{R}-s \right)\bar{F}\left( \hat{\xi}\left( q \right) \right)-\delta_{1}w\left( 1-\beta\right)=0,\#\left( EC.1a \right) \\ &\delta_{1}\left( k-w\left( 1-\beta\right)q \right)\geq0,\#\left( EC.1b \right) \\ &\delta_{1}\geq0.\#\left( EC.1c \right) \end{aligned} \right.$$

If $\delta_{1}=0$, we have $q_{3}(w,R,k)=\bar{F}^{-1}\left( \frac{we^{R}-s}{p-s}\bar{F}\left( \hat{\xi}\left( q_{3}(w,R,k) \right) \right) \right)$ which is the solution for $\frac{\partial\mathbb{E}\left[ v_{r}\left( q \right) \right]}{\partial q}=\left( p-s \right)\bar{F}\left( q \right)-\left( we^{R}-s \right)\bar{F}\left( \hat{\xi}\left( q \right) \right)=0$. Then, define $T\left( q \right)=\bar{F}^{-1}\left( \frac{we^{R}-s}{p-s}\bar{F}\left( \hat{\xi}\left( q \right) \right) \right)$ and $G\left( q \right)=T\left( q \right)-q$. Since $\bar{F}\left( \cdot\right)$ is continuous and strictly increasing, $G\left( \cdot\right)$ is continuous. Differentiating the identity $\bar{F}\left( T\left( q \right) \right)=\frac{we^{R}-s}{p-s}\bar{F}\left( \hat{\xi}\left( q \right) \right)$ yields $T^{'}\left( q \right)=\left( \frac{we^{R}-s}{p-s} \right)^{2}\frac{f\left( \hat{\xi}\left( q \right) \right)}{f\left( T\left( q \right) \right)}$. Because $0<\frac{we^{R}-s}{p-s}\leq1$, we have $\bar{F}\left( T\left( q \right) \right)\leq\bar{F}\left( \hat{\xi}\left( q \right) \right)$, while implies $T\left( q \right)\geq\hat{\xi}\left( q \right)$. For a demand distribution with IFR, $z\left( \cdot\right)$ is increasing, so $z\left( T\left( q \right) \right)\geq z\left( \hat{\xi}\left( q \right) \right)$. Therefore, $T^{'}\left( q \right)\leq\frac{we^{R}-s}{p-s}<1$ and then $G^{'}\left( q \right)=T^{'}\left( q \right)-1<0$, implying $G\left( q \right)$ is strictly decreasing. Moreover, $G\left( 0 \right)=\bar{F}^{-1}\left( \frac{we^{R}-s}{p-s} \right)>0$, while $G\left( q \right)\to-\infty$ as $q\to\infty$. By intermediate value theorem, there exist a solution $q_{3}$ satisfying $G\left( q_{3} \right)=0$ and the strictly monotonicity of $G\left( q \right)$ guarantees the uniqueness of $q_{3}$. Therefore, the equation $\frac{\partial\mathbb{E}\left[ v_{r}\left( q \right) \right]}{\partial q}=\left( p-s \right)\bar{F}\left( q \right)-\left( we^{R}-s \right)\bar{F}\left( \hat{\xi}\left( q \right) \right)=0$ admits a unique solution in $q$. This establishes the existence and uniqueness of the bound of the inventory advance rate, given by $1-\frac{q_{2}\left( 1-\gamma\right)}{q_{3}}$.

Then, if $1-\frac{q_{2}\left( 1-\gamma\right)}{q_{3}}<\beta\leq1$, for $wq_{3}\left( 1-\beta\right)\leq k<wq_{2}\left( 1-\gamma\right)$, the retailer’s optimal order quantity $q_{r}^{*}$ is $q_{3}(w,R,k)$. And if $\gamma<\beta\leq1-\frac{q_{2}\left( 1-\gamma\right)}{q_{3}}$, we have $wq_{2}\left( 1-\gamma\right)<wq_{3}(1-\beta)$. And for $wq_{2}\left( 1-\gamma\right)\leq k<wq_{2}$, the retailer’s optimal order quantity $q_{r}^{*}$ is $q_{2}\left( w,R \right)$.

If $\delta_{1}>0$, the retailer’s loan amount is constrained by $\beta wq$ and the retailer can only order $q_{0}=\frac{k}{w\left( 1-\beta\right)}.$ Then, from Equation (EC.1a), $\delta_{1}=\frac{\left( p-s \right)\bar{F}\left( q_{0} \right)-\left( we^{R}-s \right)\bar{F}\left( \hat{\xi}\left( q_{0} \right) \right)}{w\left( 1-\beta\right)}$. If $1-\frac{q_{2}\left( 1-\gamma\right)}{q_{3}}<\beta\leq1$, for $k<wq_{3}\left( 1-\beta\right)<wq_{2}\left( 1-\gamma\right)$, we have $q_{3}>q_{0}$. Thus, $\delta_{1}>0$ and the retailer’s optimal order quantity is $q_{0}$. If $\gamma<\beta\leq1-\frac{q_{2}\left( 1-\gamma\right)}{q_{3}}$, for $k<wq_{2}\left( 1-\gamma\right)$, the retailer’s order quantity is $q_{0}$. Since when $1-\frac{q_{2}\left( 1-\gamma\right)}{q_{3}}<\beta\leq1$, the retailer faces higher bankruptcy risk which is not expected by the bank, we focus on $\gamma<\beta\leq1-\frac{q_{2}\left( 1-\gamma\right)}{q_{3}}$ where the retailer faces the medium bankruptcy risk. Note that we let $\hat{\beta}=1-\frac{q_{2}\left( w,R \right)\left( 1-\gamma\left( w,R \right) \right)}{q_{3}\left( w,R,k \right)}$ for simplifying the expression.

In sum, for $\gamma<\beta\leq\hat{\beta}$, when $wq_{2}\left( 1-\gamma\right)\leq k<wq_{2}$, the retailer’s optimal order quantity is $q_{2}\left( w,R \right)$, and when $0\leq k<wq_{2}\left( 1-\gamma\right)$, the retailer’s optimal order quantity is $q_{0}\left( w, \beta,k \right)$.

**Proof of Proposition 1.** The retailers’ decisions for $\frac{k}{w}<q\leq\hat{q}$ and $\hat{q}<q$ are shown in the proof of Lemma 3. Now, we calculate the optimal order quantity for $0\leq q<\frac{k}{w}$ and $q=\frac{k}{w}$.

1. For $0\leq q<\frac{k}{w}$, $\frac{\partial\mathbb{E}\left[ v_{r}\left( q \right) \right]}{\partial q}=\left( p-s \right)\bar{F}\left( q \right)-\left( we^{r_{f}}-s \right)$ decreases in $q$ so that $v_{r}\left( q \right)$ is quasi-concave in $q$. Let $k_{1}\left( w \right)=w\bar{F}^{-1}\left( \frac{we^{r_{f}}-s}{p-s} \right)$. For $k\geq k_{1}\left( w \right)$, we have $\frac{k}{w}\geq\bar{F}^{-1}\left( \frac{we^{r_{f}}-s}{p-s} \right)$. As $v_{r}\left( q \right)$ is quasi-concave in $q$, we have $q_{1}\left( w \right)=\bar{F}^{-1}\left( \frac{we^{r_{f}}-s}{p-s} \right)$ which is the solution for $\frac{\partial\mathbb{E}\left[ v_{r}\left( q \right) \right]}{\partial q}=\left( p-s \right)\bar{F}\left( q_{r}^{*} \right)-\left( we^{r_{f}}-s \right)=0.$ Therefore, for $k>k_{1}\left( w \right)$, the optimal order quantity $q_{r}^{*}$ is $q_{1}\left( w \right)$.
2. For $q=\frac{k}{w}$ and $k\leq k_{1}\left( w \right)$, we have $\frac{k}{w}\leq\bar{F}^{-1}\left( \frac{we^{r_{f}}-s}{p-s} \right)$, then we derive $\left. \frac{\partial\mathbb{E}\left[ v_{r}\left( q \right) \right]}{\partial q} \right|_{{\frac{k}{w}}^{-}}=\left( p-s \right)\bar{F}\left( \frac{k}{w} \right)-\left( we^{r_{f}}-s \right)\geq0$. Because $\frac{\partial\mathbb{E}\left[ v_{r}\left( q \right) \right]}{\partial q}$ decreases in $q$ for $0\leq q<\frac{k}{w}$, $\frac{\partial\mathbb{E}\left[ v_{r}\left( q \right) \right]}{\partial q}=\left( p-s \right)\bar{F}\left( q \right)-\left( we^{r_{f}}-s \right)\geq0$ for any $0\leq q<\frac{k}{w}$. Hence, $q_{1}\left( w \right)$is not in $\left[ 0,\frac{k}{w} \right)$. Then, for $k\geq k_{2}\left( w \right)$, where $k_{2}\left( w \right)=w\bar{F}^{-1}\left( \frac{we^{R}-s}{p-s} \right)$, we have $\frac{k}{w}\geq\bar{F}^{-1}\left( \frac{we^{R}-s}{p-s} \right)$ and thus $\left. \frac{\partial\mathbb{E}\left[ v_{r}\left( q \right) \right]}{\partial q} \right|_{{\frac{k}{w}}^{+}}=\left( p-s \right)\bar{F}\left( \frac{k}{w} \right)-\left( we^{R}-s \right)\leq0$. As a result, for $k_{2}\left( w \right)\leq k\leq k_{1}\left( w \right)$, $q_{r}^{*}=\frac{k}{w}$ is the retailer’s optimal order quantity.

As shown above, the retailer’s optimal order quantity is

$$q_{r}^{*}=\left\{ \begin{aligned} &q_{1}(w),& &if k\geq wq_{1}, \left( no borrowing \right) \\ &\frac{k}{w},&&if wq_{2}\leq k\leq wq_{1}, \left( no borrowing, but using up working capital \right)\# \\ &q_{2}\left( w,R \right),&&if wq_{2}\left( 1-\gamma\right)\leq k<wq_{2},\left( borrowing without bankruptcy risk \right) \\ &q_{0}\left( w, \beta,k \right),&&if 0\leq k<wq_{2}\left( 1-\gamma\right), \left( borrowing with bankruptcy risk \right) \end{aligned} \right..$$

Let $k_{i}\left( w \right)$ denote the retailer’s working capital cutoff, where $i=1,2,3$, $k_{1}\left( w \right)=w\bar{F}^{-1}\left( \frac{we^{r_{f}}-s}{p-s} \right)$, $k_{2}\left( w \right)=w\bar{F}^{-1}\left( \frac{we^{R}-s}{p-s} \right)$ and $k_{3}\left( w \right)=w\left( 1-\gamma\right)\bar{F}^{-1}\left( \frac{we^{R}-s}{p-s} \right)$.

**Proof of** **Proposition 2.** For demand distributions with increasing failure rate, in *UB-Region*, since $q_{r}^{*}=q_{0}\left( w,\beta,k \right)=\frac{k}{w\left( 1-\beta\right)}$ from Equation (3), we have $\frac{dq_{r}^{*}}{dk}=\frac{dq_{0}\left( w,\beta,k \right)}{dk}=\frac{1}{w\left( 1-\beta\right)}>0$. Therefore, the retailer’s optimal order quantity increases in $k$. The bankruptcy threshold in this region is $\hat{\xi}\left( q_{r}^{*} \right)=\frac{l\left( q_{0} \right)e^{R}-sq_{0}}{p-s}$ which increases in $q_{0}$ so that increases in $k$. From the proof of Lemma 3, we have $\frac{\left( p-s \right)\bar{F}\left( q_{0} \right)-\left( we^{R}-s \right)\bar{F}\left( \hat{\xi}\left( q_{0} \right) \right)}{w\left( 1-\beta\right)}>0$. Thus, $\frac{d\mathbb{E}\left[ v_{r}\left( q_{0} \right) \right]}{dk}=\frac{\left[ \left( p-s \right)\bar{F}\left( q_{0} \right)-\left( \beta we^{R}-s \right)\bar{F}\left( \hat{\xi}\left( q_{0} \right) \right) \right]}{w\left( 1-\beta\right)}>0$.

In A-Region, $q_{r}^{*}=\frac{k}{w}$ so that $\frac{dq_{r}^{*}}{dk}=\frac{1}{w}$. Since $\mathbb{E}\left[ v_{r}\left( q_{r}^{*} \right) \right]=\left( p-s \right)\int_{0}^{q} \bar{F}\left( \xi\right)d\xi+sq$ from Equation ([2b](#Eq2b)), we have that $\frac{d\mathbb{E}\left[ \pi_{r}\left( q_{r}^{*} \right) \right]}{dk}=\frac{p\bar{F}\left( q_{r}^{*} \right)+sF\left( q_{r}^{*} \right)}{w}>0$. As a result, both the retailer’s optimal order quantity and expected final cash flow increases with respect to $k$.

**Proof of Lemma 4.** For $q_{r}^{*}=q_{2}\left( w,R \right)$, we have $\bar{F}\left( q_{2}\left( w,R \right) \right)=\frac{we^{R}-s}{p-s}$ and $\frac{d\bar{F}\left( q_{2}\left( w,R \right) \right)}{dR}=\frac{we^{R}}{p-s}>0$. Since $\bar{F}\left( \cdot\right)$ is a strictly decreasing function, then $q_{2}\left( w,R \right)$ decreases with respect to $R$.

For $q_{r}^{*}=q_{0}\left( w,\beta,k \right)$, we have $\frac{dq_{0}\left( w,\beta,k \right)}{d\beta}=\frac{k}{w\left( 1-\beta\right)^{2}}>0$. Therefore, for a given $w$ and $k$, $q_{0}\left( w,\beta,k \right)$ increases with respect to $\beta$.

**Proof of Lemma 5.** First, for $k_{1}\left( w \right)=w\bar{F}^{-1}\left( \frac{we^{r_{f}}-s}{p-s} \right)$, we have $\frac{dk_{1}\left( w \right)}{dw}=\frac{k_{1}\left( w \right)}{w}-\frac{we^{r_{f}}}{\left( we^{r_{f}}-s \right)z\left( \frac{k_{1}\left( w \right)}{w} \right)}$ and

$$\begin{aligned} \frac{d^{2}k_{1}\left( w \right)}{dw^{2}}=\frac{e^{r_{f}}z^{2}\left( \frac{k_{1}\left( w \right)}{w} \right)\left( 2s-we^{r_{f}} \right)-we^{{2r}_{f}}z^{'}\left( \frac{k_{1}\left( w \right)}{w} \right)}{\left( we^{r_{f}}-s \right)^{2}z^{3}\left( \frac{k_{1}\left( w \right)}{w} \right)}.\#\left( EC.2 \right) \end{aligned}$$

Since the denominator of Equation ([EC.2](#ec2)) is positive and the failure rate of demand distributions is increasing and convex, then Equation ([EC.2](#ec2)) is negative only if the salvage value $s\leq\frac{we^{r_{f}}}{2}+\frac{we^{r_{f}}z^{'}\left( \frac{k_{1}\left( w \right)}{w} \right)}{2z\left( \frac{k_{1}\left( w \right)}{w} \right)}=\frac{we^{r_{f}}}{2}+\frac{we^{r_{f}}z^{'}\left( q_{1} \right)}{2z\left( q_{1} \right)}$. In other words, $k_{1}\left( w \right)$ is concave in $w$ only if $s\leq\frac{we^{r_{f}}}{2}+\frac{we^{r_{f}}z^{'}\left( q_{1} \right)}{2z\left( q_{1} \right)}$. The optimal $w$ is solved from $\bar{F}\left( \frac{k_{1}\left( w \right)}{w} \right)=\frac{we^{r_{f}}-s}{p-s}$ and $\frac{k_{1}\left( w \right)}{w}z\left( \frac{k_{1}\left( w \right)}{w} \right)-\frac{we^{r_{f}}}{we^{r_{f}}-s}=0$, simultaneously. The optimal $w$ is denoted by $\tilde{w}$. And let $k_{1}\left( w \right)_{\max}=\tilde{k}$. Then, for $k\leq\tilde{k}$, there exists at most two values of $w$, $0<w_{t1}\leq w_{t2}<pe^{-r_{f}}$, so that $k=k_{1}\left( w \right)$.

Next, for $k_{2}\left( w \right)=w\bar{F}^{-1}\left( \frac{we^{R}-s}{p-s} \right)$, we have $\frac{dk_{2}\left( w \right)}{dw}=\frac{k_{2}\left( w \right)}{w}-\frac{we^{R}}{\left( we^{R}-s \right)z\left( \frac{k_{2}\left( w \right)}{w} \right)}$ and

$$\begin{aligned} \frac{d^{2}k_{2}\left( w \right)}{dw^{2}}=\frac{e^{R}z^{2}\left( \frac{k_{2}\left( w \right)}{w} \right)\left( 2s-we^{R} \right)-we^{2R}z^{'}\left( \frac{k_{2}\left( w \right)}{w} \right)}{\left( we^{R}-s \right)^{2}z^{3}\left( \frac{k_{2}\left( w \right)}{w} \right)}.\#\left( EC.3 \right) \end{aligned}$$

It is clear that Equation ([EC.3](#ec3)) is negative only if $s\leq\frac{we^{R}}{2}+\frac{we^{R}z^{'}\left( q_{2} \right)}{2z\left( q_{2} \right)}$, which means $k_{2}\left( w \right)$ is concave in $w$ only if $s\leq\frac{we^{R}}{2}+\frac{we^{R}z^{'}\left( q_{2} \right)}{2z\left( q_{2} \right)}$. The optimal $w$ is solved from $\bar{F}\left( \frac{k_{2}\left( w \right)}{w} \right)=\frac{we^{R}-s}{p-s}$ and $\frac{k_{2}\left( w \right)}{w}z\left( \frac{k_{2}\left( w \right)}{w} \right)-\frac{we^{R}}{we^{R}-s}=0$ simultaneously. The optimal $w$ is denoted by $\bar{w}$. Let $k_{2}\left( w \right)_{\max}=\bar{k}$. Hence, for $k\leq\bar{k}$, there exists at most two values of $w$, $w_{t3}\leq w_{t4}$, so that $k=k_{2}\left( w \right)$. Since $R\geq r_{f}$, then $k_{1}\left( w \right)\geq k_{2}\left( w \right)$. From the definition of $w_{t1}$ and $w_{t2}$, we know that $k=k_{1}\left( w_{ti} \right)$, where $i=1,2$. Therefore, for $i=1,2$, we have $k\geq k_{2}\left( w_{ti} \right)$ so that $w_{t1}\leq w_{t3}\leq w_{t4}\leq w_{t2}$.

Finally, for $k_{3}\left( w \right)=w\left( 1-\gamma\right)\bar{F}^{-1}\left( \frac{we^{R}-s}{p-s} \right)$, we have $\frac{dk_{3}\left( w \right)}{dw}=\frac{k_{3}\left( w \right)}{w}-\frac{we^{R}\left( 1-\gamma\right)}{\left( we^{R}-s \right)z\left( \frac{k_{3}\left( w \right)}{w\left( 1-\gamma\right)} \right)}$ and

$$\begin{aligned} \frac{d^{2}k_{3}\left( w \right)}{dw^{2}}=\frac{e^{R}{\left( 1-\gamma\right)z}^{2}\left( \frac{k_{3}\left( w \right)}{w\left( 1-\gamma\right)} \right)\left( 2s-we^{R} \right)-we^{2R}\left( 1-\gamma\right)z^{'}\left( \frac{k_{3}\left( w \right)}{w\left( 1-\gamma\right)} \right)}{\left( we^{R}-s \right)^{2}z^{3}\left( \frac{k_{3}\left( w \right)}{w\left( 1-\gamma\right)} \right)}.\#\left( EC.4 \right) \end{aligned}$$

We obtain that $k_{3}\left( w \right)$ is concave in $w$ only if $s\leq\frac{we^{R}}{2}+\frac{we^{R}z^{'}\left( q_{2} \right)}{2z\left( q_{2} \right)}$. The optimal $w$ is solved from $\bar{F}\left( \frac{k_{3}\left( w \right)}{w\left( 1-\gamma\right)} \right)=\frac{we^{R}-s}{p-s}$ and $\frac{k_{3}\left( w \right)}{w}z\left( \frac{k_{3}\left( w \right)}{w\left( 1-\gamma\right)} \right)-\frac{we^{R}\left( 1-\gamma\right)}{we^{R}-s}=0$ simultaneously. The optimal $w$ is denoted by $\underline{w}$. Let $k_{3}\left( w \right)_{\max}= \underline{k}$. Therefore, for $k\leq\underline{k}$, there exists at most two values of $w$, $w_{t5}\leq w_{t6}$, so that $k=k_{3}\left( w \right)$. Since $\gamma\leq1$, then $k_{2}\left( w \right)\geq k_{3}\left( w \right)$. From the definition of $w_{t3}$ and $w_{t4}$, we know that $k=k_{2}\left( w_{ti} \right)$, where $i=3,4$. Consequently, for $i=3,4$, we have $k\geq k_{3}\left( w_{ti} \right)$ so that $w_{t3}\leq w_{t5}\leq w_{t6}\leq w_{t4}$.

**Proof of Proposition 3.** Simplifying the writing, for a given $k$, $R$ and $\beta$, let $q_{2}\left( w,R \right)=q_{2}\left( w \right)$ and $q_{0}\left( w,\beta,k \right)=q_{0}(w)$. It is not difficult to show that in *T-Region*, *A-Region* and *UB-Region*, the optimal order quantity $q_{r}^{*}(w)$ decreases in $w$, because $\frac{\partial q_{1}\left( w \right)}{\partial w}=-\frac{e^{r_{f}}}{\left( p-s \right)f\left( q_{1}\left( w \right) \right)}<0$, $\frac{\partial\frac{k}{w}}{\partial w}=-\frac{k}{w^{2}}<0$ and $\frac{\partial q_{0}\left( w \right)}{\partial w}=-\frac{k}{\left( 1-\beta\right)w^{2}}<0$. Then in *BN-Region*, $\frac{\partial q_{2}\left( w \right)}{\partial w}=-\frac{e^{R}}{\left( p-s \right)f\left( q_{2}\left( w \right) \right)}<0$ so that $q_{r}^{*}(w)$ decreases in$w$. Therefore, the optimal order quantitydecreases in wholesale price.

**Proof of Proposition 4.** For $\bar{k}\leq k\leq\tilde{k}$, if $w\leq w_{t1}$ or $w\geq w_{t2}$, the retailer behaves as the traditional newsvendor and the optimal order quantity $q_{s1}$ satisfies the following condition (Lariviere & Porteus, 2001).

$$\begin{aligned} \bar{F}\left( q_{s1} \right)-q_{s1}f\left( q_{s1} \right)-\frac{ce^{r_{f}}-s}{p-s}=0,\#\left( EC.5 \right) \end{aligned}$$

for $w\leq w_{t1}$ or $w\geq w_{t2}$. The corresponding wholesale price, denoted by $w_{s1}$, is $w_{s1}=\left[ \left( p-s \right)\bar{F}\left( q_{s1} \right)+s \right]e^{-r_{f}}$. Let $\tilde{q}$ satisfies $\tilde{q}z\left( \tilde{q} \right)=\frac{we^{r_{f}}}{we^{r_{f}}-s}>1$ and the corresponding wholesale price is $\tilde{w}=\left[ \left( p-s \right)\bar{F}\left( \hat{q} \right)+s \right]e^{-r_{f}}$. If $ce^{r_{f}}>s>0$, then $q_{s1}z\left( q_{s1} \right)<1$. From the proof of Lemma 6, we have $q_{s1}\leq\tilde{q}\leq q_{t1}$ and $w_{s1}\geq\tilde{w}\geq w_{t1}>0$.

In the *A-Region*, the supplier gains the total wealth of the retailer. We obtain that $\frac{\partial\pi_{s}\left( q_{r}^{*} \right)}{\partial q_{r}^{*}}=-ce^{r_{f}}<0$ in this case and $q_{r}^{*}\left( w \right)$ decreases in $w$ from Proposition 3, so the supplier’s final profit increases in $w$. As a result, the equilibrium wholesale price is $w_{t2}$ in this region. If $w_{t2}\leq w_{s1}$, $w_{s1}$ is the global equilibrium wholesale price and the corresponding order quantity is $q_{s1}$. Otherwise, the global equilibrium wholesale price is $w_{t2}$ and the corresponding order quantity is $q_{t2}$. And from Proposition 3, we have $q_{t2}\geq q_{s1}$ when $w_{t2}\leq w_{s1}$.

**Proof of Proposition 5.** Considering Equation (6), we obtain that the first derivate of $\pi_{s}\left( q_{r}^{*} \right)$ with respect to $q_{r}^{*}$ is

$$\begin{aligned} \frac{\partial\pi_{s}\left( q_{r}^{*} \right)}{\partial q_{r}^{*}}=-\left( p-s \right)e^{r_{f}-R}\left[ q_{r}^{*}f\left( q_{r}^{*} \right)-\bar{F}\left( q_{r}^{*} \right)+\frac{ce^{R}-s}{p-s} \right].\#\left( EC.6 \right) \end{aligned}$$

Lariviere and Porteus (2001) have established that $\frac{\partial\pi_{s}\left( q_{r}^{*} \right)}{\partial q_{r}^{*}}$ decreases in $q_{r}^{*}\left( w \right)$. Hence, we defined that $q_{s2}$ is the solution of $\bar{F}\left( q_{s2} \right)-q_{s2}f\left( q_{s2} \right)-\frac{ce^{R}-s}{p-s}=0$, which means the equilibrium order quantity is $q_{s2}$ with corresponding wholesale price $w_{s2}$. Since $ce^{R}>s>0$, we have $q_{s2}z\left( q_{s2} \right)<1$. Similar to discussion in section 5.2.1, from Lemma 6, we have $q_{s2}\leq q_{s1}\leq\tilde{q}\leq q_{t3}\leq q_{t1}$ and $w_{s2}\geq\tilde{w}\geq w_{t3}\geq w_{t1}$.

Notice that if and only if $w_{s1}$ is in *T-Region*, i.e., $w_{t2}\leq w_{s1}$, $\left( w_{s1},q_{s1} \right)$ is the candidate of global equilibrium. Otherwise, $w_{t2}>w_{s1}$, the candidate is $(w_{t2},q_{t2})$. Furthermore, we also have to decide whether $w_{s2}$ is not exceed the $w_{t4}$. If $w_{s2}$ is lower than $w_{t4}$, $\left( w_{s2},q_{s2} \right)$ is one of the global equilibrium candidates. The supplier offers a wholesale price contract which can maximizing her final profit with satisfying the region condition.

**Proof of Proposition 6.** Due to $\frac{\partial\pi_{s}\left( q_{r}^{*} \right)}{\partial q_{r}^{*}}=-ce^{r_{f}}<0$ and the results from Proposition 3, the supplier’s final profits increase in $w$. Consequently, the supplier charges $w_{t6}$ to enable the retailer’s loan requirement to reach the loan limit and place larger orders, thereby increasing her profits. From the Proof in Proposition 5, the wholesale price contract $\left( w_{s1},q_{s1} \right)$ and $(w_{t2},q_{t2})$ cannot be the global equilibrium candidates simultaneously. And for the BU-retailer, $\left( w_{t6},q_{0}\left( w_{t6} \right) \right)$ is also a global equilibrium candidate. The supplier offers a wholesale price contract which can maximizing her final profit with satisfying the region condition.

**Proof of Lemma 6.** First, for $k_{2}\left( R \right)=w\bar{F}^{-1}\left( \frac{we^{R}-s}{p-s} \right)$, we have $\frac{dk_{2}\left( R \right)}{dR}=-\frac{w^{2}e^{R}}{\left( we^{R}-s \right)z\left( \frac{k_{2}\left( R \right)}{w} \right)}<0$ so that $k_{2}\left( R \right)$ decreases in $R$. Since $r_{c}\leq R\leq\ln\left( \frac{p}{w} \right)$, then $k_{2}\left( R \right)$ takes the maximum value at $r_{c}$, i.e., $k_{2}\left( R \right)_{\max}=k_{2}\left( r_{c} \right)$. Therefore, we obtain that for $k\leq k_{2}\left( r_{c} \right)$, there exists an interest rate $R_{t1}$ so that $k_{2}\left( R_{t1} \right)=k$, where $r_{c}\leq R_{t1}\leq\ln\left( \frac{p}{w} \right)$.

Next, for $k_{3}\left( R \right)=w\left( 1-\gamma\right)\bar{F}^{-1}\left( \frac{we^{R}-s}{p-s} \right)$, we have $\frac{dk_{3}\left( R \right)}{dR}=\frac{w[\gamma q_{2}z\left( q_{2} \right)-1]}{z\left( q_{2} \right)}$. $q_{2}\bar{F}\left( q_{2} \right)=q_{2}\frac{we^{R}-s}{p-s}$ increases in $q_{2}$ so that $q_{2}\leq\check{q}$, where $\check{q}$ satisfies $\check{q}z\left( \check{q} \right)=1$. Since the failure rate of demand distribution is increasing and convex, then $q_{2}z\left( q_{2} \right)\leq\check{q}z\left( \check{q} \right)=1$ so that $\frac{dk_{3}\left( R \right)}{dR}<0$. Therefore, $k_{3}\left( R \right)$ takes the maximum value at $r_{c}$, i.e., $k_{3}\left( R \right)_{\max}=k_{3}\left( r_{c} \right)$. As a result, we obtain that for $k\leq k_{3}\left( r_{c} \right)$, there exists an interest rate $R_{t2}$, so that $k_{2}\left( R_{t2} \right)=k$, where $r_{c}\leq R_{t2}\leq R_{t1}$.

**Proof of Proposition 7.** For the demand distribution with increasing and convex failure rate and a given $w$, (1) and for $k_{3}\left( r_{c} \right)<k\leq k_{2}\left( r_{c} \right)$, the retailer borrows below the loan limit without bankruptcy risk, thus, the inventory advance rate offered by the bank can be up to 1. The bank’s problem is shown as Equation ([EC.7](#ec7)), which is subjected to $r_{c}\leq R\leq R_{t1}$ and $\gamma\left( r_{c} \right)<\beta\leq1$.

$$\mathbb{E}\left[ \pi_{b}\left( R \right) \right]=\max\left( wq_{2}\left( R \right)-k \right)\left( e^{R}-e^{r_{c}} \right)$$

$$s.t. r_{c}\leq R\leq R_{t1},$$

$$\begin{aligned} \gamma\left( r_{c} \right)<\beta\leq1.\#\left( EC.7 \right) \end{aligned}$$

We solve this problem by using K-K-T condition shown as following.

$$\left\{ \begin{aligned} &w\frac{dq_{2}\left( R \right)}{dR}\left( e^{R}-e^{r_{c}} \right)+\left( wq_{2}\left( R \right)-k \right)e^{R}-\theta_{3}+\theta_{4}=0 \\ &-\theta_{1}+\theta_{2}=0 \\ &\theta_{1}(1-\beta)=0 \\ &\theta_{2}\left( \beta-\gamma\left( r_{c} \right) \right)=0 \\ &\theta_{3}(R_{t1}-R)=0 \\ &\theta_{4}(R-r_{c})=0 \\ &\theta_{i}\geq0, where i=1,2,3,4. \end{aligned} \right.$$

There are several cases for the solution of the above system of equations.

1. From the second and the last equations in the system of equations, we derive $\theta_{1}=\theta_{2}=0$. First, let $\theta_{3}=\theta_{4}=0$, then we have $w\frac{dq_{2}\left( R \right)}{dR}\left( e^{R}-e^{r_{c}} \right)+\left( wq_{2}\left( R \right)-k \right)e^{R}=0$ and $R_{bu}$ is the solution for this equation. Since all constraints in this problem are linear, we should examine whether $\mathbb{E}\left[ \pi_{b}\left( R \right) \right]$ is concave in $R$. The second order derivative of the bank’s expected final profit with respect to interest rate $\frac{d^{2}\mathbb{E}\left[ \pi_{b}\left( R \right) \right]}{dR^{2}}=w\left( e^{R}-e^{r_{c}} \right)\frac{d^{2}q_{2}\left( R \right)}{dR^{2}}+2we^{R}\frac{dq_{2}\left( R \right)}{dR}+\left( wq_{2}\left( R \right)-k \right)e^{R}$ where $\frac{d^{2}q_{2}\left( R \right)}{dR^{2}}=\frac{we^{R}\left[ sz\left( q_{2}\left( R \right) \right)^{2}-we^{R}z^{'}\left( q_{2}\left( R \right) \right) \right]}{\left( we^{R}-s \right)^{2}z^{3}\left( q_{2}\left( R \right) \right)}$. Let $g_{0}\left( r_{c} \right)=w\left( e^{R_{bu}}-e^{r_{c}} \right)\frac{d^{2}q_{2}\left( R \right)}{dR^{2}}\left. \right|_{R=R_{bu}}+2we^{R_{bu}}\frac{dq_{2}\left( R \right)}{dR}\left. \right|_{R=R_{bu}}+\left( wq_{2}\left( R_{bu} \right)-k \right)e^{R_{bu}}$. When the cost rate of bank loans $r_{c}$ satisfies $g_{0}\left( r_{c} \right)<0$, which implies $\frac{d^{2}\mathbb{E}\left[ \pi_{b}\left( R \right) \right]}{dR^{2}}\left. \right|_{R=R_{bu}}<0$, $R_{bu}$ is the optimal candidate interest rate.
2. Let $\theta_{3}>0$ and $\theta_{4}=0$, we rewrite the first equation to derive $\theta_{3}=w\frac{dq_{2}\left( R \right)}{dR}\left( e^{R}-e^{r_{c}} \right)+\left( wq_{2}\left( R \right)-k \right)e^{R}$. And for $\theta_{3}>0$, we have $R=R_{t1}$. However, when $R=R_{t1}$, $\theta_{3}=w\frac{dq_{2}\left( R \right)}{dR}\left. \right|_{R=R_{t1}}\left( e^{R_{t1}}-e^{r_{c}} \right)+\left( wq_{2}\left( R_{t1} \right)-k \right)e^{R_{t1}}<0$. Thus, $R=R_{t1}$ is not the optimal solution for the bank’s problem and $\theta_{3}=0$.
3. Let $\theta_{3}=0$ and $\theta_{4}>0$, we rewrite the first equation to derive $\theta_{4}=-w\frac{dq_{2}\left( R \right)}{dR}\left( e^{R}-e^{r_{c}} \right)-\left( wq_{2}\left( R \right)-k \right)e^{R}$. And for $\theta_{4}>0$, we have $R=r_{c}.$ However, when $R=R_{t1}$, $\theta_{4}=-\left( wq_{2}\left( r_{c} \right)-k \right)e^{r_{c}}\geq0$. Thus, $R=r_{c}$ is the candidate optimal solution for the bank’s problem.

In sum, we derive the bank’s optimal credit decision for the retailer who borrows below the loan limit and has no bankruptcy risk. That is, if $R_{bu}>r_{c}$, $R^{*}=R_{bu}$, otherwise $R^{*}=r_{c}$, and $\gamma\left( r_{c} \right)<\beta^{*}<1$, where $R_{bu}$ satisfies $\left( wq_{2}\left( w,R_{bu} \right)-k \right)\left( we^{R_{bu}}-s \right)z\left( q_{2}\left( {w,R}_{bu} \right) \right)-w^{2}\left( e^{R_{bu}}-e^{r_{c}} \right)=0$.

(2) From the proof of Lemma 3, the inventory advance rate should satisfy $wq_{3}\left( 1-\beta\right)\geq wq_{2}\left( 1-\gamma\right)$. Then from the proof of Lemma 7, $k_{3}\left( R \right)_{max}=k_{3}\left( r_{c} \right)=w\left( 1-\gamma\left( r_{c} \right) \right)q_{2}\left( r_{c} \right)$. Thus, the inventory advance rate should satisfy $wq_{3}\left( r_{c} \right)\left( 1-\beta\right)\geq wq_{2}\left( r_{c} \right)\left( 1-\gamma\left( r_{c} \right) \right)$ to enable the retailer to have bankruptcy risk only when he borrows up to the loan limit. Thus, the bank intends to constrain the inventory advance rate within a medium range, i.e., $\gamma\left( r_{c} \right)<\beta\leq\hat{\beta}\left( r_{c} \right)$. The bank’s problem is shown as Equation ([EC.8](#ec8)), which is subjected to $r_{c}\leq R\leq R_{t2}$ and $\gamma\left( r_{c} \right)<\beta\leq\hat{\beta}\left( r_{c} \right)$.

$$\mathbb{E}\left[ \pi_{b}\left( R,\beta\right) \right]=\max\beta wq_{0}\left( \beta\right)\left( e^{R}-e^{r_{c}} \right)-\left( p-s \right)\left( \hat{\xi}\left( q_{0}\left( \beta\right),R \right)-\int_{0}^{\hat{\xi}\left( q_{0}\left( \beta\right),R \right)} \bar{F}\left( \xi\right)d\xi\right)$$

$$s.t. r_{c}\leq R\leq R_{t2},$$

$$\begin{aligned} \gamma\left( r_{c} \right)<\beta\leq\hat{\beta}\left( r_{c} \right).\#\left( EC.8 \right) \end{aligned}$$

Then, we solve this problem by using K-K-T condition shown as following.

$$\left\{ \begin{aligned} &\beta wq_{0}e^{R}-\left( p-s \right)\left( \frac{d\hat{\xi}\left( q_{0}\left( \beta\right),R \right)}{dR}-\bar{F}\left( \hat{\xi}\left( q_{0} \right) \right)\frac{d\hat{\xi}\left( q_{0}\left( \beta\right),R \right)}{dR} \right)-\lambda_{3}+\lambda_{4}=0 \\ &wq_{0}\left( e^{R}-e^{r_{c}} \right)+\beta w\left( e^{R}-e^{r_{c}} \right)\frac{dq_{0}}{d\beta}-\left( p-s \right)\left( \frac{d\hat{\xi}\left( q_{0}\left( \beta\right),R \right)}{d\beta}-\bar{F}\left( \hat{\xi}\left( q_{0} \right) \right)\frac{d\hat{\xi}\left( q_{0}\left( \beta\right),R \right)}{d\beta} \right)-\lambda_{1}+\lambda_{2}=0 \\ &\lambda_{1}(\hat{\beta}\left( r_{c} \right)-\beta)=0 \\ &\lambda_{2}\left( \beta-\gamma\left( r_{c} \right) \right)=0 \\ &\lambda_{3}(R_{t2}-R)=0 \\ &\lambda_{4}(R-r_{c})=0 \\ &\lambda_{i}\geq0, where i=1,2,3,4. \end{aligned} \right.$$

There are several cases for the solution of the above system of equations.

1. Let $\lambda_{i}=0$. The first and second equations in the system of equations can be rewritten as the follows. $\beta wq_{0}e^{R}-\left( p-s \right)\left( \frac{d\hat{\xi}\left( q_{0}\left( \beta\right),R \right)}{dR}-\bar{F}\left( \hat{\xi} \right)\frac{d\hat{\xi}\left( q_{0}\left( \beta\right),R \right)}{dR} \right)=\beta wq_{0}e^{R}\bar{F}\left( \hat{\xi} \right)>0$ and $wq_{0}\left( e^{R}-e^{r_{c}} \right)+\beta w\left( e^{R}-e^{r_{c}} \right)\frac{dq_{0}}{d\beta}-\left( p-s \right)\left( \frac{d\hat{\xi}\left( q_{0}\left( \beta\right),R \right)}{d\beta}-\bar{F}\left( \hat{\xi}\left( q_{0} \right) \right)\frac{d\hat{\xi}\left( q_{0}\left( \beta\right),R \right)}{d\beta} \right)=\frac{q_{0}\left[ \left( we^{R}-s \right)\bar{F}\left( \hat{\xi}\left( q_{0}\left( w,\beta,k \right) \right) \right)-\left( we^{r_{c}}-s \right) \right]}{\left( 1-\beta\right)}=0$. Since $\frac{d\mathbb{E}\left[ \pi_{b}\left( R,\beta\right) \right]}{dR}=\beta wq_{0}e^{R}\bar{F}\left( \hat{\xi}\left( q_{0} \right) \right)>0$, the bank sets the highest feasible interest rate to optimize his expected final profit, i.e., $R=R_{t2}$, which results in $\lambda_{3}>0$. Note that because the bank’s expected final profit is strictly increasing with respect to $R$ over the available range, the optimal interest rate is always attained at the upper bound $R_{t2}$. Thus, the original two-dimensional problem reduces to one-dimensional constrained maximization problem in $\beta$. As a result, the KKT conditions with respect to $\beta$ are sufficient to characterize the global optimum, without requiring second-order conditions or Hessian matrix checks for $R$ and $\beta$.
2. Then, for $\lambda_{3}>0$ and $\lambda_{1}=\lambda_{2}=\lambda_{4}=0$, $\beta=\bar{\beta}\left( R \right)$ is the solution of $\frac{q_{0}\left( w,\beta,k \right)\left[ \left( we^{R}-s \right)\bar{F}\left( \hat{\xi}\left( q_{0}\left( w,\beta,k \right) \right) \right)-\left( we^{r_{c}}-s \right) \right]}{\left( 1-\beta\right)}=0$ and $R=R_{t2}$. Since $\left. \frac{d^{2}\mathbb{E}\left[ \pi_{b}\left( R,\beta\right) \right]}{d\beta^{2}} \right|_{\beta=\bar{\beta}\left( R \right)}=\frac{-\frac{q_{0}\left( w,\bar{\beta}\left( R \right),k \right)^{2}\left( we^{R}-s \right)^{2}f\left( \hat{\xi}\left( q_{0}\left( w,\bar{\beta}\left( R \right),k \right) \right) \right)}{p-s}}{\left( 1-\bar{\beta}\left( R \right) \right)^{2}}<0$, $\bar{\beta}\left( R \right)$ can optimize $\mathbb{E}\left[ \pi_{b}\left( R,\beta\right) \right]$. In this case, $\left( R_{t2},\bar{\beta}\left( R_{t2} \right) \right)$ is the candidate optimal loan menu.
3. When $\lambda_{1}>0$ and $\lambda_{3}>0$ and $\lambda_{2}=\lambda_{4}=0$, we have $R=R_{t2}$ and $\beta=\hat{\beta}\left( r_{c} \right)$.If $\lambda_{1}\left( R_{t2},\hat{\beta}\left( r_{c} \right) \right)=\frac{q_{0}\left( w,\hat{\beta}\left( r_{c} \right),k \right)\left[ \left( we^{R_{t2}}-s \right)\bar{F}\left( \hat{\xi}\left( q_{0}\left( w,\hat{\beta}\left( r_{c} \right),k \right) \right) \right)-\left( we^{r_{c}}-s \right) \right]}{\left( 1-\hat{\beta}\left( r_{c} \right) \right)}>0$, $\left( R_{t2}, \hat{\beta}\left( r_{c} \right) \right)$ is the candidate optimal loan menu.
4. When $\lambda_{4}>0$ and $\lambda_{1}=\lambda_{2}=\lambda_{3}=0$, the first equation in the system of equations can be rewritten as $\beta wq_{0}e^{R}\bar{F}\left( \hat{\xi}\left( q_{0} \right) \right)+\lambda_{4}=0$. Since $\beta wq_{0}e^{R}\bar{F}\left( \hat{\xi}\left( q_{0} \right) \right)>0$, we have $\lambda_{4}<0$ which contradicts $\lambda_{4}>0$.

In addition, since our research focuses on the bank’s inventory advance rate decision within a medium region characterized by $\gamma\left( r_{c} \right)<\beta\leq\hat{\beta}\left( r_{c} \right)$, we restrict attention to the parameter region in which the cost rate of bank loans $r_{c}$ satisfies $\bar{\beta}\left( R_{t2} \right)>\gamma\left( r_{c} \right)$. Under this condition, the candidate interior solution belongs to retailer’s bankruptcy region.

In sum, we derive the bank’s optimal credit decision for the retailer who borrows up to the loan limit and has bankruptcy risk. That is, when $r_{c}$ satisfies $\bar{\beta}\left( R_{t2} \right)>\gamma\left( r_{c} \right)$, if $\lambda_{1}\left( R_{t2},\hat{\beta}\left( r_{c} \right) \right)>0$, the bank’s optimal loan menu is $\left( R_{t2},\hat{\beta}\left( r_{c} \right) \right)$ where $\lambda_{1}\left( R_{t2},\hat{\beta}\left( r_{c} \right) \right)=\frac{q_{0}\left( w,\hat{\beta}\left( r_{c} \right),k \right)\left[ \left( we^{R_{t2}}-s \right)\bar{F}\left( \hat{\xi}\left( q_{0}\left( w,\hat{\beta}\left( r_{c} \right),k \right) \right) \right)-\left( we^{r_{c}}-s \right) \right]}{\left( 1-\hat{\beta}\left( r_{c} \right) \right)}$. Otherwise, the bank offers $\left( R_{t2},\bar{\beta}\left( R_{t2} \right) \right)$ as optimal loan menu, where $\bar{\beta}\left( R_{t2} \right)$ satisfies $\left( we^{R_{t2}}-s \right)\bar{F}\left( \hat{\xi}\left( q_{0}\left( w,\bar{\beta}\left( R_{t2} \right),k \right) \right) \right)-\left( we^{r_{c}}-s \right)=0$.

# Appendix B. The analysis for the uniform demand distribution

Assume that the demand $\xi$ follows a uniform distribution with $\xi\sim U(a,b)$. We study the bank’s optimal credit decision when the supplier offers wholesale price contracts are $\left( w^{*},q^{*} \right)$ to the retailer who applies for IBF-B from the bank.

The BB-retailer retailer will apply for IBF-B from the bank if the wholesale price contract is $\left( w_{s2},q_{s2} \right)$, where $w_{s2}=\frac{\left( bp-as \right)+ce^{R}\left( b-a \right)}{2\left( b-a \right)e^{R}}$, $q_{s2}=\frac{\left( bp-as \right)-ce^{R}\left( b-a \right)}{2\left( p-s \right)}$. And the BU-retailer with $0\leq k\leq\underline{k}\left( R \right)$ will place an order $q_{0}\left( w_{t6} \right)$ by borrowing up to the loan limit, when the wholesale price is $w_{t6}$, where $w_{t6}=\frac{\sqrt{\left( p-s \right)\left( b^{2}\left( p-s \right)-4ke^{R}\left( b-a \right) \right)}+b\left( p+s \right)-2as}{2e^{R}\left( b-a \right)}$ and $q_{0}\left( w_{t6} \right)=\frac{k}{w_{t6}\left( 1-\beta\right)}$.

Following the analysis in Section 6.2, we derive the same results about the relationship between the working capital cutoffs ($\bar{k}\left( R \right)$ and $\underline{k}\left( R \right)$) and interest rates for the uniform demand distribution in Lemma [EC.1](#Lemmaec1).

**Lemma EC.1.** *Both* $\bar{k}\left( R \right)$*and* $\underline{k}\left( R \right)$*decrease with* $R$ *Thus,* $\bar{k}\left( R \right)_{max}=\bar{k}\left( r_{c} \right)=\frac{\left( bp-as \right)^{2}}{4e^{r_{c}}\left( p-s \right)\left( b-a \right)}$*,* $\underline{k}\left( R \right)_{max}=\underline{k}\left( r_{c} \right)=\frac{b^{2}\left( p-s \right)}{4e^{r_{c}}\left( b-a \right)}$*, and* $\underline{k}\left( R \right)_{max}\leq\bar{k}\left( R \right)_{max}$*. For* $\leq k_{i}\left( R \right)_{max}$ $(i=2,3)$*, there exists a value for* $R$ *so that the retailer exhausts all working capital to determine optimal order quantity, regardless of whether borrowing is necessary. That is*

*(1) For* $\underline{k}\left( r_{c} \right)<k\leq\bar{k}\left( r_{c} \right)$*, there exists a value of* $R$*, i.e.,* $r_{c}\leq R_{t1}^{U}\leq\ln\left( \frac{p}{w} \right)$*, so that* $\bar{k}\left( R_{t1}^{U} \right)=k$*, where* $R_{t1}^{U}=\ln\left( \frac{\left( bp-as \right)^{2}}{4\left( p-s \right)\left( b-a \right)k} \right)$*;*

*(2) For* $0\leq k\leq\underline{k}\left( r_{c} \right)$*, there exists a value of* $R$*, i.e.,* $r_{c}\leq R_{t2}^{U}\leq R_{t1}^{U}$*, so that* $\underline{k}\left( R_{t2}^{U} \right)=k$*, where* $R_{t2}^{U}=\ln\left( \frac{b^{2}\left( p-s \right)}{4\left( b-a \right)k} \right)$.

**Proof of Lemma EC.1.** For the demand $\xi$ follows a uniform distribution with $\xi\sim U(a,b)$, $\bar{k}\left( R \right)=\frac{\left( bp-as \right)^{2}}{4e^{R}\left( p-s \right)\left( b-a \right)}$ and $\underline{k}\left( R \right)=\frac{b^{2}\left( p-s \right)}{4e^{R}\left( b-a \right)}$. Then, we have $\frac{d\bar{k}\left( R \right)}{dR}=-\frac{\left( bp-as \right)^{2}}{4e^{R}\left( p-s \right)\left( b-a \right)}<0$ and $\frac{d\underline{k}\left( R \right)}{dR}=-\frac{b^{2}\left( p-s \right)}{4e^{R}\left( b-a \right)}<0$. Thus, $\bar{k}\left( R \right)_{max}=\bar{k}\left( r_{c} \right)$, $\underline{k}\left( R \right)_{max}=\underline{k}\left( r_{c} \right)$. We obtain for $\underline{k}\left( r_{c} \right)<k\leq\bar{k}\left( r_{c} \right)$, there exists an interest rate $R_{t1}^{U}$ so that $\bar{k}\left( R_{t1}^{U} \right)=k$, where $R_{t1}^{U}=\ln\left( \frac{\left( bp-as \right)^{2}}{4\left( p-s \right)\left( b-a \right)k} \right)$ and $r_{c}\leq R_{t1}^{U}\leq\ln\left( \frac{p}{w} \right)$; and for $0\leq k\leq\underline{k}\left( r_{c} \right)$, there exists an interest rate $R_{t2}^{U}$ so that $\underline{k}\left( R_{t2}^{U} \right)=k$, where $R_{t2}^{U}=\ln\left( \frac{b^{2}\left( p-s \right)}{4\left( b-a \right)k} \right)$ and $r_{c}\leq R_{t2}^{U}{\leq R}_{t1}^{U}$.

**Proof of Proposition 8.** For the uniform demand distribution $\xi\sim U(a,b)$, (1) and for $\underline{k}\left( r_{c} \right)<k\leq\bar{k}\left( r_{c} \right)$, when the wholesale price contract offered by the supplier is $\left( w_{s2},q_{s2} \right)$, the retailer borrows below the loan limit without bankruptcy risk. Thus, the inventory advance rate offered by the bank can be up to 1. The bank’s problem is shown as Equation ([EC.9](#eqec9)), which is subjected to $r_{c}\leq R\leq R_{t1}^{U}$ and $\gamma\left( r_{c} \right)<\beta\leq1$.

$$\mathbb{E}\left[ \pi_{b}\left( R \right) \right]=\max\left( w_{s2}\left( R \right)q_{s2}\left( R \right)-k \right)\left( e^{R}-e^{r_{c}} \right)$$

$$s.t. r_{c}\leq R\leq R_{t1}^{U},$$

$$\begin{aligned} \gamma\left( r_{c} \right)<\beta\leq1\#\left( EC.9 \right) \end{aligned}$$

We solve this problem by using K-K-T condition shown as following.

$$\left\{ \begin{aligned} &\left( \frac{dw_{s2}\left( R \right)}{dR}q_{s2}\left( R \right)+w_{s2}\left( R \right)\frac{dq_{s2}\left( R \right)}{dR} \right)\left( e^{R}-e^{r_{c}} \right)+\left( w_{s2}\left( R \right)q_{s2}\left( R \right)-k \right)e^{R}-\theta_{3}^{'}+\theta_{4}^{'}=0 \\ &-\theta_{1}^{'}+\theta_{2}^{'}=0 \\ &\theta_{1}^{'}(1-\beta)=0 \\ &\theta_{2}^{'}\left( \beta-\gamma\left( r_{c} \right) \right)=0 \\ &\theta_{3}^{'}(R_{t1}^{U}-R)=0 \\ &\theta_{4}^{'}(R-r_{c})=0 \\ &\theta_{i}^{'}\geq0, where i=1,2,3,4. \end{aligned} \right.$$

There are several cases for the solution of the above system of equations.

1. From the second and the last equations in the system of equations, we derive $\theta_{1}^{'}=\theta_{2}^{'}=0$. First, let $\theta_{3}^{'}=\theta_{4}^{'}=0$, then we have $\left( \frac{dw_{s2}\left( R \right)}{dR}q_{s2}\left( R \right)+w_{s2}\left( R \right)\frac{dq_{s2}\left( R \right)}{dR} \right)\left( e^{R}-e^{r_{c}} \right)+\left( w_{s2}\left( R \right)q_{s2}\left( R \right)-k \right)e^{R}=\frac{\left( e^{R}-e^{r_{c}} \right)\left( c\left( b-a \right)e^{R}\left( \sigma_{2}\left( R \right)-\sigma_{3}\left( R \right) \right)-\sigma_{2}\left( R \right)\sigma_{3}\left( R \right) \right)-e^{R}\left( \sigma_{1}ke^{R}-\sigma_{2}\left( R \right)\sigma_{3}\left( R \right) \right)}{\sigma_{1}e^{R}}=0$ and $R_{bu}^{U}$ is the solution for this equation, where $\sigma_{1}=4\left( b-a \right)\left( p-s \right)$, $\sigma_{2}\left( R \right)=\left( bp-as \right)-ce^{R}\left( b-a \right)$ and $\sigma_{3}\left( R \right)=\left( bp-as \right)+ce^{R}\left( b-a \right)$. Since all constraints in this problem are linear, we should examine whether $\mathbb{E}\left[ \pi_{b}\left( R \right) \right]$ is concave in $R$. The second order derivative of the bank’s expected final profit with respect to interest rate $\frac{d^{2}\mathbb{E}\left[ \pi_{b}\left( R \right) \right]}{dR^{2}}=\left( \frac{d^{2}w_{s2}\left( R \right)}{dR^{2}}q_{s2}\left( R \right)+2\frac{dw_{s2}\left( R \right)}{dR}\frac{dq_{s2}\left( R \right)}{dR}+w_{s2}\left( R \right)\frac{d^{2}q_{s2}\left( R \right)}{dR^{2}} \right)\left( e^{R}-e^{r_{c}} \right)+2\left( \frac{dw_{s2}\left( R \right)}{dR}q_{s2}\left( R \right)+w_{s2}\left( R \right)\frac{dq_{s2}\left( R \right)}{dR} \right)+\left( w_{s2}\left( R \right)q_{s2}\left( R \right)-k \right)e^{R}=\frac{c\left( b-a \right)e^{2R}\left[ \left( \sigma_{2}\left( R \right)-\sigma_{3}\left( R \right) \right)-2ce^{R}\left( b-a \right) \right]-e^{r_{c}}\left[ \sigma_{2}\left( R \right)\sigma_{3}\left( R \right)-2c^{2}e^{2R}\left( b-a \right)^{2}-c\left( b-a \right)\left( \sigma_{2}\left( R \right)-\sigma_{3}\left( R \right) \right)e^{R} \right]-\sigma_{1}ke^{2R}}{\sigma_{1}e^{R}}$, where $\sigma_{1}=4\left( b-a \right)\left( p-s \right)$, $\sigma_{2}\left( R \right)=\left( bp-as \right)-ce^{R}\left( b-a \right)$ and $\sigma_{3}\left( R \right)=\left( bp-as \right)+ce^{R}\left( b-a \right)$. Let $h_{0}\left( r_{c} \right)=-4\left( b-a \right)^{2}c^{2}e^{3R_{bu}^{U}}+\left( b-a \right)^{2}c^{2}e^{2R_{bu}^{U}}e^{r_{c}}-4\left( b-a \right)\left( p-s \right)ke^{2R_{bu}^{U}}-\left( bp-as \right)^{2}e^{r_{c}}$. When the cost rate of bank loans $r_{c}$ satisfies $h_{0}\left( r_{c} \right)<0$, which implies $\frac{d^{2}\mathbb{E}\left[ \pi_{b}\left( R \right) \right]}{dR^{2}}\left. \right|_{R=R_{bu}^{U}}<0$, $R_{bu}^{U}$ is the optimal candidate interest rate.
2. Let $\theta_{3}^{'}>0$ and $\theta_{4}^{'}=0$, we rewrite the first equation to derive $\theta_{3}^{'}=\left( \frac{dw_{s2}\left( R \right)}{dR}q_{s2}\left( R \right)+w_{s2}\left( R \right)\frac{dq_{s2}\left( R \right)}{dR} \right)\left( e^{R}-e^{r_{c}} \right)+\left( w_{s2}\left( R \right)q_{s2}\left( R \right)-k \right)e^{R}$. And when $\theta_{3}^{'}>0$, we have $R=R_{t1}^{U}$. Since $\frac{dw_{s2}\left( R \right)}{dR}q_{s2}\left( R \right)+w_{s2}\left( R \right)\frac{dq_{s2}\left( R \right)}{dR}=\frac{-\left( bp-as \right)^{2}-\left( b-a \right)^{2}c^{2}e^{2R}}{4\left( b-a \right)\left( p-s \right)e^{R}}<0$ and $R_{t1}^{U}=\ln\left( \frac{\left( bp-as \right)^{2}}{4\left( p-s \right)\left( b-a \right)k} \right)$, we have $\theta_{3}^{'}\left( R_{t1}^{U} \right)=\left( \frac{dw_{s2}\left( R \right)}{dR}\left. \right|_{R=R_{t1}^{U}}q_{s2}\left( R_{t1}^{U} \right)+w_{s2}\left( R_{t1}^{U} \right)\frac{dq_{s2}\left( R \right)}{dR}\left. \right|_{R=R_{t1}^{U}} \right)\left( e^{R_{t1}^{U}}-e^{r_{c}} \right)+\left( w_{s2}\left( R_{t1}^{U} \right)q_{s2}\left( R_{t1}^{U} \right)-k \right)e^{R_{t1}^{U}}<0$, which contradicts $\theta_{3}^{'}>0$. Thus, $R=R_{t1}^{U}$ is not the optimal solution for the bank’s problem and $\theta_{3}^{'}=0$.
3. Let $\theta_{3}^{'}=0$ and $\theta_{4}^{'}>0$, we rewrite the first equation to derive $\theta_{4}^{'}=-\left( \frac{dw_{s2}\left( R \right)}{dR}q_{s2}\left( R \right)+w_{s2}\left( R \right)\frac{dq_{s2}\left( R \right)}{dR} \right)\left( e^{R}-e^{r_{c}} \right)-\left( w_{s2}\left( R \right)q_{s2}\left( R \right)-k \right)e^{R}$. And when $\theta_{4}^{'}>0$, we have $R=r_{c}.$ However, when $R=r_{c}$, $\theta_{4}^{'}=-\left( w_{s2}\left( r_{c} \right)q_{s2}\left( r_{c} \right)-k \right)e^{r_{c}}$, and for $\underline{k}\left( r_{c} \right)<k\leq\bar{k}\left( r_{c} \right)$, we have $\theta_{4}^{'}>0$. Thus, $R=r_{c}$ is the optimal solution for the bank’s problem and $\theta_{4}^{'}=0$.

In sum, we derive the bank’s optimal credit decision for the retailer who borrows below the loan limit and has no bankruptcy risk. That is, if $R_{bu}^{U}>r_{c}$, ${R^{U}}^{*}=R_{bu}^{U}$, otherwise, ${R^{U}}^{*}=r_{c}$,

and $\gamma\left( r_{c} \right)<\beta^{U*}<1$, where $R_{bu}^{U}$ *satisfies* $\frac{\left( e^{R_{bu}^{U}}-e^{r_{c}} \right)\left( c\left( b-a \right)e^{R_{bu}^{U}}\left( \sigma_{2}\left( R_{bu}^{U} \right)-\sigma_{3}\left( R_{bu}^{U} \right) \right)-\sigma_{2}\left( R_{bu}^{U} \right)\sigma_{3}\left( R_{bu}^{U} \right) \right)-e^{R_{bu}^{U}}\left( \sigma_{1}ke^{R_{bu}^{U}}-\sigma_{2}\left( R_{bu}^{U} \right)\sigma_{3}\left( R_{bu}^{U} \right) \right)}{\sigma_{1}e^{R_{bu}^{U}}}=0$*.*

(2) For $0<k\leq\underline{k}\left( r_{c} \right)$, when the wholesale price contract offered by the supplier is $\left( w_{t6},q_{0}\left( w_{t6} \right) \right)$, the retailer borrows up to the loan limit with the bankruptcy risk. Thus, the inventory advance rate is constrained within a medium range, i.e., $\gamma\left( r_{c} \right)<\beta\leq\hat{\beta}\left( r_{c} \right)$. Then, the bank’s problem is shown as Equation ([EC.10](#eqec10)), which is subjected to $r_{c}\leq R\leq R_{t2}^{U}$ and $\gamma\left( r_{c} \right)<\beta\leq\hat{\beta}\left( r_{c} \right)$.

$$\mathbb{E}\left[ \pi_{b}\left( R,\beta\right) \right]=\max\beta w_{t6}\left( R \right)q_{0}\left( w_{t6}\left( R \right),\beta\right)\left( e^{R}-e^{r_{c}} \right)-\left( p-s \right)\left( \hat{\xi}\left( q_{0}\left( w_{t6}\left( R \right),\beta\right),R \right)-\int_{0}^{\hat{\xi}\left( q_{0}\left( w_{t6}\left( R \right),\beta\right),R \right)} \bar{F}\left( \xi\right)d\xi\right)$$

$$s.t. r_{c}\leq R\leq R_{t2}^{U},$$

$$\begin{aligned} \gamma\left( r_{c} \right)<\beta\leq\hat{\beta}\left( r_{c} \right),\#\left( EC.10 \right) \end{aligned}$$

We solve this problem by using K-K-T condition shown as following.

$$\left\{ \begin{aligned} &\left( \beta\frac{dw_{t6}\left( R \right)}{dR}q_{0}\left( w_{t6}\left( R \right),\beta\right)e^{R}+\beta w_{t6}\left( R \right)\frac{dq_{0}\left( w_{t6}\left( R \right),\beta\right)}{dw_{t6}}\frac{dw_{t6}\left( R \right)}{dR} \right)\left( e^{R}-e^{r_{c}} \right)+\beta w_{t6}\left( R \right)q_{0}\left( w_{t6}\left( R \right),\beta\right)e^{R} \\ -\left( p-s \right)\left( \frac{d\hat{\xi}\left( q_{0}\left( w_{t6}\left( R \right),\beta\right),R \right)}{dR}-\bar{F}\left( \hat{\xi} \right)\frac{d\hat{\xi}\left( q_{0}\left( w_{t6}\left( R \right),\beta\right),R \right)}{dR} \right)-\lambda_{3}^{'}+\lambda_{4}^{'}=0 \\ &w_{t6}\left( R \right)q_{0}\left( w_{t6}\left( R \right),\beta\right)\left( e^{R}-e^{r_{c}} \right)+\beta w_{t6}\left( R \right)\left( e^{R}-e^{r_{c}} \right)\frac{dq_{0}\left( w_{t6}\left( R \right),\beta\right)}{d\beta} \\ -\left( p-s \right)\left( \frac{d\hat{\xi}\left( q_{0}\left( w_{t6}\left( R \right),\beta\right),R \right)}{d\beta}-\bar{F}\left( \hat{\xi} \right)\frac{d\hat{\xi}\left( q_{0}\left( w_{t6}\left( R \right),\beta\right),R \right)}{d\beta} \right)-\lambda_{1}^{'}+\lambda_{2}^{'}=0 \\ &\lambda_{1}^{'}(\hat{\beta}\left( r_{c} \right)-\beta)=0 \\ &\lambda_{2}^{'}\left( \beta-\gamma\left( r_{c} \right) \right)=0 \\ &\lambda_{3}^{'}(R_{t2}^{U}-R)=0 \\ &\lambda_{4}^{'}(R-r_{c})=0 \\ &\lambda_{i}^{'}\geq0, where i=1,2,3,4. \end{aligned} \right.$$

There are several cases for the solution of the above system of equations.

1. Let $\lambda_{i}^{'}=0$. The first and second equations in the system of equations can be rewritten as $\beta w_{t6}\left( R \right)q_{0}\left( w_{t6}\left( R \right),\beta\right)e^{R}\bar{F}\left( \hat{\xi}\left( q_{0}\left( w_{t6}\left( R \right),\beta\right) \right) \right)+\left( 1-\bar{F}\left( \hat{\xi}\left( q_{0}\left( w_{t6}\left( R \right),\beta\right) \right) \right) \right)s\frac{dq_{0}\left( w_{t6}\left( R \right),\beta\right)}{dR}$ and $w_{t6}\left( R \right)q_{0}\left( w_{t6}\left( R \right),\beta\right)\left( e^{R}-e^{r_{c}} \right)+\beta w_{t6}\left( R \right)\left( e^{R}-e^{r_{c}} \right)\frac{dq_{0}\left( w_{t6}\left( R \right),\beta\right)}{d\beta}-\left( p-s \right)\left( \frac{d\hat{\xi}\left( q_{0}\left( w_{t6}\left( R \right),\beta\right),R \right)}{d\beta}-\bar{F}\left( \hat{\xi} \right)\frac{d\hat{\xi}\left( q_{0}\left( w_{t6}\left( R \right),\beta\right),R \right)}{d\beta} \right)=\frac{q_{0}\left( w_{t6}\left( R \right),\beta\right)\left[ \left( w_{t6}e^{R}-s \right)\bar{F}\left( \hat{\xi}\left( q_{0}\left( w_{t6}\left( R \right),\beta\right) \right) \right)-\left( we^{r_{c}}-s \right) \right]}{\left( 1-\beta\right)}$. Since $\frac{dq_{0}\left( w_{t6}\left( R \right),\beta\right)}{dR}=\frac{k\left[ \left( b\left( p+s \right)-2as \right)\sqrt{\left( p-s \right)\left( b^{2}\left( p-s \right)-4ke^{R}\left( b-a \right) \right)}+\left( p-s \right)\left( b^{2}\left( p-s \right)-2\left( b-a \right)ke^{R} \right) \right]}{2\left( b-a \right)e^{R}\sqrt{\left( p-s \right)\left( b^{2}\left( p-s \right)-4ke^{R}\left( b-a \right) \right)}w_{t6}^{2}\left( R \right)\left( 1-\beta\right)}>0$, we have $\frac{d\mathbb{E}\left[ \pi_{b}\left( R,\beta\right) \right]}{dR}=\beta w_{t6}\left( R \right)q_{0}\left( w_{t6}\left( R \right),\beta\right)e^{R}\bar{F}\left( \hat{\xi}\left( q_{0}\left( w_{t6}\left( R \right),\beta\right) \right) \right)+\left( 1-\bar{F}\left( \hat{\xi}\left( q_{0}\left( w_{t6}\left( R \right),\beta\right) \right) \right) \right)s\frac{dq_{0}\left( w_{t6}\left( R \right),\beta\right)}{dR}>0$. Thus, the bank sets the highest feasible interest rate to optimize his expected final profit, i.e., $R=R_{t2}^{U}=\ln\left( \frac{b^{2}\left( p-s \right)}{4\left( b-a \right)k} \right)$, which results in $\lambda_{3}^{'}>0$. Note that because the bank’s expected final profit is strictly increasing with respect to $R$ over the available range, the optimal interest rate is always attained at the upper bound $R_{t2}^{U}$. Thus, the original two-dimensional problem reduces to one-dimensional constrained maximization problem in $\beta$. As a result, the KKT conditions with respect to $\beta$ are sufficient to characterize the global optimum, without requiring second-order conditions or Hessian matrix checks for $R$ and $\beta$.
2. Then, for $\lambda_{3}^{'}>0$ and $\lambda_{1}^{'}=\lambda_{2}^{'}=\lambda_{4}^{'}=0$, $\beta=\bar{\beta}\left( R_{t2}^{U} \right)$ is the solution of $\frac{q_{0}\left( w_{t6}\left( R_{t2}^{U} \right),\beta\right)\left[ \left( w_{t6}\left( R_{t2}^{U} \right)e^{R_{t2}^{U}}-s \right)\bar{F}\left( \hat{\xi}\left( q_{0}\left( w_{t6}\left( R_{t2}^{U} \right),\beta\right) \right) \right)-\left( we^{r_{c}}-s \right) \right]}{\left( 1-\beta\right)}=0$. Since $\left. \frac{d^{2}\mathbb{E}\left[ \pi_{b}\left( R,\beta\right) \right]}{d\beta^{2}} \right|_{\beta=\bar{\beta}\left( R_{t2}^{U} \right)}=\frac{{-q}_{0}\left( w_{t6}\left( R_{t2}^{U} \right),\bar{\beta}\left( R_{t2}^{U} \right) \right)^{2}\left( w_{t6}\left( R_{t2}^{U} \right)e^{R_{t2}^{U}}-s \right)^{2}f\left( \hat{\xi}\left( q_{0}\left( w_{t6}\left( R_{t2}^{U} \right),\bar{\beta}\left( R_{t2}^{U} \right) \right) \right) \right)}{\left( 1-\bar{\beta}\left( R_{t2}^{U} \right) \right)^{2}\left（ p-s \right）}<0$, $\bar{\beta}\left( R_{t2}^{U} \right)$ can optimize $\mathbb{E}\left[ \pi_{b}\left( R,\beta\right) \right]$. Then, $\left( R_{t2}^{U},\bar{\beta}\left( R_{t2}^{U} \right) \right)$ is the candidate optimal loan menu, where $\bar{\beta}\left( R_{t2}^{U} \right)=\frac{2\left( s+\sigma_{5} \right)\left( b-a \right)}{\sigma_{4}+2\left( b-a \right)\sigma_{5}}$, $\sigma_{4}=b\left( p+s \right)-2as$and $\sigma_{5}=\frac{2\sigma_{4}\left[ b^{4}\left( p-s \right)^{2}+2\left( b-a \right)^{2}\left( sb^{2}\left( p-s \right)-2ke^{r_{c}}\sigma_{4} \right) \right]}{b^{5}\left( p-s \right)^{2}}.$
3. When $\lambda_{1}^{'}>0$, $\lambda_{3}^{'}>0$and $\lambda_{2}^{'}=\lambda_{4}^{'}=0$, we have $R=R_{t2}^{U}$and $\beta=\hat{\beta}\left( r_{c} \right)$ if $\lambda_{1}^{'}=\frac{q_{0}\left( w_{t6}\left( R_{t2}^{U} \right),\hat{\beta}\left( r_{c} \right) \right)\left[ \left( w_{t6}\left( R_{t2}^{U} \right)e^{R_{t2}^{U}}-s \right)\bar{F}\left( \hat{\xi}\left( q_{0}\left( w_{t6}\left( R_{t2}^{U} \right),\hat{\beta}\left( r_{c} \right) \right) \right) \right)-\left( w_{t6}\left( R_{t2}^{U} \right)e^{r_{c}}-s \right) \right]}{\left( 1-\hat{\beta}\left( r_{c} \right) \right)}>0$. Thus, in this case, if $\lambda_{1}^{'}\left( R_{t2}^{U}, \hat{\beta}\left( r_{c} \right) \right)>0$, $\left( R_{t2}^{U}, \hat{\beta}\left( r_{c} \right) \right)$ is the candidate optimal loan menu, where $\hat{\beta}\left( r_{c} \right)=1-\left( \frac{2s\left( b-a \right)\left( \sigma_{6}^{2}-\left( p-s \right)^{2} \right)}{\left( \left( b\left( p-s \right)+ke^{r_{c}} \right)\sigma_{6}+b\left( p-s \right)^{2} \right)\sigma_{4}}+1 \right)\frac{b}{2}$ and $\sigma_{6}=-\frac{b\left( p-s \right)}{2\left( b-a \right)}$.
4. When $\lambda_{4}^{'}>0$ and $\lambda_{1}^{'}=\lambda_{2}^{'}=\lambda_{3}^{'}=0$, the first equation in the system of equations can be rewritten as $\beta w_{t6}\left( R \right)q_{0}\left( w_{t6}\left( R \right),\beta\right)e^{R}\bar{F}\left( \hat{\xi}\left( q_{0}\left( w_{t6}\left( R \right),\beta\right) \right) \right)+\left( 1-\bar{F}\left( \hat{\xi}\left( q_{0}\left( w_{t6}\left( R \right),\beta\right) \right) \right) \right)s\frac{dq_{0}\left( w_{t6}\left( R \right),\beta\right)}{dR}+\lambda_{4}^{'}=0$. Since $\beta w_{t6}\left( R \right)q_{0}\left( \beta,R \right)e^{R}\bar{F}\left( \hat{\xi}\left( q_{0}\left( \beta,R \right) \right) \right)+\left( 1-\bar{F}\left( \hat{\xi}\left( q_{0}\left( \beta,R \right) \right) \right) \right)s\frac{dq_{0}\left( \beta,R \right)}{dR}>0$, we have $\lambda_{4}^{'}<0$ which contradicts $\lambda_{4}^{'}>0$.

In addition, since our research focuses on the bank’s inventory advance rate decision within a medium region characterized by $\gamma\left( r_{c} \right)<\beta\leq\hat{\beta}\left( r_{c} \right)$, we restrict attention to the parameter region in which the cost rate of bank loans $r_{c}$ satisfies $\bar{\beta}\left( R_{t2}^{U} \right)>\gamma\left( r_{c} \right)$. That is, $\bar{\beta}\left( R_{t2}^{U} \right)- \gamma\left( r_{c} \right)=\frac{2\left( b-a \right)\sigma_{5}\left( r_{c} \right)b\left( p-s \right)}{\left[ \sigma_{4}+2\left( b-a \right)\sigma_{5}\left( r_{c} \right) \right]\sigma_{4}}>0$. Under this condition, the candidate interior solution belongs to retailer’s bankruptcy region.

We summarize the bank’s optimal loan menu in Table B1 and show the expression of all candidate wholesale price contracts in Table B2.

Table B1. The expressions of the optimal loan menu.

| The type of the retailer | BU-retailers with  $0<k\leq\underline{k}\left( r_{c} \right)$ | | BB-retailers with  $\underline{k}\left( r_{c} \right)<k\leq\bar{k}\left( r_{c} \right)$ | |
| --- | --- | --- | --- | --- |
| Condition | $\lambda_{1}^{'}\left( R_{t2}^{U},\hat{\beta}\left( r_{c} \right) \right)>0$ | $\lambda_{1}^{'}\left( R_{t2}^{U},\hat{\beta}\left( r_{c} \right) \right)\leq0$ | $R_{bu}^{U}>r_{c}$ | $R_{bu}^{U}\leq r_{c}$ |
| Interest rate ${R^{U}}^{*}$ | $R_{t2}^{U}=\ln\left( \frac{b^{2}\left( p-s \right)}{4\left( b-a \right)k} \right)$ | | $R_{bu}^{U}$ | $r_{c}$ |
| Inventory advance rate ${\beta^{U}}^{*}$ | $\hat{\beta}\left( r_{c} \right)=1-\left( \frac{2s\left( b-a \right)\left( \sigma_{6}^{2}-\left( p-s \right)^{2} \right)}{\left( \left( b\left( p-s \right)+ke^{r_{c}} \right)\sigma_{6}+b\left( p-s \right)^{2} \right)\sigma_{4}}+1 \right)\frac{b}{2}$ | $\bar{\beta}\left( R_{t2}^{U} \right)=\frac{2\left( s+\sigma_{5} \right)\left( b-a \right)}{\sigma_{4}+2\left( b-a \right)\sigma_{5}}$ | $\gamma\left( r_{c} \right)<{\beta^{U}}^{*}<\hat{\beta}\left( r_{c} \right)$ | |

*Note:* $\sigma_{4}=b\left( p+s \right)-2as$*,* $\sigma_{5}=\frac{2\sigma_{4}\left[ b^{4}\left( p-s \right)^{2}+2\left( b-a \right)^{2}\left( sb^{2}\left( p-s \right)-2ke^{r_{c}}\sigma_{4} \right) \right]}{b^{5}\left( p-s \right)^{2}}$*,* and $\sigma_{6}=-\frac{b\left( p-s \right)}{2\left( b-a \right)}$*. And* $R_{bu}^{U}$ *satisfies* $\frac{\left( e^{R_{bu}^{U}}-e^{r_{c}} \right)\left( c\left( b-a \right)e^{R_{bu}^{U}}\left( \sigma_{2}\left( R_{bu}^{U} \right)-\sigma_{3}\left( R_{bu}^{U} \right) \right)-\sigma_{2}\left( R_{bu}^{U} \right)\sigma_{3}\left( R_{bu}^{U} \right) \right)-e^{R_{bu}^{U}}\left( \sigma_{1}ke^{R_{bu}^{U}}-\sigma_{2}\left( R_{bu}^{U} \right)\sigma_{3}\left( R_{bu}^{U} \right) \right)}{\sigma_{1}e^{R_{bu}^{U}}}=0$*, where* $\sigma_{1}=4\left( b-a \right)\left( p-s \right)$, $\sigma_{2}\left( R_{bu}^{U} \right)=\left( bp-as \right)-ce^{R_{bu}^{U}}\left( b-a \right)$ and $\sigma_{3}\left( R_{bu}^{U} \right)=\left( bp-as \right)+ce^{R_{bu}^{U}}\left( b-a \right)$.

Table B2. The expressions of all candidate wholesale price contracts.

| Wholesale price contract | |
| --- | --- |
| Wholesale price | Order quantity |
| $w_{s1}=\frac{\left( bp-as \right)+ce^{r_{f}}\left( b-a \right)}{2\left( b-a \right)e^{r_{f}}}$ | $q_{s1}=\frac{\left( bp-as \right)-ce^{r_{f}}\left( b-a \right)}{2\left( p-s \right)}$ |
| $w_{t2}=\frac{\left( bp-as \right)+\sqrt{\left( bp-as \right)^{2}-4\left( b-a \right)\left( p-s \right)ke^{r_{f}}}}{2\left( b-a \right)e^{r_{f}}}$ | $q_{t2}=\frac{\left( bp-as \right)-\sqrt{\left( bp-as \right)^{2}-4\left( b-a \right)\left( p-s \right)ke^{r_{f}}}}{2\left( p-s \right)}$ |
| $w_{s2}\left( {R^{U}}^{*} \right)=\frac{\sigma_{3}\left( {R^{U}}^{*} \right)}{2\left( b-a \right)e^{{R^{U}}^{*}}}$  where ${R^{U}}^{*}=\max\left\{ R_{bu}^{U}, r_{c} \right\}$ | $q_{s2}\left( {R^{U}}^{*} \right)=\frac{\sigma_{2}\left( {R^{U}}^{*} \right)}{2\left( p-s \right)}$ |
| $w_{t6}\left( R_{t2}^{U} \right)=\frac{2k\sigma_{4}}{b^{2}\left( p-s \right)}$ | $q_{0}\left( R_{t2}^{U},\beta^{U^{*}} \right)=\frac{b^{2}\left( p-s \right)}{2\sigma_{4}\left( 1-{\beta^{U}}^{*} \right)}$  where $\beta^{U^{*}}=\min\left\{ \bar{\beta}\left( R_{t2}^{U} \right),\hat{\beta}\left( r_{c} \right) \right\}$ |

**Proof of Proposition 9.** Since $R_{bu}^{U}$ satisfies $\frac{\left( e^{R_{bu}^{U}}-e^{r_{c}} \right)\left( c\left( b-a \right)e^{R_{bu}^{U}}\left( \sigma_{2}\left( R_{bu}^{U} \right)-\sigma_{3}\left( R_{bu}^{U} \right) \right)-\sigma_{2}\left( R_{bu}^{U} \right)\sigma_{3}\left( R_{bu}^{U} \right) \right)-e^{R_{bu}^{U}}\left( \sigma_{1}ke^{R_{bu}^{U}}-\sigma_{2}\left( R_{bu}^{U} \right)\sigma_{3}\left( R_{bu}^{U} \right) \right)}{\sigma_{1}e^{R_{bu}^{U}}}=0$, we have $k=\frac{\left( e^{R_{bu}^{U}}-e^{r_{c}} \right)e^{R_{bu}^{U}}c\left( b-a \right)\left( \sigma_{2}\left( R_{bu}^{U} \right)-\sigma_{3}\left( R_{bu}^{U} \right) \right)+e^{r_{c}}\sigma_{2}\left( R_{bu}^{U} \right)\sigma_{3}\left( R_{bu}^{U} \right)}{\sigma_{1}e^{{2R}_{bu}^{U}}}$ and $\frac{dR_{bu}^{U}}{dk}=\frac{1}{\frac{dk}{dR_{bu}^{U}}}=\frac{\sigma_{1}e^{{2R}_{bu}^{U}}}{-2c^{2}e^{3R_{bu}^{U}}\left( b-a \right)^{2}-\left( bp-as \right)^{2}e^{r_{c}}}<0$.

Then, we have $\frac{dq_{s2}\left( R_{bu}^{U} \right)}{dk}=-\frac{ce^{R_{bu}^{U}}\left( b-a \right)}{2\left( p-s \right)}\frac{dR_{bu}^{U}}{dk}>0,$ $\frac{dw_{s2}\left( R_{bu}^{U} \right)}{dk}=-\frac{bp-as}{2\left( b-a \right)e^{R_{bu}^{U}}}\frac{dR_{bu}^{U}}{dk}>0,$ $\frac{dR_{t2}^{U}}{dk}=-\frac{1}{k}<0,$ and $\frac{dw_{t6}\left( R_{t2}^{U} \right)}{dk}=\frac{2\sigma_{4}}{b^{2}\left( p-s \right)}>0$. Since $\frac{d\sigma_{5}}{dk}=-\frac{4\sigma_{4}^{2}e^{r_{c}}}{b^{5}\left( p-s \right)^{2}}<0$, we have $\frac{d\bar{\beta}\left( R_{t2}^{U} \right)}{dk}=\frac{2\left( b-a \right)b\left( p-s \right)\frac{d\sigma_{5}}{dk}}{\left[ \sigma_{4}+2\left( b-a \right)\sigma_{5} \right]^{2}}<0$. Since $\frac{dq_{3}\left( \underline{w}\left( r_{c} \right),r_{c},k \right)}{dk}=-\frac{\frac{b^{2}\left( p-s \right)^{2}}{2\left( b-a \right)}e^{r_{c}}}{\left( p-s \right)^{2}-\left( \frac{b\left( p+s \right)}{2\left( b-a \right)} \right)^{2}}<0$, we have $\frac{d\hat{\beta}\left( r_{c} \right)}{dk}=\frac{q_{2}\left( \underline{w}\left( r_{c} \right),r_{c} \right)\left( 1-\gamma\left( \underline{w}\left( r_{c} \right),r_{c} \right) \right)}{q_{3}\left( \underline{w}\left( r_{c} \right),r_{c},k \right)^{2}}\frac{dq_{3}\left( \underline{w}\left( r_{c} \right),r_{c},k \right)}{dk}<0$. And $\frac{dq_{0}\left( R_{t2}^{U},{\beta^{U}}^{*} \right)}{dk}=\frac{b^{2}\left( p-s \right)}{2\sigma_{4}}\frac{1}{\left( 1-\bar{\beta}\left( R_{t2}^{U} \right) \right)^{2}}\frac{d{\beta^{U}}^{*}}{dk}<0$.

Next, the retailer’s bankruptcy probability $\Pr\left[ \xi\leq\hat{\xi}\left( q_{0}\left( R_{t2}^{U},{\beta^{U}}^{*} \right) \right) \right]=\frac{\frac{b^{2}}{2\left( 1-{\beta^{U}}^{*} \right)}\left[ \frac{{\beta^{U}}^{*}}{2\left( b-a \right)}-\frac{s}{\sigma_{4}} \right]-a}{b-a}$ and then $\frac{d\Pr\left[ \xi\leq\hat{\xi}\left( q_{0}\left( R_{t2}^{U},{\beta^{U}}^{*} \right) \right) \right]}{dk}=\frac{b^{2}}{2\left( 1-{\beta^{U}}^{*} \right)^{2}}\left[ \frac{1}{2\left( b-a \right)}-\frac{s}{\sigma_{4}} \right]\left( \frac{d{\beta^{U}}^{*}}{dk} \right)<0$.

Finally, we have $\frac{dw_{t2}}{dk}=-\frac{\left( p-s \right)}{\sqrt{\left( bp-as \right)^{2}-4\left( b-a \right)\left( p-s \right)ke^{r_{f}}}}<0$ and $\frac{dq_{t2}}{dk}=\frac{\left( p-s \right)}{\sqrt{\left( bp-as \right)^{2}-4\left( b-a \right)\left( p-s \right)ke^{r_{f}}}}>0$.

Table B3. Three-parties’ Equilibrium Decisions for the BU-retailers and BB-retailers.

| **The type of the retailer** | $\boldsymbol{k}$ | ${\boldsymbol{w}^{\boldsymbol{U}}}^{\boldsymbol{*}}$ | ${\boldsymbol{q}^{\boldsymbol{U}}}^{\boldsymbol{*}}$ | $\boldsymbol{w}_{\boldsymbol{t}\boldsymbol{6}}\left( \boldsymbol{R}_{\boldsymbol{t}\boldsymbol{2}}^{\boldsymbol{U}} \right)$ | $\boldsymbol{w}_{\boldsymbol{s}\boldsymbol{1}}$ | $\boldsymbol{w}_{\boldsymbol{t}\boldsymbol{2}}$ | $\mathbb{E}\left[ \boldsymbol{\pi}_{\boldsymbol{s}}^{\mathbf{*}}\left( \boldsymbol{w}_{\boldsymbol{t}\boldsymbol{6}}\left( \boldsymbol{R}_{\boldsymbol{t}\boldsymbol{2}}^{\boldsymbol{U}} \right) \right) \right]$ | | $\mathbb{E}\left[ \boldsymbol{\pi}_{\boldsymbol{s}}^{\mathbf{*}}\boldsymbol{(}\boldsymbol{w}_{\boldsymbol{t}\boldsymbol{2}}\boldsymbol{)} \right]$ | $\boldsymbol{R}^{\boldsymbol{U}^{\boldsymbol{*}}}$ | $\left( \boldsymbol{\gamma}\left( \boldsymbol{r}_{\boldsymbol{c}} \right)\boldsymbol{,}\hat{\boldsymbol{\beta}}\left( \boldsymbol{r}_{\boldsymbol{c}} \right) \right]$ | | ${\boldsymbol{\beta}^{\boldsymbol{U}}}^{\boldsymbol{*}}$ | |
| --- | --- | --- | --- | --- | --- | --- | --- | --- | --- | --- | --- | --- | --- | --- |
| BU-retailer | 0.10 | **0.974** | 0.103 | 0.047 | 0.740 | **0.974** | -3.030 | | 0.050 | The retailer does not use IBF-B | | | | |
|  | 0.50 | **0.949** | 0.527 | 0.233 | 0.740 | **0.949** | -1.658 | | 0.241 |  |  |  |  |  |
|  | 0.90 | **0.923** | 0.975 | 0.420 | 0.740 | **0.923** | -0.460 | | 0.421 |  |  |  |  |  |
|  | 1.30 | **0.895** | 1.453 | 0.607 | 0.740 | **0.895** | 0.564 | | 0.585 |  |  |  |  |  |
|  | 1.35 | **0.630** | 5.121 | **0.630** | 0.740 | 0.891 | 0.679 | | 0.604 | 0.105 | [0.571,0.582] | | 0.582 | |
|  | 1.40 | **0.653** | 5.064 | **0.653** | 0.740 | 0.887 | 0.792 | | 0.624 | 0.069 | [0.571,0.577] | | 0.577 | |
|  | 1.45 | **0.677** | 5.006 | **0.677** | 0.740 | 0.884 | 0.902 | | 0.642 | 0.034 | [0.571,0.572] | | 0.572 | |
|  | $\boldsymbol{k}$ | ${\boldsymbol{w}^{\boldsymbol{U}}}^{\boldsymbol{*}}$ | ${\boldsymbol{q}^{\boldsymbol{U}}}^{\boldsymbol{*}}$ | $\boldsymbol{w}_{\boldsymbol{s}\boldsymbol{2}}$ | $\boldsymbol{w}_{\boldsymbol{t}\boldsymbol{4}}$ | $\boldsymbol{w}_{\boldsymbol{s}\boldsymbol{1}}$ | $\boldsymbol{w}_{\boldsymbol{t}\boldsymbol{2}}$ | $\mathbb{E}\left[ \boldsymbol{\pi}_{\boldsymbol{s}}^{\mathbf{*}}\boldsymbol{(}\boldsymbol{w}_{\boldsymbol{s}\boldsymbol{2}}\boldsymbol{)} \right]$ | $\mathbb{E}\left[ \boldsymbol{\pi}_{\boldsymbol{s}}^{\mathbf{*}}\boldsymbol{(}\boldsymbol{w}_{\boldsymbol{t}\boldsymbol{2}}\boldsymbol{)} \right]$ | $\boldsymbol{R}^{\boldsymbol{U}^{\boldsymbol{*}}}$ | | $\left( \boldsymbol{\gamma}\left( \boldsymbol{r}_{\boldsymbol{c}} \right)\boldsymbol{,}\hat{\boldsymbol{\beta}}\left( \boldsymbol{r}_{\boldsymbol{c}} \right) \right]$ | | ${\boldsymbol{\beta}^{\boldsymbol{U}}}^{\boldsymbol{*}}$ |
| BB-retailer | 1.50 | **0.880** | 1.699 | 0.664 | 0.725 | 0.740 | **0.880** | 0.551 | 0.659 | The retailer does not use IBF-B | | | | |
|  | 2.00 | **0.841** | 2.374 | 0.688 | 0.733 | 0.740 | **0.841** | 0.688 | 0.825 |  |  |  |  |  |
|  | 2.50 | **0.796** | 3.136 | 0.713 | 0.737 | 0.740 | **0.796** | 0.830 | 0.946 |  |  |  |  |  |
|  | 3.00 | **0.743** | 4.031 | 0.736 | 0.735 | 0.740 | **0.743** | 0.977 | 1.000 |  |  |  |  |  |
|  | 3.50 | **0.740** | 4.082 | 0.760 | 0.725 | **0.740** | 0.676 | 1.126 | 0.929 |  |  |  |  |  |
|  | 4.00 | **0.740** | 4.082 | 0.787 | 0.773 | **0.740** | 0.668 | 1.277 | 0.451 |  |  |  |  |  |

Table B4. Optimal loan menu for BU-retailers.

| The type of the retailer | $k$ | The lower bound of inventory advance rate  $\gamma\left( r_{c} \right)$ | The upper bound of inventory advance rate  $\hat{\beta}\left( r_{c} \right)$ | $\bar{\beta}\left( R_{t2}^{U} \right)$ | ${\beta^{U}}^{*}$ | $R^{U^{*}}$ |
| --- | --- | --- | --- | --- | --- | --- |
| BU-retailer | 0.10 | 0.571 | 0.673 | 0.956 | 0.673 | 2.708 |
|  | 0.50 | 0.571 | 0.648 | 0.940 | 0.648 | 1.099 |
|  | 0.90 | 0.571 | 0.620 | 0.906 | 0.620 | 0.511 |
|  | 1.30 | 0.571 | 0.586 | 0.786 | 0.586 | 0.143 |
|  | 1.35 | 0.571 | 0.582 | 0.745 | 0.582 | 0.105 |
|  | 1.40 | 0.571 | 0.577 | 0.684 | 0.577 | 0.069 |
|  | 1.45 | 0.571 | 0.572 | 0.586 | 0.572 | 0.034 |
