# Supplementary material for: Retailer’s inventory-based financing with bounded advance rates: Interplay between wholesale price contract and loan menu
Source: PLoS One. 2026 Jul 30;21(7):e0347675. doi: 10.1371/journal.pone.0347675 (PMC13423192; doi:10.1371/journal.pone.0347675)
Supplement: Attachment — Submitted filename: Review Comments.docx [file pone.0347675.s002.docx]

**Comments**

The research paper “Retailer’s Inventory-Based Financing with Bounded Advance Rates: Interplay between Wholesale Price Contract and Loan Menu” investigated a supply chain model comprises a capital constrained retailer, a bank, and a supplier. Specifically, a capital constrained retailer utilizing inventory-based financing to purchase products from the supplier by choosing from bank loan menu and wholesale price contracts by the supplier. The authors aim to help financial institutions to balance profit goals with the necessity of reducing credit risk. To this end, a mathematical framework was developed to assist in leveraging their physical assets to overcome common funding obstacles. However, there are minor concerns which need to be addressed for publication in PLOS One.

- The authors need to incorporate updated literature and create a distinction from closely related papers. For example, latest paper in **Table 1** is from 2020. Besides, studies like “Chen, H., & Zhu, W. (2025). Retailer-initiated inventory-based financing. *Available at SSRN 3974181”* needs to be considered to create a distinction from.
- The authors note that their optimal advance rate 24% - 41% which is quite low compared to the stated 50% - 65%. A suggestion to strengthen the managerial insight section by providing explicit explanations.
- A consistent writing style needs to be incorporated. For example, writing consistency for “subject to” or “s.t.” and abbreviations for IBF-B, SME etc.
- Please revisit the whole manuscript for typo errors.

Goodluck!
