# Supplementary material for: Retailer’s inventory-based financing with bounded advance rates: Interplay between wholesale price contract and loan menu
Source: PLoS One. 2026 Jul 30;21(7):e0347675. doi: 10.1371/journal.pone.0347675 (PMC13423192; doi:10.1371/journal.pone.0347675)
Supplement: Attachment — Submitted filename: Response to Reviewers.docx [file pone.0347675.s004.docx]

**Response to reviewers for “Retailer’s Inventory-Based Financing with Bounded Advance Rates: Interplay between Wholesale Price Contract and Loan Menu”**

Dear Editors:

Thank you for your email and for the constructive comments and suggestions provided by the reviewers regarding our manuscript entitled “Retailer’s Inventory-Based Financing with Bounded Advance Rates: Interplay between Wholesale Price Contract and Loan Menu” (ID: PONE-D-26-16015). We sincerely appreciate the time and effort that you and the reviewers have devoted to evaluating our work. The insightful feedback has been extremely helpful in identifying areas for improvement and enhancing overall quality of the manuscript.

We highly appreciate this revising opportunity and believe that the reviewers’ constructive suggestions will greatly help us improve the quality and clarity of our work. We have carefully addressed all concerns raised and have used them as guidance in the revised version of the manuscript.

Once again, we sincerely thank you and all the reviewers for your insightful comments, which have been instrumental in enhancing the quality of our work. We have carefully studied these suggestions, thoroughly revised the manuscript and made point-by-point corrections and improvements, which are marked in red in the revised manuscript. We hope that these revisions meet with your approval.

Yours sincerely,

The authors

**A point-by-point response to the reviewers’ comments**

# Review comments from Editor

## **Major concerns:**

1. **Clarification of Collateral Valuation and Liquidation Recovery Value**

***Suggestion 1: Critical Inconsistency in Liquidation Value.***

***The model states in Section 3.1 that "the bank has a legal right to liquidate the retailer's inventory at salvage value*** $\boldsymbol{s}$ ***in the event of default," and the entire bankruptcy analysis in Sections 4 and 6 uses*** $\boldsymbol{s}$ ***as the recovery value. However, Section 8.2 (Limitations) states: "We assume that the liquidation value of inventory equals the wholesale price." This is a direct contradiction. The collateral valuation is central to the model (the loan limit is*** $\boldsymbol{\beta wq}$***, which is based on wholesale price***$\boldsymbol{w}$***, while recovery is based on*** $\boldsymbol{s}$***), so the authors must clearly state and justify the liquidation value assumption in Section 3, not only as an afterthought limitation. If the liquidation value truly equals the wholesale price, the entire bankruptcy threshold structure would need revisiting.***

**Our response:** Thank you for your careful observation. We agree that the wording in Section 8.2 (Limitations) could cause confusion regarding the valuation of pledged inventory. We would like to clarify that the model distinguishes between the collateral value used for determining the loan limit and the liquidation recovery value obtained from unsold inventory.

Specifically, throughout the model, the loan limit is determined based on the inventory collateral value, which is measured by the wholesale value of inventory, i.e., $\beta wq$. In contrast, the bankruptcy analysis in Sections 4 and 6 incorporates the liquidation recovery value of unsold inventory. When demand is realized, the bank can recover the salvage value of the remaining inventory, yielding a recovery value of $s\left( q-\xi\right)^{+}$.

Section 3.1 has been revised to explicitly distinguish the collateral value and liquidation recovery value. For specific modification, please refer to the text highlighted in red in the **Paragraphs 2 and 3 of Section 3.1 on Pages 11-12.**

Since the statement in Section 8.2 (Limitations) referring to the “liquidation value” of inventory was imprecise, to avoid confusion, we have revised this terminology to “collateral value”, which is consistent with the loan-limit determination in the model. For specific modification, please refer to the text highlighted in red in the **Paragraph 1 of Section 8.2 on Page 42.** Theses revisions clarify the model assumptions.

We are grateful to the reviewer for pointing out this confusion and giving us the opportunity to provide further clarification.

2. **Correction of Numbering Mismatches**

***Suggestion 2: Fix the Numbering Mismatch Between Main Text and Appendix in the lemma proofs***

**Our response:** Thank you for pointing out this issue. We carefully checked the numbering of lemmas, propositions, equations, and related references throughout the manuscript and the appendix. Specifically, the content previously labeled as the Proof of Lemma 5 has been incorporated into the Proof of Lemma 4, where it properly belongs. Consequently, the previous Proof of Lemma 6 has been renumbered as the Proof of Lemma 5 and the numbering of the subsequent lemma proofs has been adjusted accordingly. All cross-references between the main text and the appendix have also been updated to ensure consistency.

For specific modification, please refer to the text highlighted in red in the **S1 Appendix A on Page 4.** Thank you once again for your careful review.

3. **Improvement of Figure Quality**

***Suggestion 3: Some of the figures can be enhanced with high-definition images.***

**Our response:** Thank you for this helpful suggestion. We have carefully reviewed all figures in the revised manuscript after converting the document to PDF. During this process, we found that Figure 3 exhibited insufficient clarity. Therefore, we regenerated and replaced Figure 3 with a higher-definition version to improve its readability and presentation quality. In addition, all figures have been prepared as separate TIFF files at 600dpi in accordance with the journal’s image requirements. The revised figure has been incorporated into the **Section 4.3 on Page 19 and its title has been highlighted in red.** Thank you once again for your guidance.

4. **Existence and Uniqueness of** $\boldsymbol{q}_{\boldsymbol{3}}\left( \boldsymbol{w,R,k} \right)$

***Suggestion 4: Implicit Fixed-Point Equation for*** $\boldsymbol{q}_{\boldsymbol{3}}\left( \boldsymbol{w,R,k} \right)$ ***Is Not Justified Lemma 3 defines*** $\boldsymbol{q}_{\boldsymbol{3}}\boldsymbol{(w, R, k)}$ ***as the solution to:***$\boldsymbol{q}_{\boldsymbol{3}}\left( \boldsymbol{w,R,k} \right)\boldsymbol{=}{\bar{\boldsymbol{F}}}^{\boldsymbol{-1}}\left( \frac{\boldsymbol{w}\boldsymbol{e}^{\boldsymbol{R}}\boldsymbol{-s}}{\boldsymbol{p-s}}\bar{\boldsymbol{F}}\left( \hat{\boldsymbol{\xi}}\left( \boldsymbol{q}_{\boldsymbol{3}}\left( \boldsymbol{w,R,k} \right) \right) \right) \right)\boldsymbol{.}$ ***This is an implicit fixed-point equation. The existence and uniqueness of a solution are never established. Given that the IFR assumption is used throughout, the authors should either (a) prove existence and uniqueness explicitly, or (b) cite a supporting result. Without this, the upper bound*** $\hat{\boldsymbol{\beta}}$ ***is not well-defined, which undermines the entire IBF-B framework.***

**Our response:** Thank you for this insightful comment. Following your suggestion, we have added a formal proof of the existence and uniqueness of $q_{3}\left( w,R,k \right)$ in S1 Appendix A. The proof shows that, under the IFR assumption adopted in the paper, the fixed-point equation admits a unique solution. Therefore, $q_{3}\left( w,R,k \right)$ is well-defined and the corresponding upper bound $\hat{\beta}$ used in the IBF-B framework exists and is uniquely determined. The detailed proof has been added to **S1 Appendix: A proof of Lemma 3 on Pages 1-2**. Thank you once again for your valuable guidance.

5. **Clarification of the Stackelberg Game Structure**

***Suggestion 5: Ambiguity in the Stackelberg Game Structure***

***The game structure is described inconsistently. Section 1.1 says "the supplier acts as the leader and the capital-constrained retailer functions as the follower," yet Assumption 3 states "the bank can act as a Stackelberg game leader, the supplier is a sub-leader and the capital-constrained retailer is a follower." Figure 1 shows the bank and supplier both acting at the beginning of the selling season, making it unclear whether the bank or the supplier moves first. The sequential structure needs to be specified precisely: Does the bank announce*** $\boldsymbol{(R, \beta)}$ ***before or after the supplier announces*** $\boldsymbol{w}$***? The current analysis in Section 6 treats*** $\boldsymbol{w}$ ***as given when the bank optimizes, which is inconsistent with the bank being the overall leader. This needs to be clarified both in the text and in Figure 1.***

**Our response:** Thank you very much for this valuable comment. We apologize for the confusion in the decision sequence among the bank, supplier and retailer caused by the description of the game structure in previous versions.

In the revised manuscript, we have clarified and unified the description of Stackelberg game structure. Specifically, the overall game is now consistently described as a three-level Stackelberg game in which the bank acts as the overall leader, the supplier acts as the sub-leader, and the capital-constrained retailer is the follower.

We have also clarified the roles of Sections 5-7 in Section 3.2. Since obtaining analytical solutions or closed-form expressions for the three-level Stackelberg game is impossible under newsvendor setting with general demand distribution, we study the supplier’s optimal wholesale price contract under a given loan contract in Section 5, while investigating the bank’s optimal loan menu under a given wholesale price. These analyses are intended to characterize the structural properties of the supplier’s pricing decision and the bank’s IBF-B decision under a general demand distribution. To further reveal the interaction between the loan menu and the wholesale price contract, we examine the complete three-level Stackelberg game under a uniform demand distribution. In this complete game, the bank first announces the loan menu, the supplier subsequently determines the wholesale price, the retailer finally making financing and ordering decisions.

For detailed modifications, please refer to the text highlighted in red in **the Paragraph 1 of Section 1.1 on Page 4, the Paragraph 1 of Section 3.1 on Page 11, Assumption 3 in Section 3.1 on Page 13, Section 3.2 on Pages 13-14, Figure 1 on Page 14, the Paragraph 1 of Section 5.1 on Page 20, the Paragraph 1 of Section 6 on Page 26 and the last Paragraph of Section 6.1 on Page 27.** Thank you once again for your guidance.

6. **Discussion of Contract Efficiency Results**

***Suggestion 6: Contract Efficiency Values Are Very Low and Require Discussion***

***Table 6 shows contract efficiency*** $\frac{\mathbb{E}\left[ \boldsymbol{\Pi}^{\boldsymbol{*}} \right]}{\mathbb{E}\left[ \boldsymbol{\Pi}_{\boldsymbol{cn}}^{\boldsymbol{*}} \right]}$ ***as low as 6.46%–26.96% for BU-retailers who do not use IBF-B. These values are extremely low by the standards of supply chain coordination literature. Even for retailers who do use IBF-B, efficiency is 77–81%. The authors are largely silent on why such severe inefficiency persists even with IBF-B, and whether these magnitudes are realistic or are artifacts of the specific parameter choices. A more substantive discussion—potentially with sensitivity analysis over key parameters—is required.***

**Our response:** Thank you for your professional comment. We have further discussed and explained the contract efficiency results.

First, we have revised the numerical setting to better reflect practical inventory categories, as explained in our response to Reviewer 2, Comment 2. Contract efficiencies remain relatively low for BU-retailers that do not adopt IBF-B, whereas it remains substantially higher for BU-retailers adopting IBF-B.

Second, we have strengthened the discussion of the relatively low contract efficiencies observed for poorer BU-retailers. In equilibrium, these retailers do not adopt IBF-B because the loan interest rates required to compensate for their high bankruptcy risk are prohibitively high (see detail in S1 Appendix B Table B4). Consequently, these retailers place orders by solely using limited working capital, resulting in substantially lower order quantities and then lower contract efficiency. We have added an explicit explanation of this result in the discussion of Table 6 **in lines 806-810 on Page 35.**

Third, we have further clarified why contract efficiency remains below the centralized benchmark even when IBF-B is adopted. Although IBF-B substantially alleviates financing constraints and improves supply chain performance, the supplier’s wholesale pricing decision, the retailer’s bankruptcy risk, and the bank’s risk-control considerations continue to affect both operational and financing decisions. We have added this explanation in the discussion of Table 6 **in lines 818-822, 824-828 on Page 36.** Therefore, inefficiencies persist relative to the centralized system.

Finally, following your suggestion, we have added sensitivity analyses with respect to the salvage-to-price ratio, demand spread and the bank’s loan cost rate. The results show that although contract efficiency varies with the salvage-to-price ratio, demand spread and the bank’s loan cost rate, the qualitative pattern of contract efficiency remains unchanged. In particular, contract efficiencies remain relatively low for BU-retailers that do not adopt IBF-B, whereas it remains substantially higher for BU-retailers adopting IBF-B. These additional analyses suggest that the efficiency levels reported in Table 6 are not driven by a particular parameter setting but reflect the interaction among capital constraints, financing and operational decisions. **The detailed discussion of the sensitivity analyses of contract efficiency is presented in Section 7.3.2 on Pages 38-39.**

7. **Sensitivity Analysis of Key Economic Parameters**

***Suggestion 7: No Sensitivity Analysis.***

***The paper uses a single numerical example throughout*** $\boldsymbol{(p=3, c=0.5, s=0.4,}\boldsymbol{r}_{\boldsymbol{f}}\boldsymbol{=0.02, U(0,10))}$***. There is no sensitivity analysis with respect to key parameters: monitoring cost*** $\boldsymbol{r}_{\boldsymbol{c}}$***, salvage value*** $\boldsymbol{s}$***, demand spread*** $\boldsymbol{(b-a)}$***, or retail price*** $\boldsymbol{p}$***. Given that the paper positions the bounded advance rate as an operational tool for banks, it is important to understand how the optimal bounds and loan menu change with the economic environment.***

**Our response:** Thank you for this valuable comment. Following this suggestion, we have added a comprehensive sensitivity analysis **in Section 7.3.** Specifically, we examine the impact of salvage-to-price ratio $\frac{s}{p}$, demand spread ($b-a$), and the bank’s loan cost rate $r_{c}$ on the bank’s optimal interest rates, optimal inventory advance rates, and the resulting contract efficiency under equilibrium decisions.

Instead of varying the salvage value and retailer price separately, we analyze the salvage-to-price ratio $\frac{s}{p}$, which directly captures inventory recoverability and plays an important role in the model. This treatment allows us to jointly reflect the effects of both the salvage value and the retail price on loan menus and supply chain contract efficiency. These additional analyses provide managerial insights into how the changes in inventory recoverability, demand situations, and financing costs affect the bank’s loan menus and supply chain performance.

For detailed modifications, please refer to the text highlighted in red in **Abstract, the Paragraph 4 of Section 1.1 on Page 6, the Paragraph 1 of Section 1.2 on Page 7, the Paragraph 4 of Section 2 on Page 9, the Paragraph 1 of Section 7 on Page 29, Section 7.3 on Pages 36-39, and the Paragraph 4 of Section 8.1 on Page 42.** Thank you once again for your guidance.

8. **Reference Formatting and Citation Clarifications**

***Suggestion 8: Reference Formatting Issues***

***Several references are inconsistently formatted: Reference [2] lists a DOI that appears to belong to a different paper (doi:10.1111/poms.13323 is listed for a 2022 IEEE Trans paper, but the same DOI is also used for Reference [35], the Fu et al. (2021) paper).***

***Reference [11] is a working paper from 2007 and is not published in a peer-reviewed venue. The paper relies on this work repeatedly; if it remains unpublished, the authors should verify that the results are not superseded by subsequent published work.***

***Reference [19] (Hu and Sobel 2005) is also an unpublished working paper. The authors should either cite the published version or clarify the current status.***

**Our response:** Thank you for your careful review of the references. First, we checked the reference list and corrected the DOI of Reference [2]. The DOI previously associated with Reference [2] was incorrect, whereas the DOI of Reference [35] is correct and has been verified. For detailed modifications, please refer to the text highlighted in red in **References on Page 43.**

Note that previous Reference [11] has been renumbered as Reference [14] in the revised manuscript. Regarding References [14] and [19], we verified their publication status. Reference [14] originates from a chapter of Jianer Zhou’s doctoral dissertation and has not subsequently appeared as a journal publication. Reference [19] is a well-known working paper in the inventory management literature and has likewise not been published in a peer-reviewed journal. We carefully reviewed the subsequent literature and did not identify subsequent published works that supersede the specific results cited in these papers. Moreover, both references continue to be cited in the inventory financing and supply chain finance literature. Therefore, we retain these references. Thank you once again for your valuable comment.

9. **Positioning the NB-Retailer Case as a Benchmark**

***Suggestion 9: The Role of the Supplier for NB-Retailer is Trivial***

***Section 5.2.1 (NB-retailer: retailer who orders using all working capital) does not use IBF-B and reduces to a standard "selling to the newsvendor" problem already studied by Lariviere and Porteus (2001). The results in Proposition 4 add little novelty. The authors should either (a) clearly position this case as a benchmark, or (b) reduce the space devoted to it and focus the analysis on the IBF-B-relevant cases (Sections 5.2.2–5.2.3).***

**Our response:** Thank you for this helpful suggestion. The NB-retailer case does not involve IBF-B and is closely related to the classical “selling to the newsvendor” problem already studied by Lariviere and Porteus (2001). Following this suggestion, we have explicitly positioned the NB-retailer case as a benchmark case in the revised manuscript. Although this case does not involve IBF-B, it provides a natural reference point for investigating how financing affects the supplier’s wholesale pricing decisions and the retailer’s ordering decisions in BB-retailer and BU-retailer cases. In particular, several equilibrium results under IBF-B are characterized related to this no-financing benchmark.

In addition, we have streamlined the discussion of Proposition 4 and reduced the space devoted to the NB-retailer case. More emphasis is now placed on the IBF-B-relevant cases (Sections 5.2.2–5.2.3).

For detailed modifications, please refer to the text highlighted in red in **Section 5.2.1 on Pages 23-24.** Thank you once again for your guidance.

10. **Strengthened comparison with Jiang and Liu [34]**

***Suggestion 10: Comparison with Jiang and Liu (2018) [34] Needs Strengthening***

***The paper [34] also studies inventory advance rates with wholesale price contracts. While Table 1 notes that [34] does not consider interest rates, the verbal distinction in the main text is insufficient. Section 2 should explicitly explain what analytical results this paper obtains that [34] does not, and why the simultaneous optimization of*** $\boldsymbol{(R, \beta)}$ ***yields qualitatively different insights.***

**Our response:** We greatly appreciate your valuable comments. Accordingly, we have strengthened the comparison with Jiang and Liu [34]. In addition to noting that the bank’s loan interest rate is exogenously given in [34], whereas it is endogenously determined in our work, we further clarify the differences in modeling assumptions and analytical results. Specifically, Jiang and Liu [34] focus on the impact of supplier overconfidence on inventory financing decisions and assume that the loan amount is equal to the loan limit.

In contrast, our work incorporates the retailer’s working capital into the financing and operational decisions and considers a rational supplier. Depending on the working capital levels, retailers exhibit different borrowing behaviors and bankruptcy risks.

In our framework, the inventory advance rate primarily determines the loan limit to the retailer and thereby directly influences the retailer’s order quantity. The interest rate reflects the compensation the bank requires for bearing credit risk. Our results show that the bank offers a higher inventory advance rate to encourage the retailer with lower working capital to order more, while simultaneously setting a higher interest rate to compensate for their greater default risk. By jointly optimizing both the interest rate $R$ and inventory advance rate $\beta$, the bank can balance profitability and credit risk. This dual-control mechanism creates endogenous interaction between financing and operational decisions, which cannot be captured when the interest rate is exogenously fixed. Consequently, the equilibrium outcomes for the retailer’s ordering and financing decisions, as well as the supplier’s wholesale pricing decision, differ qualitatively from the study where only $\beta$ is adjusted.

For detailed modifications, please refer to the text highlighted in red in **the lines 215-228 on Page 9.** Thank you once again for your guidance.

11. **Revision of Inventory Advance Rate Terminology**

***Suggestion 11: Definition of "Moderate" Advance Rate Is Circular***

***The paper defines IBF-B as the medium range*** $\boldsymbol{(\gamma,}\hat{\boldsymbol{\beta}}\boldsymbol{]}$***, and states the bank should set a "moderate advance rate" when the shadow price is non-positive. The term "moderate" is used to simultaneously describe the full feasible range*** $\boldsymbol{(\gamma,}\hat{\boldsymbol{\beta}}\boldsymbol{]}$ ***and a specific point*** $\bar{\boldsymbol{\beta}}$ ***within that range. This circular language creates confusion. The authors should use distinct terminology for the feasible range versus the specific optimal rate within it.***

**Our response:** Thank you for pointing out the ambiguity in the terminology. We have revised the manuscript to **replace the term “moderate advance rate” with “interior advance rate”** to clearly distinguish the medium range (a feasible range $(\gamma, \hat{\beta}]$) and the specific point $\bar{\beta}$ within this range. The medium range now consistently refers to feasible range, while interior advance rate denotes the specific value chosen $\bar{\beta}$ by the bank when the marginal contribution to profit is non-positive. The corresponding modifications in the main text have been highlighted **in red** for clarity. Thank you once again for your suggestions.

12. **Improved Interpretation of Table 3**

***Suggestion 12: Table 3 Is Difficult to Read***

***Table 3 (Optimal Order Quantity Corresponding to Wholesale Price) is cluttered and requires cross-referencing multiple earlier definitions to interpret. A brief verbal summary of each row's economic meaning would substantially improve accessibility.***

**Our response:** Thank you for this helpful suggestion. We have improved the readability and accessibility of Table 3 by adding a brief verbal explanation of the economic meaning with each row of Table 3. The additional discussion explains the different retailers’ order quantity and financing behavior under different wholesale price level. These explanations help readers understand the intuition behind the analytical results without repeatedly referring to earlier definitions and propositions.

For detailed modifications, please refer to the text highlighted in red in the **last Paragraph of Section 5.1 on Pages 22-23.** Thank you once again for your suggestion.

13. **Correction of Typographical Errors**

***Suggestion 13: Typographical Errors***

***The following typographical errors should be corrected:***

***Section 1.1, line 132: "enhancment of supply chain performance" → "enhancement of supply chain performance".***

***Appendix, Proof of Lemma 3: "the retailer's loan amount is constrained by the loam limit" → "loan limit".***

***Section 3.2, line 293: "We first analyze the retailer's optimal order quantity qr*" — the asterisk renders poorly; use consistent mathematical typesetting.***

***Section 1.1, line 72: "Since the decisions cannot made independently" → "cannot be made independently".***

***Section 5.2, line 493: the comma placement in "called NB-retailer; the retailer who intends to borrow below the loan limit, i.e.,*** $\underline{\boldsymbol{k}}\boldsymbol{\leq k\leq}\bar{\boldsymbol{k}}$***, called BB-retailer" omits the subject in the second clause.***

**Our response:** Thank you for pointing out these typographical errors. All identified issues have been corrected in the revised manuscript:

Section 1.1, Paragraph 4: “enhancment of supply chain performance” corrected to “enhancement of supply chain performance”.

Appendix, Proof of Lemma 3: “loam limit” corrected to “loan limit”.

Section 3.2, Paragraph 2: the asterisk in “$q_{r}^{*}$” has been properly formatted using consistent mathematical typesetting.

Section 1, Paragraph 6: “cannot made independently” corrected to “cannot be made independently”.

Section 5.2: the subject in the second clause has been added for clarity. In addition, the definitions of NB-, BB-, and BU-retailers have been revised for grammatical completeness and clarity. Specific modifications are highlighted in red **in the Paragraph 2 in Section 5.2 on Page 23.**

All corrections are highlighted **in red** in the revised manuscript. Thank you once again for your careful review.

14. **Summary of Lemma EC.1 in Main Text**

***Suggestion 14: Lemma EC.1 in the Appendix Is Not Labeled in the Main Text
Lemma EC.1 (Appendix B) is referenced in the main text (Section 7.1) but is not stated there. At minimum, the result should be summarized in the main text, since it is used to derive Proposition 8. Alternatively, a clear forward reference with an inline statement would help readers follow the argument.***

**Our response:** Thank you for this valuable suggestion. To improve readability and help readers follow the derivation of Proposition 8, we have added a brief summary of Lemma EC. 1 in Section 7.1 of the main text. Specifically, we summarize the relationship between the working capital cutoffs ($\underline{k}\left( R \right)$ and $\bar{k}\left( R \right)$) and the interest rate under the uniform demand distribution. We further explain the updated working-capital ranges corresponding to BB- and BU-retailers, as well as the interest-rate ranges under which these retailers are willing to consider IBF-B. This summary provides the key insights of Lemma EC.1 and clarifies its role in deriving Proposition 8, while the complete statement and proof remain in S1 Appendix B.

For detailed modifications, please refer to the text highlighted in red in **the Paragraph 2 of** **Section 7.1 on Page 30.** Thank you once again for your suggestion.

15. **Replacement of “Bargaining Advantage” with “Market Power”**

***Suggestion 15: The Bank's "Bargaining Advantage" Is Not Formally Modeled***

***The paper repeatedly asserts that the bank has a "bargaining advantage" over the retailer (Abstract, Sections 1, 3, Assumption 3). However, no formal bargaining model (e.g., Nash bargaining, take-it-or-leave-it offer) is specified. The bank is simply modeled as a Stackelberg leader. The term "bargaining advantage" is misleading and should either be formalized or replaced with "market power" or "first-mover advantage."***

**Our response:** Thank you for pointing out the ambiguity regarding the term “bargaining advantage”. We have revised the manuscript to replace all occurrences of “bargaining advantage” with “market power” to accurately reflect the bank’s role as a Stackelberg leader. This term, “market power”, better captures the bank’s strategic position without implying a formal bargaining model.

For detailed modifications, please refer to the text highlighted in red in **Abstract, the Paragraph 3 of Section 1.1 on Page 5, the Paragraphs 3, 5 and 7 of Section 2 on Pages 8-10, Assumption 3 in Section 3.1 on Page 13, Paragraph 2 of Section 8.1 on Page 40.** Thank you once again for your suggestion.

# Review comments from reviewer 1

## **Major concerns:**

1. **Clarification on the Demand Distribution Assumption**

***Suggestion 1: The demand distribution is assumed to be uniform. It's useful to consider general distribution in the extension.***

**Our response:** Thank you very much for this valuable comment. We would like to clarify that the main analysis in our paper is not restricted to the uniform demand distribution. In fact, the supplier’s wholesale pricing decision under a given loan contract is derived under general demand distribution framework.

Our original objective was to further determine the bank’s optimal loan menu based on the supplier’s wholesale pricing decision. However, under a general demand distribution, the resulting Stackelberg game becomes analytically intractable, making it difficult to obtain closed-form solutions for the bank’s optimal decisions. Therefore, instead of imposing additional restrictive assumptions, we examined the bank’s loan menu under a given wholesale price in the general demand distribution setting.

To further illustrate the interplay between the supplier’s wholesale price contract and the bank’s loan menu, we then considered the uniform distribution as a special case. This special case allows us to derive explicit analytical results and characterize the bank’s optimal financing decisions when acting as the Stackelberg leader. Therefore, the uniform distribution is introduced primarily for tractability and for providing additional managerial insights into the interaction between the supplier’s wholesale price contract and the bank’s loan menu, rather than as a fundamental assumption of the model.

To avoid possible misunderstanding, we have revised the manuscript to clarify the role of the general demand distribution and the purpose of introducing the uniform distribution in **Paragraph 2 of Section 3.2 on Page 14**. We greatly appreciate the reviewer’s insightful comment.

2. **Focus on Bank-Provided Inventory-Based Financing**

***Suggestion 2: It's better the compare supplier financing and bank financing in this study.***

**Our response:** Thank you for this valuable comment. A comparison between supplier financing and bank financing is indeed an interesting research question. However, the primary objective of this study is to investigate the inventory-based financing (IBF) provided by a bank and to characterize the interactions among the bank’s loan menu, the supplier’s wholesale price contract, and the retailer’s operational decisions. Accordingly, our analysis focuses on how the bank jointly determines the interest rate and inventory advance rate to control credit risk, how the supplier offers the wholesale price contract, and how these decisions affect supply chain performance.

In contrast, supplier financing involves a fundamentally different financing structure and incentive mechanism, where the supplier acts not only as an operational decision maker but also as a financing provider. Incorporating supplier financing would require a different modeling framework and substantially expand the scope of the study. Therefore, we focus on bank-provided IBF in the current paper. We agree that a systematic comparison between supplier financing and bank financing would provide additional managerial insights and consider it a promising direction for future research. Thank you once again for your suggestion.

3. **Practical Evidence for Bounded Inventory Advance Rates**

***Suggestion 3: The bounded rate should be added some evidence in the introduction.***

**Our response:** Thank you for your helpful suggestion. To provide practical evidence for the bounded inventory advance rate, we have revised the Introduction by incorporating industry examples of inventory-based financing (IBF). Specifically, we added evidence showing that inventory advance rates are typically subject to explicit upper limits imposed by lenders to control credit risk. For example, industry sources report inventory advance rates of around 50% and commonly within the range of 50%-65%. These examples provide practical support for the bounded inventory advance rates considered in our model. The corresponding discussion has been added to the **Paragraph 3 of Section 1 (Introduction) on Page 3**.

# Review comments from reviewer 2

## **Major concerns:**

1. **Updated Literature Review and Distinction from Related Studies**

***Suggestion 1: The authors need to incorporate updated literature and create a distinction from closely related papers. For example, latest paper in Table 1 is from 2020. Besides, studies like “Chen, H., & Zhu, W. (2025). Retailer-initiated inventory-based financing. Available at SSRN 3974181” needs to be considered to create a distinction from.***

**Our response:** Thank you for your valuable comments. In this revision, we have updated latest literature by incorporating several recent studies on inventory-based financing (IBF) and revised the Table 1 accordingly. Specifically, we added recent references such as Liang et al. (2025), Chen and Zhu (2025) and Song et al. (2026).

Following the reviewer’s suggestion, we also strengthened the discussion of closely related studies and clarified the distinctions between our work and the existing literature. In particular, we added a comparison with Chen and Zhu (2025), who study retailer-initiated IBF and derive the lender’s optimal interest rate with an exogenously specified inventory advance rate. In contrast, our study explicitly incorporates retailers working capital and jointly determines the interest rate and inventory advance rate, allowing us to characterize retailer classifications, financing behaviors, bankruptcy risks and the bounded inventory advance rates. The corresponding revisions have been made in lines **237-249** **on Pages 9-10 and Table 1 on Page 10.** The references are as follows.

Liang G, Zhang Z, Zhang L, Zhou J. Dynamic inventory management with inventory-based financing under stochastic market value of collateralized inventory. 2025. Available at SSRN 5528380. [http://dx.doi.org/10.2139/ssrn.5528380](https://dx.doi.org/10.2139/ssrn.5528380)

Chen H, Zhu W. Retailer initiated inventory-based financing. 2025. Available at SSRN 3974181. <http://dx.doi.org/10.2139/ssrn.3974181>

Song L, Guo X, Xu F, Chen Y. Smart contracts and inventory-based financing in supply chains. 2026. Available at SSRN 6091528. [http://dx.doi.org/10.2139/ssrn.6091528](https://dx.doi.org/10.2139/ssrn.6091528)

2. **Explanation of the Low Optimal Inventory Advance Rates**

***Suggestion 2: The authors note that their optimal advance rate 24% - 41% which is quite low compared to the stated 50% - 65%. A suggestion to strengthen the managerial insight section by providing explicit explanations.***

**Our response:** Thank you for this valuable suggestion. To better understand the discrepancy that the optimal inventory advance rate reported in the previous version (24%-41%) were substantially lower than the inventory advance rates commonly observed in practice, we found that the low advance rates were mainly driven by the conservative salvage-to-price ratio adopted in the original numerical example, which implied very limited collateral recovery value.

To better reflect inventory categories commonly accepted in IBF, such as consumer electronics^[1]^, household appliances^[2]^, and standardized retail products^[3]^, we revised the numerical example by setting $p=1$ and $s=0.4$, corresponding to a **salvage-to-price ratio of** $\frac{\boldsymbol{s}}{\boldsymbol{p}}\boldsymbol{=0.4}$. Under this setting, **the optimal advance rate increases to 57.1%-67.3%,** which is broadly consistent with the 50%-65% range reported in industry practice. The detailed numerical results are shown **in S1 Appendix B Tables B3-B4 and the detailed modifications in the main manuscript are highlighted in the line 831 on Page 36.**

In addition, we strengthened the managerial discussion by explaining how salvage-to-price ration affects the bank’s optimal inventory advance rate and interest rate. The corresponding revisions have been shown in **Section 7.3 (Sensitivity analysis)**.

Accordingly, **Figure 6 and Table 6 presented in Section 7** have been recalculated and updated based on the revised parameter setting. Despites the changes in numerical values, the qualitative patterns and managerial insights derived from Figure 6 and Table 6 remain unchanged. Therefore, the corresponding discussions in the previous version are still applicable and have been preserved in the revised manuscript.

3. **Writing Consistency and Terminology Standardization**

***Suggestion 3: A consistent writing style needs to be incorporated. For example, writing consistency for “subject to” or “s.t.” and abbreviations for IBF-B, SME etc.***

**Our response:** Thank you for your careful review. Following your suggestion, we have conducted a thorough check of the manuscript to improve writing consistency throughout the paper. Specifically, we standardized the use of terminology, abbreviations, and notations. For example, expressions such as “subject to” and “s.t.” have been unified, and abbreviations including IBF-B and SME are now used consistently throughout the manuscript. We have also carefully reviewed the manuscript to ensure consistency in mathematical notation and presentation style.

4. **Correction of Typographical Errors**

***Suggestion 4: Please revisit the whole manuscript for typo errors.***

**Our response:** Thank you for your careful review. Following your suggestion, we have thoroughly proofread the entire manuscript and corrected typographical, grammatical, and formatting errors throughout the paper. In addition to the errors specifically identified in Editor Comment 13, we further revised several issues discovered during the proofreading process.

For example, we corrected a subject-verb agreement error in **Section 1 line 59** by revising “how the lender determine the advance rate” to “how the lender determines the advance rate” and clarify the expression by replacing “the up-stream” with “the upstream supplier”.

We also refined several expressions to improve readability and linguistic accuracy. For instance, in **Section 1.1 line 112,** we revised “trade off between…” to the more appropriate expression “make a trade-off between…”. In addition, the sentence in **Section 1.1 lines 142-144**, “The retailer who borrows up to the loan limit is poor, and the loan he obtains is large, which in turn the enhancement of supply chain performance is remarkable” was restricted as “The retailer who borrows up to the loan limit is poor and obtains a sizable loan, which in turn lead to a remarkable enhancement in supply chain performance” to improve both grammatical correctness and clarity of interpretation.

We also corrected grammatical inconsistencies in the definitions of NB-, BB-, and BU-retailers in **the Paragraph 2 of Section 5.2**, to ensure that the three retailer types are defined in complete and consistent manner. We have also carefully reviewed the manuscript to improve overall readability and presentation.

The corresponding revisions have been incorporated throughout the manuscript and **highlighted** **in red**. Thank you once again for your suggestion.

1. <https://www.investopedia.com/consumer-products-that-lose-value-rapidly-11680787>?
2. https://fiaxlo.com/appliance-depreciation-per-year/?
3. https://www.returnpro.com/resources/blog/retailers-know-returns-drive-value-so-why-is-recovery-still-failing?
