# Supplementary material for: Retailer’s inventory-based financing with bounded advance rates: Interplay between wholesale price contract and loan menu
Source: PLoS One. 2026 Jul 30;21(7):e0347675. doi: 10.1371/journal.pone.0347675 (PMC13423192; doi:10.1371/journal.pone.0347675)
Supplement: Peer review history

# Peer review: Decision Letter 0

**DOI:** 10.1371/journal.pone.0347675.r001

**Authors:** Bo Li

13 May 2026

PONE-D-26-16015

Retailer’s Inventory-Based Financing with Bounded Advance Rates: Interplay between Wholesale Price Contract and Loan Menu

PLOS One

Dear Dr. Tian,

Thank you for submitting your manuscript to PLOS ONE. After careful consideration, we feel that it has merit but does not fully meet PLOS ONE’s publication criteria as it currently stands. Therefore, we invite you to submit a revised version of the manuscript that addresses the points raised during the review process.

Please submit your revised manuscript by Jun 25 2026 11:59PM. If you will need more time than this to complete your revisions, please reply to this message or contact the journal office at plosone@plos.org. When you're ready to submit your revision, log on to https://www.editorialmanager.com/pone/ and select the 'Submissions Needing Revision' folder to locate your manuscript file.

Please include the following items when submitting your revised manuscript:

- A letter that responds to each point raised by the academic editor and reviewer(s). You should upload this letter as a separate file labeled 'Response to Reviewers'.
- A marked-up copy of your manuscript that highlights changes made to the original version. You should upload this as a separate file labeled 'Revised Manuscript with Track Changes'.
- An unmarked version of your revised paper without tracked changes. You should upload this as a separate file labeled 'Manuscript'.

If you would like to make changes to your financial disclosure, please include your updated statement in your cover letter. Guidelines for resubmitting your figure files are available below the reviewer comments at the end of this letter.

If applicable, we recommend that you deposit your laboratory protocols in protocols.io to enhance the reproducibility of your results. Protocols.io assigns your protocol its own identifier (DOI) so that it can be cited independently in the future. For instructions see: https://journals.plos.org/plosone/s/submission-guidelines#loc-laboratory-protocols. Additionally, PLOS ONE offers an option for publishing peer-reviewed Lab Protocol articles, which describe protocols hosted on protocols.io. Read more information on sharing protocols at https://plos.org/protocols?utm_medium=editorial-email&utm_source=authorletters&utm_campaign=protocols.

As the corresponding author, your ORCID iD is verified in the submission system and will appear in the published article. PLOS supports the use of ORCID, and we encourage all coauthors to register for an ORCID iD and use it as well. Please encourage your coauthors to verify their ORCID iD within the submission system before final acceptance, as unverified ORCID iDs will not appear in the published article. *Only*  the individual author can complete the verification step; PLOS staff *cannot*  verify ORCID iDs on behalf of authors.

We look forward to receiving your revised manuscript.

Kind regards,

Bo Li, Ph.D.

Academic Editor

PLOS One

Journal Requirements:

When submitting your revision, we need you to address these additional requirements.

1. Please ensure that your manuscript meets PLOS ONE's style requirements, including those for file naming. The PLOS ONE style templates can be found at

https://journals.plos.org/plosone/s/file?id=wjVg/PLOSOne_formatting_sample_main_body.pdf and

https://journals.plos.org/plosone/s/file?id=ba62/PLOSOne_formatting_sample_title_authors_affiliations.pdf

2. Thank you for stating in your Funding Statement:

“This work was supported by the National Natural Science Foundation of China (Grant Nos. 71972158, 71571147).”

Please provide an amended statement that declares *all* the funding or sources of support (whether external or internal to your organization) received during this study, as detailed online in our guide for authors at http://journals.plos.org/plosone/s/submit-now. Please also include the statement “There was no additional external funding received for this study.” in your updated Funding Statement.

Please include your amended Funding Statement within your cover letter. We will change the online submission form on your behalf.

3. Thank you for stating the following financial disclosure:

“This work was supported by the National Natural Science Foundation of China (Grant Nos. 71972158, 71571147).”

Please state what role the funders took in the study. If the funders had no role, please state: "The funders had no role in study design, data collection and analysis, decision to publish, or preparation of the manuscript."

If this statement is not correct you must amend it as needed.

Please include this amended Role of Funder statement in your cover letter; we will change the online submission form on your behalf.

4. If the reviewer comments include a recommendation to cite specific previously published works, please review and evaluate these publications to determine whether they are relevant and should be cited. There is no requirement to cite these works unless the editor has indicated otherwise.

5. Please review your reference list to ensure that it is complete and correct. If you have cited papers that have been retracted, please include the rationale for doing so in the manuscript text, or remove these references and replace them with relevant current references. Any changes to the reference list should be mentioned in the rebuttal letter that accompanies your revised manuscript. If you need to cite a retracted article, indicate the article’s retracted status in the References list and also include a citation and full reference for the retraction notice.

Additional Editor Comments:

This paper studies a three-party supply chain consisting of a capital-constrained retailer, a supplier, and a bank. The retailer uses inventory-based financing (IBF) to procure products from the supplier, using purchased inventory as collateral. The central contribution is the derivation of upper and lower bounds for the inventory advance rate—defining what the authors call IBF with bounded advance rates (IBF-B)—and an analysis of the interplay between the bank's loan menu (interest rate and advance rate) and the supplier's wholesale price contract. The paper is set up as a Stackelberg game and solved using a newsvendor framework and KKT conditions.

Given the comments of two knowledgeable reviewers, I invite the authors to submit a revised manuscript. Please address the issues raised by the two reviewers and my additional comments below.

The topic is relevant and the general research design is sound. However, the manuscript has significant issues across mathematical consistency, notational clarity, presentation, and certain gaps in the analysis that must be addressed before publication. These are detailed below.

1. Critical Inconsistency in Liquidation Value

The model states in Section 3.1 that "the bank has a legal right to liquidate the retailer's inventory at salvage value s in the event of default," and the entire bankruptcy analysis in Sections 4 and 6 uses s as the recovery value. However, Section 8.2 (Limitations) states: "We assume that the liquidation value of inventory equals the wholesale price." This is a direct contradiction. The collateral valuation is central to the model (the loan limit is βwq, which is based on wholesale price w, while recovery is based on s), so the authors must clearly state and justify the liquidation value assumption in Section 3, not only as an afterthought limitation. If the liquidation value truly equals the wholesale price, the entire bankruptcy threshold structure would need revisiting.

2. Fix the Numbering Mismatch Between Main Text and Appendix in the lemma proofs

3. Some of the figures can be enhanced with high-definition images.

4. Implicit Fixed-Point Equation for q₃ Is Not Justified

Lemma 3 defines q₃(w, R, k) as the solution to:

q₃ = F̄⁻¹( [(w·e^R − s)/(p − s)] · F̄(ξ̂(q₃)) )

This is an implicit fixed-point equation. The existence and uniqueness of a solution are never established. Given that the IFR assumption is used throughout, the authors should either (a) prove existence and uniqueness explicitly, or (b) cite a supporting result. Without this, the upper bound β̂ is not well-defined, which undermines the entire IBF-B framework.

5. Ambiguity in the Stackelberg Game Structure

The game structure is described inconsistently. Section 1.1 says "the supplier acts as the leader and the capital-constrained retailer functions as the follower," yet Assumption 3 states "the bank can act as a Stackelberg game leader, the supplier is a sub-leader and the capital-constrained retailer is a follower." Figure 1 shows the bank and supplier both acting at the beginning of the selling season, making it unclear whether the bank or the supplier moves first. The sequential structure needs to be specified precisely: Does the bank announce (R, β) before or after the supplier announces w? The current analysis in Section 6 treats w as given when the bank optimizes, which is inconsistent with the bank being the overall leader. This needs to be clarified both in the text and in Figure 1.

6. Contract Efficiency Values Are Very Low and Require Discussion

Table 6 shows contract efficiency (E[Π*]/E[Πcn*]) as low as 6.46%–26.96% for BU-retailers who do not use IBF-B. These values are extremely low by the standards of supply chain coordination literature. Even for retailers who do use IBF-B, efficiency is 77–81%. The authors are largely silent on why such severe inefficiency persists even with IBF-B, and whether these magnitudes are realistic or are artifacts of the specific parameter choices. A more substantive discussion—potentially with sensitivity analysis over key parameters—is required.

7. No Sensitivity Analysis

The paper uses a single numerical example throughout (p=3, c=0.5, s=0.4, r_f=0.02, U(0,10)). There is no sensitivity analysis with respect to key parameters: monitoring cost r_c, salvage value s, demand spread (b−a), or retail price p. Given that the paper positions the bounded advance rate as an operational tool for banks, it is important to understand how the optimal bounds and loan menu change with the economic environment.

8. Reference Formatting Issues

Several references are inconsistently formatted:

Reference [2] lists a DOI that appears to belong to a different paper (doi:10.1111/poms.13323 is listed for a 2022 IEEE Trans paper, but the same DOI is also used for Reference [35], the Fu et al. (2021) paper).

Reference [11] is a working paper from 2007 and is not published in a peer-reviewed venue. The paper relies on this work repeatedly; if it remains unpublished, the authors should verify that the results are not superseded by subsequent published work.

Reference [19] (Hu and Sobel 2005) is also an unpublished working paper. The authors should either cite the published version or clarify the current status.

9. The Role of the Supplier for NB-Retailer is Trivial

Section 5.2.1 (NB-retailer: retailer who orders using all working capital) does not use IBF-B and reduces to a standard "selling to the newsvendor" problem already studied by Lariviere and Porteus (2001). The results in Proposition 4 add little novelty. The authors should either (a) clearly position this case as a benchmark, or (b) reduce the space devoted to it and focus the analysis on the IBF-B-relevant cases (Sections 5.2.2–5.2.3).

10. Comparison with Jiang and Liu (2018) [34] Needs Strengthening

The paper [34] also studies inventory advance rates with wholesale price contracts. While Table 1 notes that [34] does not consider interest rates, the verbal distinction in the main text is insufficient. Section 2 should explicitly explain what analytical results this paper obtains that [34] does not, and why the simultaneous optimization of (R, β) yields qualitatively different insights.

11. Definition of "Moderate" Advance Rate Is Circular

The paper defines IBF-B as the medium range (γ, β̂], and states the bank should set a "moderate advance rate" when the shadow price is non-positive. The term "moderate" is used to simultaneously describe the full feasible range (γ, β̂] and a specific point β̄ within that range. This circular language creates confusion. The authors should use distinct terminology for the feasible range versus the specific optimal rate within it.

12. Table 3 Is Difficult to Read

Table 3 (Optimal Order Quantity Corresponding to Wholesale Price) is cluttered and requires cross-referencing multiple earlier definitions to interpret. A brief verbal summary of each row's economic meaning would substantially improve accessibility.

13. Typographical Errors

The following typographical errors should be corrected:

Section 1.1, line 132: "enhancment ofsupply chain performance" → "enhancement of supply chain performance"

Appendix, Proof of Lemma 3: "the retailer's loan amount is constrained by the loam limit" → "loan limit"

Section 3.2, line 293: "We first analyze the retailer's optimal order quantity qr*" — the asterisk renders poorly; use consistent mathematical typesetting

Section 3.1, line 72: "Since the decisions cannot made independently" → "cannot be made independently"

Section 5.2, line 493: the comma placement in "called NB-retailer; the retailer who intends to borrow below the loan limit, i.e., k≤k≤k̄, called BB-retailer" omits the subject in the second clause

14. Lemma EC.1 in the Appendix Is Not Labeled in the Main Text

Lemma EC.1 (Appendix B) is referenced in the main text (Section 7.1) but is not stated there. At minimum, the result should be summarized in the main text, since it is used to derive Proposition 8. Alternatively, a clear forward reference with an inline statement would help readers follow the argument.

15. The Bank's "Bargaining Advantage" Is Not Formally Modeled

The paper repeatedly asserts that the bank has a "bargaining advantage" over the retailer (Abstract, Sections 1, 3, Assumption 3). However, no formal bargaining model (e.g., Nash bargaining, take-it-or-leave-it offer) is specified. The bank is simply modeled as a Stackelberg leader. The term "bargaining advantage" is misleading and should either be formalized or replaced with "market power" or "first-mover advantage."

[Note: HTML markup is below. Please do not edit.]

Reviewers' comments:

Reviewer's Responses to Questions

**Comments to the Author**

1. Is the manuscript technically sound, and do the data support the conclusions?

The manuscript must describe a technically sound piece of scientific research with data that supports the conclusions. Experiments must have been conducted rigorously, with appropriate controls, replication, and sample sizes. The conclusions must be drawn appropriately based on the data presented.

Reviewer #1: Yes

Reviewer #2: Yes

**********

2. Has the statistical analysis been performed appropriately and rigorously?

Reviewer #1: N/A

Reviewer #2: Yes

**********

3. Have the authors made all data underlying the findings in their manuscript fully available?

The PLOS Data policy requires authors to make all data underlying the findings described in their manuscript fully available without restriction, with rare exception (please refer to the Data Availability Statement in the manuscript PDF file). The data should be provided as part of the manuscript or its supporting information, or deposited to a public repository. For example, in addition to summary statistics, the data points behind means, medians and variance measures should be available. If there are restrictions on publicly sharing data—e.g. participant privacy or use of data from a third party—those must be specified.

Reviewer #1: Yes

Reviewer #2: Yes

**********

4. Is the manuscript presented in an intelligible fashion and written in standard English?

PLOS ONE does not copyedit accepted manuscripts, so the language in submitted articles must be clear, correct, and unambiguous. Any typographical or grammatical errors should be corrected at revision, so please note any specific errors here.

Reviewer #1: Yes

Reviewer #2: Yes

**********

5. Review Comments to the Author

Please use the space provided to explain your answers to the questions above. You may also include additional comments for the author, including concerns about dual publication, research ethics, or publication ethics. (Please upload your review as an attachment if it exceeds 20,000 characters)

Reviewer #1: After reviewing this paper, I have following comments:

Comment 1: The demand distribution is assumed to be uniform. It's useful to consider general distribution in the extension.

Comment 2: It's better the compare supplier financing and bank financing in this study.

Comment 3: The bounded rate should be added some evidence in the introduction.

Reviewer #2: The research paper “Retailer’s Inventory-Based Financing with Bounded Advance Rates: Interplay between Wholesale Price Contract and Loan Menu” investigated a supply chain model comprises a capital constrained retailer, a bank, and a supplier. Specifically, a capital constrained retailer utilizing inventory-based financing to purchase products from the supplier by choosing from bank loan menu and wholesale price contracts by the supplier. The authors aim to help financial institutions to balance profit goals with the necessity of reducing credit risk. To this end, a mathematical framework was developed to assist in leveraging their physical assets to overcome common funding obstacles.

Please see the attached file for review comments.

**********

6. PLOS authors have the option to publish the peer review history of their article (what does this mean?). If published, this will include your full peer review and any attached files.

If you choose “no”, your identity will remain anonymous but your review may still be made public.

**Do you want your identity to be public for this peer review?**  For information about this choice, including consent withdrawal, please see our Privacy Policy.

Reviewer #1: No

Reviewer #2: No

**********

[NOTE: If reviewer comments were submitted as an attachment file, they will be attached to this email and accessible via the submission site. Please log into your account, locate the manuscript record, and check for the action link "View Attachments". If this link does not appear, there are no attachment files.]

To ensure your figures meet our technical requirements, please review our figure guidelines: https://journals.plos.org/plosone/s/figures

You may also use PLOS’s free figure tool, NAAS, to help you prepare publication quality figures: https://journals.plos.org/plosone/s/figures#loc-tools-for-figure-preparation.

NAAS will assess whether your figures meet our technical requirements by comparing each figure against our figure specifications.

Attachment Submitted filename: Review Comments.docx

# Author comment: Author response to Decision Letter 1

**DOI:** 10.1371/journal.pone.0347675.r002

15 Jun 2026

Thank you very much for the valuable comments and suggestions provided by the editor and reviewers. We have carefully addressed all comments and revised the manuscript accordingly. Detailed point-by-point responses are provided in the attached Response to Reviewers, and all corresponding revisions have been highlighted in red in the revised manuscript.

Attachment Submitted filename: Response to Reviewers.docx

# Editor report: Decision Letter 1

**DOI:** 10.1371/journal.pone.0347675.r003

**Authors:** Bo Li

16 Jun 2026

Retailer’s Inventory-Based Financing with Bounded Advance Rates: Interplay between Wholesale Price Contract and Loan Menu

PONE-D-26-16015R1

Dear Dr. Tian,

We’re pleased to inform you that your manuscript has been judged scientifically suitable for publication and will be formally accepted for publication once it meets all outstanding technical requirements.

Within one week, you’ll receive an e-mail detailing the required amendments. When these have been addressed, you’ll receive a formal acceptance letter and your manuscript will be scheduled for publication.

An invoice will be generated when your article is formally accepted. Please note, if your institution has a publishing partnership with PLOS and your article meets the relevant criteria, all or part of your publication costs will be covered. Please make sure your user information is up-to-date by logging into Editorial Manager at Editorial Manager® and clicking the ‘Update My Information' link at the top of the page. For questions related to billing, please contact billing support.

If your institution or institutions have a press office, please notify them about your upcoming paper to help maximize its impact. If they’ll be preparing press materials, please inform our press team as soon as possible -- no later than 48 hours after receiving the formal acceptance. Your manuscript will remain under strict press embargo until 2 pm Eastern Time on the date of publication. For more information, please contact onepress@plos.org.

Kind regards,

Bo Li, Ph.D.

Academic Editor

PLOS One

Additional Editor Comments (optional):

In Section 1.1, the authors state that a sizable loan "in turn lead to a remarkable enhancement in supply chain performance". This contains a subject-verb agreement error and should be corrected to "leads to a remarkable enhancement."

In Section 1.2, the phrase "other typical literature" is used when introducing Table 1. This would read much more professionally as "closely related literature" or "prior seminal literature."

Reviewers' comments:

# Editor report: Acceptance letter

**DOI:** 10.1371/journal.pone.0347675.r004

**Authors:** Bo Li

PONE-D-26-16015R1

PLOS One

Dear Dr. Tian,

I'm pleased to inform you that your manuscript has been deemed suitable for publication in PLOS One. Congratulations! Your manuscript is now being handed over to our production team.

At this stage, our production department will prepare your paper for publication. This includes ensuring the following:

* All references, tables, and figures are properly cited

* All relevant supporting information is included in the manuscript submission,

* There are no issues that prevent the paper from being properly typeset

You will receive further instructions from the production team, including instructions on how to review your proof when it is ready. Please keep in mind that we are working through a large volume of accepted articles, so please give us a few days to review your paper and let you know the next and final steps.

Lastly, if your institution or institutions have a press office, please let them know about your upcoming paper now to help maximize its impact. If they'll be preparing press materials, please inform our press team within the next 48 hours. Your manuscript will remain under strict press embargo until 2 pm Eastern Time on the date of publication. For more information, please contact onepress@plos.org.

You will receive an invoice from PLOS for your publication fee after your manuscript has reached the completed accept phase. If you receive an email requesting payment before acceptance or for any other service, this may be a phishing scheme. Learn how to identify phishing emails and protect your accounts at https://explore.plos.org/phishing.

If we can help with anything else, please email us at customercare@plos.org.

Thank you for submitting your work to PLOS ONE and supporting open access.

Kind regards,

PLOS ONE Editorial Office Staff

on behalf of

Professor Bo Li

Academic Editor

PLOS One
